# Genome-wide association analyses of borderline personality disorder identify 11 loci and highlight shared risk with mental and somatic disorders

**DOI:** 10.1038/s41588-026-02654-3

**Published:** 2026-07-20

**Authors:** Fabian Streit, Swapnil Awasthi, Alisha S. M. Hall, Alice Braun, Maria Niarchou, Eirini Marouli, Oladapo Babajide, Josef Frank, Lea Zillich, Carolin M. Callies, Diana Avetyan, Eric Zillich, Joonas Naamanka, Jean Gonzalez, Arvid Harder, Yi Lu, Zouhair Aherrahrou, Zain-Ul-Abideen Ahmad, Helga Ask, Anthony Batzler, Michael E. Benros, Odette M. Brand-de Wilde, Søren Brunak, Mie T. Bruun, Lea A. N. Christoffersen, Lucía Colodro-Conde, Brandon J. Coombes, Elizabeth C. Corfield, Norbert Dahmen, Maria Didriksen, Khoa M. Dinh, Srdjan Djurovic, Joseph Dowsett, Ole Kristian Drange, Helene Dukal, Susanne Edelmann, Christian Erikstrup, Mariana K. Espinola, Eva Fassbinder, Annika Faucon, Diana S. Ferreira de Sá, Jerome C. Foo, Maria Gilles, Alfonso Gutiérrez-Zotes, Thomas F. Hansen, Magnus Haraldsson, R. Patrick Harper, Alexandra Havdahl, Urs Heilbronner, Stefan Herms, Henrik Hjalgrim, Christopher Hübel, Gitta A. Jacob, Bitten Aagaard, Anders Jorgensen, Martin Jungkunz, Nikolaus Kleindienst, Nora Knoblich, Stefanie Koglin, Julia Kraft, Kristi Krebs, Christopher W. Lee, Yuhao Lin, Stefanie Lis, Amanda Lisoway, Ioannis A. Malogiannis, Amy Martinsen, Tolou Maslahati, Katharina Merz, Andreas Meyer-Lindenberg, Susan Mikkelsen, Christina Mikkelsen, Arian Mobascher, Gerard Muntané, Asmundur Oddsson, Sisse R. Ostrowski, Teemu Palviainen, Ole B. V. Pedersen, Geir Pedersen, Liam Quinn, Matthias A. Reinhard, Florian A. Ruths, Björn H. Schott, Michael Schredl, Emanuel Schwarz, Cornelia E. Schwarze, Michael Schwinn, Tabea Sarah Send, Engilbert Sigurdsson, Katja Simon-Keller, Astros T. Skuladottir, Joaquim Soler, Anne Sonley, Erik Sørensen, Hreinn Stefansson, Peter Straub, Jaana Suvisaari, Martin Tesli, Jacob Træholt, Henrik Ullum, Maja P. Völker, G. Bragi Walters, Rujia Wang, Christian C. Witt, Gerhard Zarbock, Peter Zill, John-Anker Zwart, DBDS Genomic Consortium, Søren Brunak, Mie T. Bruun, Lea A. N. Christoffersen, Maria Didriksen, Khoa M. Dinh, Joseph Dowsett, Ole Kristian Drange, Susanne Edelmann, Christian Erikstrup, Thomas F. Hansen, Henrik Hjalgrim, Bitten Aagaard, Susan Mikkelsen, Christina Mikkelsen, Sisse R. Ostrowski, Ole B. V. Pedersen, Liam Quinn, Michael Schwinn, Erik Sørensen, Hreinn Stefansson, Henrik Ullum, Kari Stefansson, Thomas Werge, the GLAD Study, Zain-Ul-Abideen Ahmad, Christopher Hübel, Yuhao Lin, Rujia Wang, Gerome Breen, HUNT All-In Psychiatry, Ole A. Andreassen, Ole A. Andreassen, Arnoud Arntz, Joanna M. Biernacka, Martin Bohus, Gerome Breen, Alexander L. Chapman, Sven Cichon, Lea K. Davis, Michael Deuschle, Sebastian Euler, Sabine C. Herpertz, Benjamin Hummelen, Andrea Jobst, Jaakko Kaprio, James L. Kennedy, Kelli Lehto, Klaus Lieb, Lourdes Martorell, Shelley McMain, Richard Musil, Vanessa Nieratschker, Markus M. Nöthen, Frank Padberg, Aarno Palotie, Juan C. Pascual, Nader Perroud, Josep A. Ramos-Quiroga, Ted Reichborn-Kjennerud, Marta Ribases, Stefan Roepke, Dan Rujescu, Sandra Sanchez-Roige, Claudia Schilling, Christian Schmahl, Kari Stefansson, Thorgeir E. Thorgeirsson, Gustavo Turecki, Elisabet Vilella, Thomas Werge, Bendik S. Winsvold, Johannes Wrege, Marcella Rietschel, Stephan Ripke, Stephanie H. Witt

**Affiliations:** 1 Hector Institute for Artificial Intelligence in Psychiatry, Central Institute of Mental Health, Medical Faculty Mannheim, Heidelberg University, Mannheim, Germany; 2 Department of Psychiatry and Psychotherapy, Central Institute of Mental Health, Medical Faculty Mannheim, Heidelberg University, Mannheim, Germany; 3 Department of Genetic Epidemiology in Psychiatry, Central Institute of Mental Health, Medical Faculty Mannheim, Heidelberg University, Mannheim, Germany; 4 German Center for Mental Health (DZPG), Partner Site Mannheim-Heidelberg-Ulm, Mannheim-Heidelberg-Ulm, Germany; 5 Department of Psychiatry and Psychotherapy, Charité Universitätsmedizin Berlin, Berlin, Germany; 6 Stanley Center for Psychiatric Research, Broad Institute of MIT and Harvard, Cambridge, MA USA; 7 Department of Clinical Medicine, Aarhus University, Aarhus, Denmark; 8 Department of Affective Disorders, Aarhus University Hospital-Psychiatry, Aarhus, Denmark; 9 Department of Medicine, Division of Genetic Medicine, Vanderbilt University Medical Center, Nashville, TN USA; 10 William Harvey Research Institute, Faculty of Medicine and Dentistry, Queen Mary University London, London, UK; 11 Hector Institute for Translational Brain Research, Central Institute of Mental Health, Medical Faculty Mannheim, Heidelberg University, Mannheim, Germany; 12 Department of Psychiatry and Psychotherapy, Medical Center - University of Freiburg, Faculty of Medicine, University of Freiburg, Freiburg, Germany; 13 Health Psychology, School of Social Sciences, University of Mannheim, Mannheim, Germany; 14 SleepWell Research Program, Faculty of Medicine, University of Helsinki, Helsinki, Finland; 15 Department of Psychiatry, University of California San Diego, La Jolla, CA USA; 16 Department of Medical Epidemiology and Biostatistics, Karolinska Institute, Stockholm, Sweden; 17 Institute for Cardiogenetics, University of Lübeck, Lübeck, Germany; 18 DZHK (German Research Centre for Cardiovascular Research), partner site Hamburg/Lübeck/Kiel, Lübeck, Germany; 19 University Heart Center Lübeck, Lübeck, Germany; 20 Social, Genetic and Developmental Psychiatry Centre, Institute of Psychology, Psychiatry and Neuroscience, King’s College London, London, UK; 21 PsychGen Centre for Genetic Epidemiology and Mental Health, Norwegian Institute of Public Health, Oslo, Norway; 22 Department of Child Health and Development, Norwegian Institute of Public Health, Oslo, Norway; 23 PROMENTA Research Center, Department of Psychology, University of Oslo, Oslo, Norway; 24 Department of Quantitative Health Sciences, Division of Computational Biology, Mayo Clinic, Rochester, MN USA; 25 Copenhagen Research Centre for Biological and Precision Psychiatry, Mental Health Centre Copenhagen, Copenhagen University Hospital, Copenhagen, Denmark; 26 Department of Clinical Medicine, Faculty of Health and Medical Sciences, University of Copenhagen, Copenhagen, Denmark; 27 Netherlands Institute for Personality Disorders, Halsteren, Netherlands; 28 Department of Public Health & Novo Nordisk Foundation Center for Protein Research, Faculty of Health and Medical Sciences, University of Copenhagen, Copenhagen, Denmark; 29 Clinical Immunology Research Unit, Department of Clinical Immunology, Odense University Hospital, Odense, Denmark; 30 Department of Clinical Immunology, Zealand University Hospital, Køge, Denmark; 31 Institute of Biological Psychiatry, Mental Health Services, University of Copenhagen, Copenhagen, Denmark; 32 Brain and Mental Health Research Program, QIMR Berghofer, Brisbane, Queensland Australia; 33 School of Psychology, The University of Queensland, Brisbane, Queensland Australia; 34 Psychiatric Genetic Epidemiology Group, Research Department & Nic Waals Institute, Lovisenberg Diaconal Hospital, Oslo, Norway; 35 MRC Integrative Epidemiology Unit, Population Health Sciences, Bristol Medical School, University of Bristol, Bristol, UK; 36 Department of Psychiatry and Psychotherapy, University Medical Center, University of Mainz, Mainz, Germany; 37 Department of Neuroscience, Faculty of Health and Medical Sciences, University of Copenhagen, Copenhagen, Denmark; 38 Department of Clinical Immunology, Copenhagen University Hospital, Rigshospitalet, Copenhagen, Denmark; 39 Department of Clinical Immunology, Aarhus University Hospital, Aarhus, Denmark; 40 Department of Medical Genetics, Oslo University Hospital, Oslo, Norway; 41 Centre for Precision Psychiatry, Division of Mental Health and Addiction, Oslo University Hospital and Institute of Clinical Medicine, University of Oslo, Oslo, Norway; 42 Department of Psychiatry, Sørlandet Hospital, Kristiansand, Agder, Norway; 43 Department of Psychiatry and Psychotherapy, University Hospital Tübingen, Tübingen, Germany; 44 German Center for Mental Health (DZPG), partner site Tübingen, Tübingen, Germany; 45 Department of Psychiatry and Psychotherapy, Christian-Albrechts-Universität zu Kiel, Kiel, Germany; 46 Department of Psychology, Division of Clinical Psychology and Psychotherapy, Saarland University, Saarbrücken, Germany; 47 Institute for Psychopharmacology, Central Institute of Mental Health, Medical Faculty Mannheim, Heidelberg University, Mannheim, Germany; 48 Department of Psychiatry, College of Health Sciences, University of Alberta, Edmonton, Alberta Canada; 49 Neuroscience and Mental Health Institute, University of Alberta, Edmonton, Alberta Canada; 50 Hospital Universitari Institut Pere Mata (HUIPM), Institute de Recerca Biomédica Catalunya Sud (IRB-CatSud) previously IISPV-CERCA, Universitat Rovira i Virgili (URV), Reus, Spain; 51 Biomedical Network Research Centre on Mental Health (CIBERSAM), Instituto de Salud Carlos III, Madrid, Spain; 52 Neurogenomic, Translational Research Centre, Copenhagen University Hospital, Glostrup, Denmark; 53 Danish Headache Center, Copenhagen University Hospital, Rigshospitalet, Copenhagen, Denmark; 54 Landspitali University Hospital, Reykjavik, Iceland; 55 Faculty of Medicine, Department of Psychiatry, School of Health Sciences, University of Iceland, Reykjavik, Iceland; 56 Bradford District Care NHS Foundation Trust, Bradford, UK; 57 Institute of Psychiatric Phenomics and Genomics (IPPG), LMU University Hospital, LMU Munich, Munich, Germany; 58 Human Genomics Research Group, Department of Biomedicine, University of Basel, Basel, Switzerland; 59 Institute of Human Genetics, University Hospital Bonn & University of Bonn, Bonn, Germany; 60 Danish Cancer Institute, Copenhagen, Denmark; 61 Department of Haematology, Copenhagen University Hospital, Rigshospitalet, Copenhagen, Denmark; 62 UK National Institute for Health Research (NIHR) Biomedical Research Centre, South London and Maudsley Hospital and King’s College London, London, UK; 63 National Centre for Register-based Research, Aarhus University, Aarhus, Denmark; 64 Clinic for Child and Adolescent Psychiatry, German Red Cross Hospitals Westend, Berlin, Germany; 65 Institute for Psychology, Department of Clinical Psychology and Psychotherapy, University of Freiburg, Freiburg, Germany; 66 Department of Clinical Immunology, Aalborg University Hospital, Aalborg, Denmark; 67 Psychiatric Center Copenhagen, Copenhagen, Denmark; 68 Institute for Medical and Data Ethics, Heidelberg University, Faculty of Medicine, Heidelberg, Germany; 69 German Cancer Research Center (DKFZ), Heidelberg, Germany; 70 Department of Psychosomatic Medicine and Psychotherapy, Central Institute of Mental Health, Medical Faculty Mannheim, Heidelberg University, Mannheim, Germany; 71 Department of Psychiatry and Neurosciences, Charité – Universitätsmedizin Berlin, corporate member of Freie Universität Berlin and Humboldt- Universität zu Berlin, Campus Benjamin Franklin, Berlin, Germany; 72 Estonian Genome Centre, Institute of Genomics, University of Tartu, Tartu, Estonia; 73 Faculty of Health and Medical Sciences, University of Western Australia, Perth, Western Australia Australia; 74 Department of Clinical Psychology, Central Institute of Mental Health, Medical Faculty Mannheim, Heidelberg University, Mannheim, Germany; 75 Molecular Brain Science, Campbell Family Mental Health Research Institute, Centre for Addiction and Mental Health, Toronto, Ontario Canada; 76 Institute of Medical Science, University of Toronto, Toronto, Ontario Canada; 77 First Department of Psychiatry, Eginition Hospital Medical School, National and Kapodistrian University of Athens, Athens, Greece; 78 Department of Research and Innovation, Division of Clinical Neuroscience, Oslo University Hospital, Oslo, Norway; 79 Institute of Clinical Medicine, Faculty of Medicine, University of Oslo, Oslo, Norway; 80 HUNT Center for Molecular and Clinical Epidemiology, Department of Public Health and Nursing, Faculty of Medicine and Health Sciences, Norwegian University of Science and Technology, Trondheim, Norway; 81 Department of Psychiatry and Psychotherapy, LMU University Hospital Munich, Ludwig Maximilian University Munich, Munich, Germany; 82 Institut de Biologia Evolutiva (UPF-CSIC), Department of Medicine and Life Sciences, Universitat Pompeu Fabra, Parc de Recerca Biomèdica de Barcelona, Barcelona, Spain; 83 deCODE genetics / AMGEN, Reykjavik, Iceland; 84 Institute for Molecular Medicine Finland (FIMM), HiLIFE, University of Helsinki, Helsinki, Finland; 85 Department of Research and Innovation, Division of Mental Health and Addiction, Oslo University Hospital, Oslo, Norway; 86 Institute of Basic Medical Sciences, Faculty of Medicine, University of Oslo, Oslo, Norway; 87 German Center for Mental Health (DZPG), Partner Site Munich-Augsburg, Munich-Augsburg, Germany; 88 South London and Maudsley NHS Foundation Trust, London, UK; 89 Department of Psychiatry and Psychotherapy, University Medical Center Göttingen, Göttingen, Germany; 90 German Center for Neurodegenerative Diseases (DZNE), Göttingen, Germany; 91 Leibniz Institute for Neurobiology, Magdeburg, Germany; 92 Department of Psychiatry and Psychotherapy, Otto von Guericke University, Magdeburg, Germany; 93 Department of Psychiatry and Psychotherapy, Sleep Laboratory, Central Institute of Mental Health, Medical Faculty Mannheim, Heidelberg University, Mannheim, Germany; 94 Department of Clinical Psychology and Psychotherapy, Charlotte Fresenius University, Wiesbaden, Germany; 95 Faculty of Medicine, University of Iceland, Reykjavik, Iceland; 96 Centro de Investigación Biomédica en Red de Salud Mental (CIBERSAM), Institut de Recerca Biomèdica Sant Pau (IIB-Sant Pau), Barcelona, Spain; 97 Department of Psychiatry, Hospital de la Santa Creu i Sant Pau, Barcelona, Spain; 98 Department of Psychiatry and Forensic Medicine & Institute of Neurosciences, Universitat Autònoma de Barcelona, Bellaterra, Spain; 99 Borderline Personality Disorder Clinic, Centre for Addiction and Mental Health, Toronto, Ontario Canada; 100 Department of Psychiatry, University of Toronto, Toronto, Ontario Canada; 101 Finnish Institute for Health and Welfare (THL), Helsinki, Finland; 102 Mental health and suicide, Norwegian Institute of Public Health, Oslo, Norway; 103 Division of Mental Health and Addiction, Oslo University Hospital, Oslo, Norway; 104 Statens Serum Institut, Copenhagen, Denmark; 105 Department of Anaesthesiology and Operative Intensive Care, University Hospital Mannheim, Medical Faculty Mannheim/Heidelberg University, Mannheim, Germany; 106 IVAH, Institut für Verhaltenstherapie-Ausbildung Hamburg, gemeinnützige GmbH, Hamburg, Germany; 107 KG Jebsen Centre for Neurodevelopmental Disorders, University of Oslo, Oslo, Norway; 108 Department of Clinical Psychology, University of Amsterdam, Amsterdam, the Netherlands; 109 Department of Psychiatry & Psychology, Division of Computational Biology, Mayo Clinic, Rochester, MN USA; 110 Department of Psychology, Faculty of Arts and Social Sciences, Simon Fraser University, Vancouver, British Columbia Canada; 111 DBT Centre of Vancouver, Vancouver, British Columbia Canada; 112 Department of Biomedicine, University of Basel, Basel, Switzerland; 113 Medical Genetics, Institute of Medical Genetics and Pathology, University Hospital Basel, Basel, Switzerland; 114 Institute of Neuroscience and Medicine (INM-1), Research Center Juelich, Juelich, Germany; 115 Vanderbilt Genetics Institute, Vanderbilt University Medical Center, Nashville, TN USA; 116 Department of Consultation Psychiatry and Psychosomatics, University Hospital Zurich, Zurich, Switzerland; 117 Department of General Psychiatry, University Hospital Heidelberg, Heidelberg University, Heidelberg, Germany; 118 Borderline Personality Disorder Clinic, General Adult Psychiatry and Health Systems Division, Centre for Addiction and Mental Health, Toronto, Ontario Canada; 119 Oberberg Fachkliniken for Psychiatry, Psychosomatics and Psychotherapy, Bad Tölz, Germany; 120 Insitute of Human Genetics, University Hospital Bonn & University of Bonn, Bonn, Germany; 121 Analytic and Translational Genetics Unit, Department of Medicine, Department of Neurology and Department of Psychiatry, Massachusetts General Hospital, Boston, MA USA; 122 The Stanley Center for Psychiatric Research and Program in Medical and Population Genetics, The Broad Institute of MIT and Harvard, Cambridge, MA USA; 123 Mental Health and Psychiatry Department, Vic Hospital Consortium, Vic, Spain; 124 Department of Psychiatric, University Hospitals of Geneva, Geneva, Switzerland; 125 Psychiatric Genetics Unit, Group of Psychiatry, Mental Health and Addiction, Vall d’Hebron Research Institute (VHIR), Universitat Autònoma de Barcelona, Barcelona, Spain; 126 Department of Psychiatry, Hospital Universitari Vall d’Hebron, Barcelona, Spain; 127 Department of Psychiatry and Forensic Medicine, Universitat Autònoma de Barcelona (UAB), Barcelona, Spain; 128 Institute of Clinical Medicine, University of Oslo, Oslo, Norway; 129 Department of Genetics, Microbiology, and Statistics, Faculty of Biology, Universitat de Barcelona, Barcelona, Spain; 130 Department of Psychiatry, Oberberg Fachkliniken for Psychiatry, Psychosomatics and Psychotherapy, Wendisch Rietz, Germany; 131 Department of Psychiatry, University of Halle, Halle, Germany; 132 Institute for Genomic Medicine, University of California San Diego, La Jolla, CA USA; 133 Douglas Institute, Department of Psychiatry, McGill University, Montreal, Quebec Canada; 134 Department of Neurology, Oslo University Hospital, Oslo, Norway; 135 Medical Faculty, University of Basel, Basel, Switzerland; 136 Analytic and Translational Genetics Unit, Massachusetts General Hospital, Boston, MA USA

**Keywords:** Genetics research, Genome-wide association studies, Psychiatric disorders, Behavioural genetics

## Abstract

Borderline personality disorder (BPD) is a severe mental health condition influenced by environmental risk factors (for example, interpersonal trauma) and genetic factors. We conducted the largest genome-wide association study (GWAS) meta-analysis of BPD so far, with a discovery sample of 12,339 cases and 1,041,717 controls, and a replication study of 685 cases and 107,750 controls (all participants of European ancestry). We identified 11 independent associated genomic loci and 9 risk genes in gene-based analyses. We observed a single-nucleotide polymorphism heritability of 17.3% and derived polygenic scores (PGS) that predicted 4.6% of the phenotypic variance in BPD on the liability scale. BPD showed the strongest positive genetic correlations with GWAS of post-traumatic stress disorder, depression, attention deficit hyperactivity disorder, antisocial behavior, and measures of suicide and self-harm. Phenome-wide analyses in Vanderbilt University Medical Center Biobank and UK Biobank using BPD-PGS confirmed these associations and also identified associations with other medical conditions, including obstructive pulmonary disease and diabetes. These analyses highlight BPD as a polygenic disorder, with the genetic risk showing substantial overlap with psychiatric and physical health conditions.

## Abstract (web summary)

Genome-wide association analyses identify risk variants for borderline personality disorder and find genetic correlations with psychiatric disorders, behavioral traits and somatic diseases.

## Main

BPD is a mental disorder characterized by pervasive instability in emotions, interpersonal relationships and self-image as well as impulsive behavior^1^^,2^ (for symptoms, see Supplementary Note). BPD has a prevalence of 0.92–1.90% in Western countries^3^, with symptom onset typically occurring during adolescence. Women are more frequently diagnosed with BPD than men by a ratio of ~3:1, for which a substantial contribution of diagnostic as well as selection bias has been postulated^4,5^. Individuals with BPD display high rates of self-harm, suicidal ideation and suicide attempts. BPD shows substantial symptom overlap and comorbidity with other mental disorders^5,6^, and comorbidity with neurological and somatic health conditions^6,7^. Although some psychotherapies are effective in treating BPD^8^, no psychopharmacological treatments have been approved by the US Food and Drug Administration specifically for BPD^2,9^.

In addition to environmental risk factors such as early interpersonal trauma^10,11^, genetic factors contribute substantially to disorder risk. Twin and family studies estimate the heritability of BPD to be 46–69%^12,13^ and demonstrate that the genetic risk for BPD is partially shared with other mental disorders but also with continuous traits; for example, the Big Five personality traits^14,15^. However, a systematic assessment of shared genetic risk with a broad range of disorders and traits is missing.

For many mental disorders, GWAS meta-analyses of genetic data from tens or hundreds of thousands of cases and controls have successfully identified hundreds of genetic risk loci^16^. By contrast, genetic research on BPD lags behind^17^. In the only GWAS of BPD conducted so far, encompassing 998 cases and 1,545 controls^18^, no single genome-wide significant variants were identified, but significant genetic correlations (*r*_g_) of BPD were observed with bipolar disorder (BIP), schizophrenia (SCZ) and major depressive disorder^18^. Although these findings indicate the potential of using genetic approaches to investigate BPD, research based on those results is limited by large uncertainties in the estimated effect sizes. Additionally, the extent to which sex-specific genetic effects contribute to the observed sex differences in the prevalence and clinical characteristics of BPD remains unclear.

The main aims of the present study were to identify novel genetic risk loci for BPD to improve understanding of the underlying molecular mechanisms and to systematically assess the shared genetic risk between BPD and a broad range of related traits and disorders.

## Results

### GWAS

**Fig. 1:**
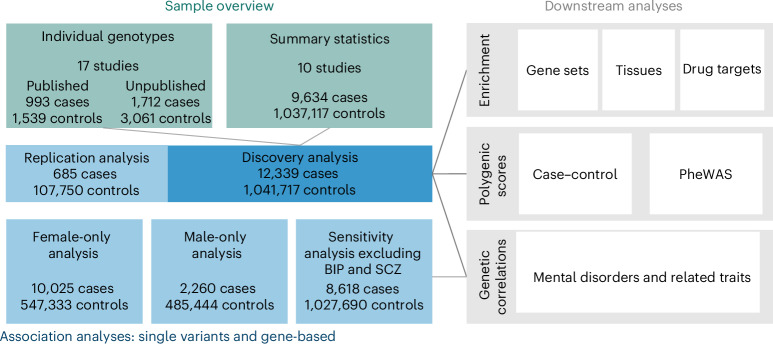
Schematic overview of the GWAS of BPD. A total of 17 studies providing individual-level data (analyzed in five datasets) and ten studies providing summary statistics were included in the discovery meta-analysis. Genetic associations were tested at the single-variant and gene level. Gene enrichment analyses were applied to test for enrichment in gene sets and tissue-specific gene expression data from 54 human tissues (GTEx), comprising 29 different ages and 11 general developmental stages (BrainSpan), single-cell-based cell types and drug-target gene sets. PGS were calculated to assess the prediction of BPD case–control status in the analyzed datasets with available individual-level data and to test the association of the genetic liability for BPD in phenome-wide association studies with phecodes in two biobanks (BioVU, UKB). Genetic correlations were calculated between the results of the GWAS of BPD and the GWAS of 50 disorders and traits of interest.

We performed a discovery GWAS meta-analysis, including 6,043,895 genetic markers in 12,339 BPD cases and 1,041,717 controls for individuals of European ancestry (Fig. 1). In total, 17 studies contributed individual-level data for a total of 2,705 cases meeting the fourth edition of the *Diagnostic and Statistical Manual of Mental Disorders* (DSM-IV) criteria for BPD and 4,600 controls, which were combined into five datasets. Additionally, 10 large-scale biobank or cohort studies provided summary statistics from GWAS of BPD (*n*_cases_ = 9,634; *n*_controls_ = 1,037,117). In these, BPD status was assessed using International Classification of Disease (ICD) codes, except for the Genetic Links to Anxiety and Depression (GLAD) study, which used a self-reported diagnosis. Details on ascertainment, along with inclusion and exclusion criteria, are documented in Supplementary Table 1. Sample sizes, basic demographics (sex and age), depression comorbidity and analysis details are provided in Supplementary Table 2 and Supplementary Note. Additionally, we performed meta-analyses stratified by sex (female only, male only) and a sensitivity analysis excluding individuals with a history of BIP or SCZ—conditions commonly excluded in BPD studies—based on summary statistics available from the studies providing summary statistics (Supplementary Tables 3–5). To assess replication, the lead single-nucleotide polymorphisms (SNPs) and PGS derived from the discovery analysis were tested in two independent datasets (total *n*_cases_ = 685; total *n*_controls_ = 107,750). Additionally, SNPs with *P* < 1 × 10^−6^ in the discovery GWAS were analyzed in a combined meta-analysis (*n*_cases_ = 13,024; *n*_controls_ = 1,149,467). The discovery GWAS had over 80% power to detect effects of 1.1 for variants with an allele frequency between 0.3 and 0.7 (ref. ^19^; Supplementary Table 6 and Supplementary Fig. 1).

We observed a SNP heritability of 28.4% (95% confidence interval (CI), [24.9%, 31.8%]), corresponding to a SNP heritability of 17.3% (95% CI, [15.2%, 19.4%]) on the liability scale (assuming a population prevalence of 1.5%)^3^. The linkage disequilibrium score regression (LDSC)^20^ intercept was 1.03 (s.e. = 0.0085), with an attenuation ratio of 0.11 (s.e. = 0.031), and we observed a genomic inflation factor (*λ*_GC_) of 1.22 (*λ*_1000_ = 1.01). There was a high genetic correlation between the studies with individual-level data and those providing summary statistics (*r*_g_ = 0.85; 95% CI, [0.68, 1.03]; *P* = 3.5 × 10^−21^), which was significantly smaller than 1 (*z* = −1.76; *P* = 0.038).

SNP association analysis revealed six independent genome-wide significant loci (*P* < 5 × 10^−8^; Fig. 2, Table 1, Supplementary Figs. 2–14 and Supplementary Table 7), and no marker showed significant heterogeneity between studies after multiple testing correction (smallest heterogeneity *P* = 0.023). All 6 genome-wide significant lead SNPs (6 out of 6, *P* = 0.016; Table 1), and 77% of the lead SNPs associated with *P* < 1 × 10^−6^ (24 out of 31, *P* = 0.0017) showed effects in the same direction in the replication analysis. In the combined analysis, the 6 loci remained genome-wide significant, and 5 additional loci with *P* < 5 × 10^−8^ were observed (Fig. 2, Table 1 and Supplementary Figs. 2–24).

**Fig. 2:**
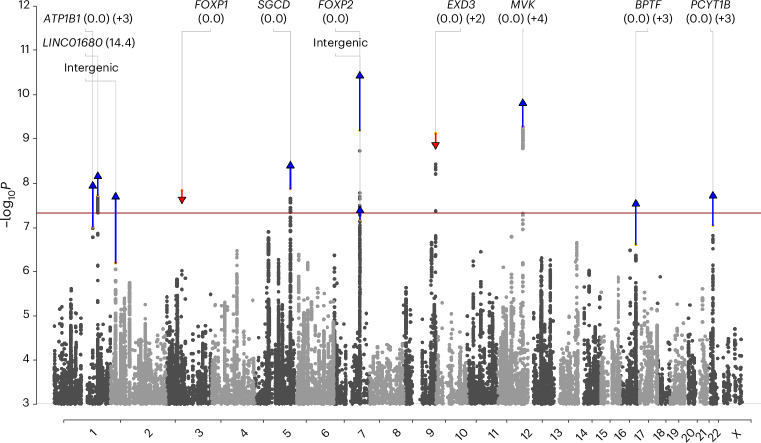
Manhattan plot of the GWAS of BPD. The two-sided −log_10_*P* value for each SNP in the discovery GWAS (inverse-variance-weighted meta-analysis; *n*_cases_ = 12,339; *n*_controls_ = 1,041,717) is indicated on the *y* axis (chromosomal position shown on the *x* axis). The red horizontal line indicates genome-wide significance (*P* < 5 × 10^−8^). Index SNPs representing independent genome-wide significant associations either in the discovery GWAS or the combined analysis (*n*_cases_ = 13,024; *n*_controls_ = 1,149,467) are highlighted (upward-pointing blue triangle, increased significance in combined analysis; downward-pointing red triangle, reduced significance in combined analysis). Combined *P* values are plotted for lead loci, and vertical lines indicate the *P* value change from the discovery to the combined analysis.

**Table 1:** Lead genome-wide significant SNPs associated (*P* < 5 × 10^−8^) with BPD

Locus	CHR	SNP	BP	Locus (start‥stop)	A1/A2	FRQ case	FRQ control	OR	s.e.	*P*	OR repl.	*P* repl.	OR comb.	s.e. comb.	*P* comb.	Mapped genes
Comb. 1	1	rs2143288	169084340	169082360‥169084340	G/A	0.858	0.868	0.89	0.022	1.06 × 10^−7^	0.86	0.035	0.89	0.021	**1.23** **×** **10**^**−8**^	*LINC00970* (0.0), *LOC101928596* (0.0), *ATP1B1* (0.0), *NME7* (0.0)
1	1	rs9970854	191125935	191005935‥191148335	G/A	0.595	0.597	0.92	0.015	**1.99** **×** **10**^**−8**^	0.93	0.183	0.92	0.014	**7.42** **×** **10**^**−9**^	*LINC01680* (14.4)
Comb. 2	2	rs12466671	22583435	22548735‥22606235	A/G	0.587	0.599	0.93	0.015	6.80 × 10^−7^	0.85	0.002	0.92	0.014	**2.16** **×** **10**^**−8**^	*-*
2	3	rs6549383	71266885	71261185‥71267720	T/C	0.503	0.503	1.09	0.015	**1.53** **×** **10**^**−8**^	1.02	0.664	1.08	0.014	**2.45** **×** **10**^**−8**^	*FOXP1* (0.0)
3	5	rs2135029	155856538	155743538‥155868438	A/G	0.594	0.599	0.92	0.014	**1.41** **×** **10**^**−8**^	0.92	0.141	0.92	0.014	**4.30** **×** **10**^**−9**^	***SGCD*** (0.0)
4	7	rs4727799	114110568	114021868‥114224568	T/C	0.663	0.67	1.10	0.015	**6.92** **×** **10**^**−10**^	1.15	0.015	1.10	0.015	**4.05** **×** **10**^**−11**^	***FOXP2*** (0.0)
Comb. 3	7	rs10953781	114974695	114963595‥115112695	C/T	0.528	0.534	1.08	0.014	7.13 × 10^−8^	1.06	0.313	1.08	0.014	**4.38** **×** **10**^**−8**^	*-*
5	9	rs73581580	140251458	140242728‥140278158	G/A	0.836	0.839	0.89	0.019	**8.09** **×** **10**^**−10**^	0.98	0.798	0.89	0.019	**1.45** **×** **10**^**−9**^	*NRARP* (−4.8), ***EXD3*** (0.0), *NOXA1* (16.4)
6	12	rs7304862	109988891	109851891..110050091	G/T	0.529	0.513	1.09	0.014	**5.71** **×** **10**^**−10**^	1.09	0.114	1.09	0.014	**1.68** **×** **10**^**−10**^	*MYO1H* (-52.7), *KCTD10* (−23.5), *UBE3B* (0.0), ***MMAB*** (0.0), ***MVK*** (0.0)
Comb. 4	17	rs7210027	65977053	65825053‥66098053	T/C	0.77	0.782	0.92	0.017	2.54 × 10^−7^	0.87	0.031	0.91	0.016	**3.12** **×** **10**^**−8**^	*BPTF* (0.0), *C17orf58* (0.0), *KPNA2* (4.7), *LINC00674* (70.6)
Comb. 5	X	rs5944622	24613189	24578589‥24933189	T/C	0.57	0.58	0.93	0.013	9.47 × 10^−8^	0.92	0.037	0.93	0.013	**2.05** **×** **10**^**−8**^	*PDK3* (0), *PCYT1B* (0), *PCYT1B*−*AS1* (+5.0), *POLA1* (+48.8)

Results of the variance-weighted meta-analysis are shown (two-sided significance test). Variants are sorted by chromosomal position. CHR, chromosome; Locus (start.stop), start and end of the boundaries of the region within *r*^2^ of 0.6 of the index SNP; A1, allele 1; A2, allele 2; FRQ, frequency of A1 in cases–controls; BP, base pair position; Comb., combined analysis; OR, odds ratio of A1; repl., replication analysis; s.e., standard error. Mapped genes, genes within *r*^2^ of 0.6 and 50 kb of the index SNP (distance to the 50 kb for boundary of the lead SNP in kb is indicated in brackets; genes in bold were also identified by the gene-based association tests). *P* values in bold indicate genome-wide significance (*P* < 5 × 10^−8^). OR, s.e. and *P* values are reported for the main analysis (*n*_cases_ = 12,339; *n*_controls_ = 1,041,717), the replication analysis (*n*_cases_ = 685; total *n*_controls_ = 107,750) and the combined meta-analysis (*n*_cases_ = 13,024; *n*_controls_ = 1,149,467) of the two datasets.

**Extended Data Fig. 1:**
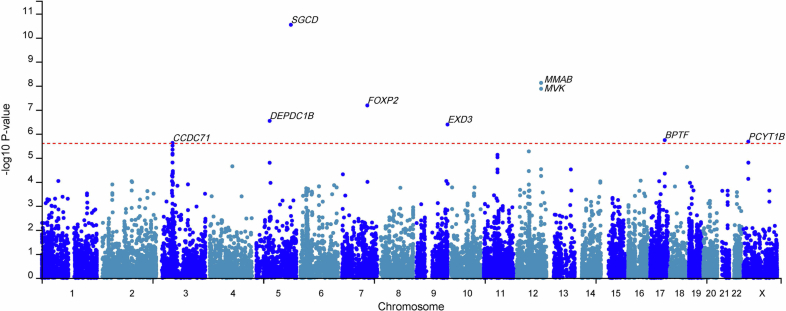
Manhattan plot of the gene-based analysis (*n*_cases_ = 12,339, *n*_controls_ = 1,041,717). The two-sided –log_10_ *P-*value of the MAGMA gene-based test (SNP-wise mean model) for each gene is indicated on the *y*-axis (chromosomal position shown on the *x*-axis). The red line indicates significance after correction for multiple testing (*P* < 0.05/19,843 = 2.5×10^−6^). Gene names are given for significant genes. Source data

Gene-based association analyses using MAGMA (v.1.07)^21,22^ implicated nine genes, including *CCDC71* and *DEPDC1B*, in addition to *SGCD*, *FOXP2*, *EXD3*, *MVK*, *MMAB*, *PCYT1B* and *BPTF* already implicated by the genome-wide SNP associations (Extended Data Fig. 1, Supplementary Fig. 25 and Supplementary Table 8).

Among the genes implicated by proximity to lead SNPs or by the gene-based analysis, the highest polygenic priority scores (PoPS)^23^ were observed for *FOXP2* (score, 0.96; rank, 1) and *SGCD* (score, 0.74; rank, 2), with *FOXP1* and *DEPDC1B* also ranking in the top 1% (Supplementary Table 9). Of the genes in proximity to loci that reached significance in the combined analysis, *NME7* and *KPNA2* ranked highest (top 5%).

### Sex-stratified GWAS

We carried out sex-stratified GWAS meta-analyses for male (*n*_cases_ = 2,260; *n*_controls_ = 485,444) and female study participants (*n*_cases_ = 10,025; *n*_controls_ = 547,333) (Supplementary Tables 3 and 4). We used sex-specific population prevalences of 2.25% for females and 0.75% for males to convert heritability estimates to the liability scale^4,5^. For females, we observed a SNP heritability of 30.3% (95% CI, [26.1%, 34.5%]), corresponding to 20.5% (95% CI, [17.7%, 23.4%]) on the liability scale. For males, the observed SNP heritability was 19.8% (95% CI, [7.9%, 31.6%]), corresponding to 10.2% (95% CI, [4.1%, 16.3%]) on the liability scale, which was significantly lower than in females (*z* = −3.32; *P* = 0.00090). There was a high genetic correlation between the two analyses (*r*_g_ = 0.80; 95% CI, [0.53, 1.07]; *P* = 4.7 × 10^−9^), which did not differ from 1 (*z* = −1.60; *P* = 0.055).

**Extended Data Fig. 2:**
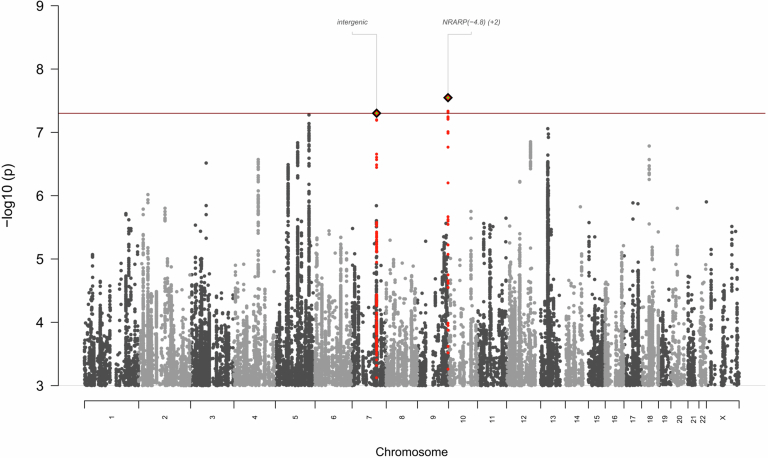
Female subset Manhattan plot of the GWAS meta-analysis (*n*_cases_ = 10,025, *n*_controls_ = 547,333). The two-sided –log_10_ *P-*value for each single-nucleotide polymorphism (SNP) in the inverse variance weighted GWAS meta-analysis is indicated on the *y*-axis (chromosomal position shown on the *x*-axis). The red line indicates genome-wide significance (*P* < 5×10^−8^). Index SNPs representing independent genome-wide significant associations are highlighted as diamonds, and SNPs in linkage disequilibrium with the index SNPs are highlighted in green.

**Extended Data Fig. 3:**
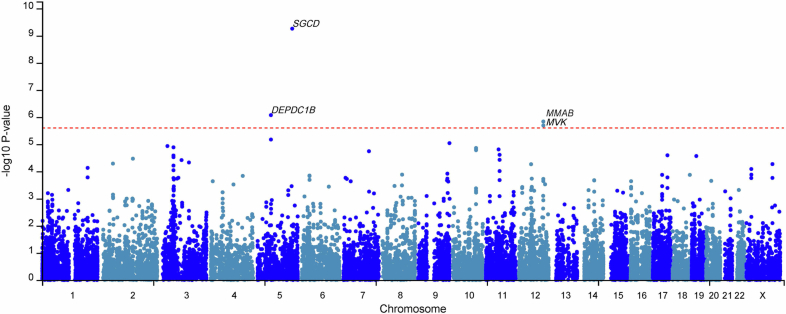
Female subset Manhattan plot of the gene-based analysis (*n*_cases_ = 10,025, *n*_controls_ = 547,333). The two-sided –log_10_ *P-*value of the MAGMA gene-based test (SNP-wise mean model) for each gene is indicated on the *y*-axis (chromosomal position shown on the *x*-axis). The red line indicates significance after correction for multiple testing (*P* < 0.05/19,844 = 2.5×10^−6^). Gene names are given for significant genes. Source data

The female-only analysis identified one genome-wide significant risk locus on chromosome 9 (rs73581580; odds ratio (OR), 0.89; 95% CI, [0.85, 0.92]; *P* = 2.83 × 10^−8^; locus 5 identified in the main analysis (*EXD3*) and a second locus on chromosome 7 (rs10227454; OR, 0.87; 95% CI, [0.83, 0.92]; *P* = 4.99 × 10^−8^), ~1 Mb from locus 4 (*FOXP2*, *r*^2^ = 0.005, *D*′ = 0.25; Extended Data Fig. 2, Supplementary Figs. 26–30 and Supplementary Table 10). The gene-based analysis identified genome-wide associations for *DEPDC1B*, *SGCD*, *MVK* and *MMAB*, all significant in the main analysis (Extended Data Fig. 3 and Supplementary Fig. 31).

**Extended Data Fig. 4:**
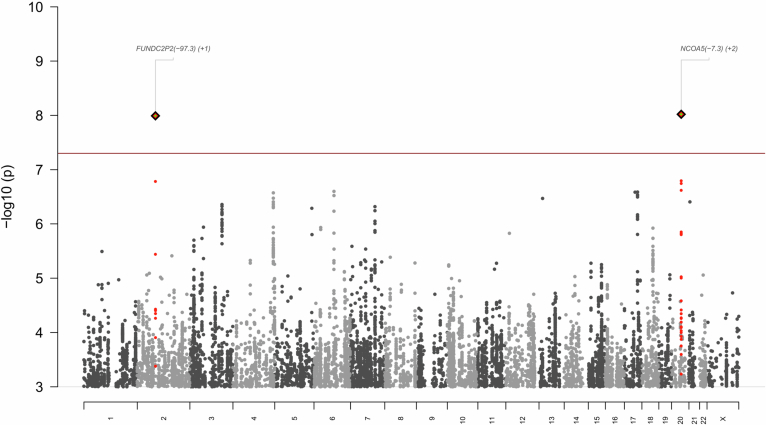
Male subset Manhattan plot of the GWAS meta-analysis (*n*_cases_ = 2,260, *n*_controls_ = 485,444). The two-sided –log_10_ *P-*value for each single-nucleotide polymorphism (SNP) in the inverse variance weighted GWAS meta-analysis is indicated on the *y*-axis (chromosomal position shown on the *x*-axis). The red line indicates genome-wide significance (*P* < 5×10^−8^). Index SNPs representing independent genome-wide significant associations are highlighted as diamonds, and SNPs in linkage disequilibrium with the index SNPs are highlighted in red.

**Extended Data Fig. 5:**
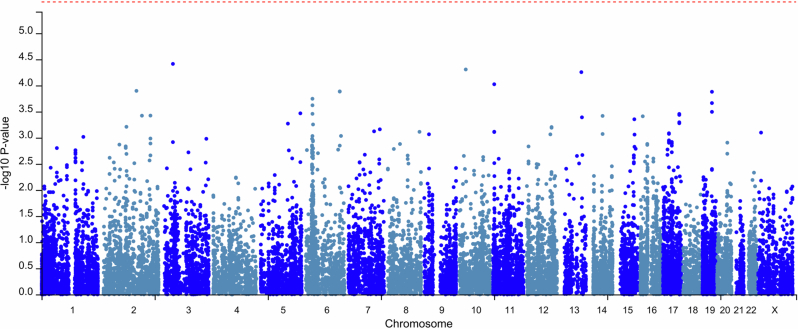
Male subset Manhattan plot of the gene-based analysis (*n*_cases_ = 2,260, *n*_controls_ = 485,444). The two-sided –log_10_ *P-*value of the MAGMA gene-based test (SNP-wise mean model) for each gene is indicated on the *y*-axis (chromosomal position shown on the *x*-axis). The red line indicates significance after correction for multiple testing (*P* < 0.05/19,856 = 2.5×10^−6^). Source data

The male-only analysis identified two genome-wide significant risk loci that were not genome-wide significant in the main analysis: one on chromosome 2 (rs17757829; OR, 0.68; 95% CI, [0.59, 0.77]; *P* = 1.02 × 10^−8^) and one on chromosome 20 (rs6032676; OR, 0.83; 95% CI, [0.77, 0.88]; *P* = 9.55 × 10^−9^) (Extended Data Fig. 4, Supplementary Figs. 32–36 and Supplementary Table 11). The gene-based analysis identified no significant genes (Extended Data Fig. 5 and Supplementary Fig. 37).

**Extended Data Fig. 6:**
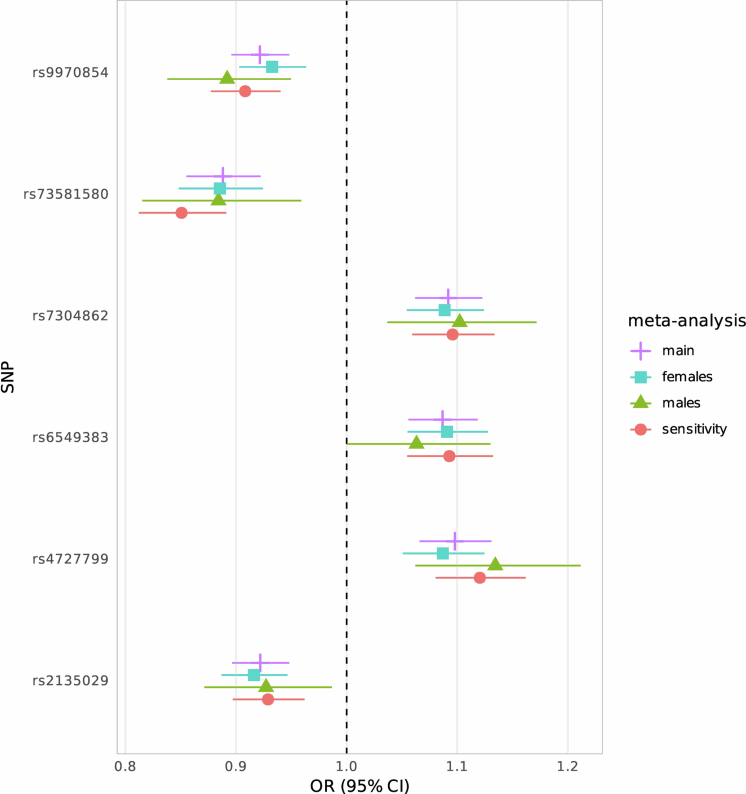
Comparison of the effect sizes of the six GWAS lead SNPs. Odds ratio (OR) estimates from the inverse variance weighted GWAS meta-analyses with error bars indicating 95% confidence intervals are presented for the main meta-analysis, the sex-stratified analyses, and the sensitivity analysis excluding cases with comorbidity of schizophrenia or bipolar disorder from the cohorts providing summary statistics. OR, odds ratio; 95% CI, 95% confidence interval; SNP, single nucleotide polymorphism. Main analysis: *n*_cases_ = 12,339, *n*_controls_ = 1,041,717; female subset: *n*_cases_ = 10,025, *n*_controls_ = 547,333; male subset: *n*_cases_ = 2,260, *n*_controls_ = 485,444; sensitivity analysis: *n*_cases_ = 8,618, *n*_controls_ = 1,027,690. Detailed results can be found in Supplementary Table 12. Source data

The six lead SNPs of the main GWAS showed comparable effects and at least nominal significance in the smaller, and therefore lower-powered, sex-stratified analyses (Extended Data Fig. 6 and Supplementary Table 12). The nine genes significant in the gene-based analysis of the main GWAS all showed nominal significance in the female-only analysis (all *P*_female_ < 8.15 × 10^−5^), whereas *CCDC71*, *DEPDC1B* and *SGCD* were not significant in the male-only analysis (Supplementary Table 13).

### Sensitivity analysis excluding BIP and SCZ

**Extended Data Fig. 7:**
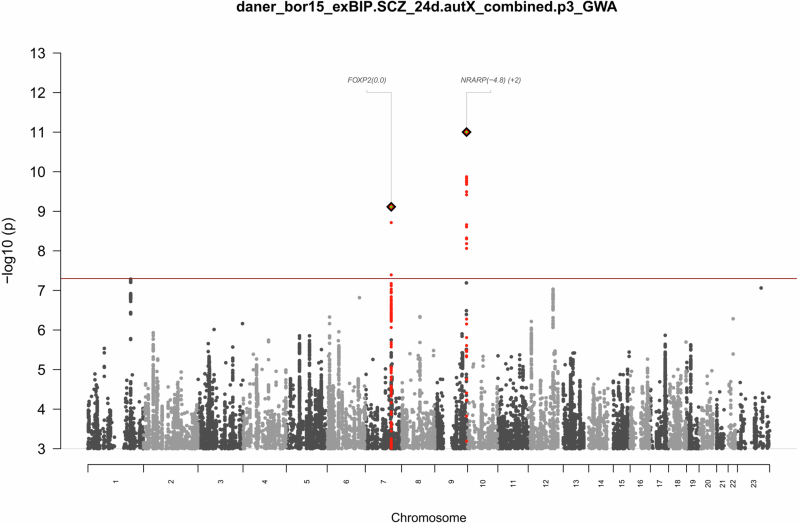
Sensitivity analysis Manhattan plot of the GWAS meta-analysis of BPD excluding individuals with schizophrenia or bipolar disorder (*n*_cases_ = 8,618, *n*_controls_ = 1,027,690). The two-sided –log_10_ *P-*value for each single-nucleotide polymorphism (SNP) in the inverse variance weighted GWAS meta-analysis is indicated on the *y*-axis (chromosomal position shown on the *x*-axis). The red line indicates genome-wide significance (*P* < 5×10^−8^). Index SNPs representing independent genome-wide significant associations are highlighted as diamonds, and SNPs in linkage disequilibrium with the index SNPs are highlighted in red.

**Extended Data Fig. 8:**
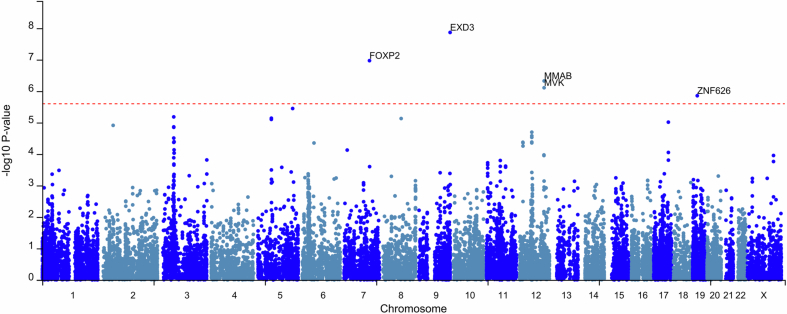
Sensitivity analysis Manhattan plot of the gene-based analysis in the subset excluding individuals with schizophrenia or bipolar disorder (*n*_cases_ = 8,618, *n*_controls_ = 1,027,690). The two-sided –log_10_ *P-*value of the MAGMA gene-based test (SNP-wise mean model) for each gene is indicated on the *y*-axis (chromosomal position shown on the *x*-axis). The red line indicates significance after correction for multiple testing (*P* < 0.05/19,842 = 2.5×10^−6^). Gene names are given for significant genes. Source data

In the sensitivity analysis excluding individuals with BIP or SCZ (*n*_cases_ = 8,618; *n*_controls_ = 1,027,690), locus 4 in *FOXP2* (rs4727799) and locus 5 in *EXD3* (rs73581580) from the main analysis were the only genome-wide significant associations (Extended Data Fig. 7, Supplementary Figs. 38–42 and Supplementary Table 14). All six lead SNPs of the main GWAS showed comparable effects (all *P*_sensitivity_ < 3.32 × 10^−5^; Extended Data Fig. 6 and Supplementary Table 12). Of the nine genes that reached significance in the gene-based analysis of the primary analysis, all remained nominally significant in the sensitivity analysis (*P*_sensitivity_ < 0.0021; Supplementary Table 13), with *FOXP2*, *EXD3*, *MMAB* and *MVK* reaching genome-wide significance, and *ZNF626* being the only additional genome-wide significant gene (Extended Data Fig. 8 and Supplementary Fig. 43).

### Enrichment analysis

Enrichment of gene sets and tissue expression using Genotype-Tissue Expression (GTEx; v.8) data for 54 tissue types^24^ and expression data from 29 different ages and 11 general developmental stages (BrainSpan)^25^ was tested with MAGMA (v.1.07)^21^ as implemented in FUMA^22^. One significant gene set was identified: the S1P–S1P3 (sphingosine-1-phosphate–sphingosine-1-phosphate receptor 3) pathway^26^ (29 genes; standardized effect size *β* = 0.03; *P* = 1.91 × 10^−6^; *P*_adj_ = 0.018; Supplementary Table 15). Nominally significant enrichment was observed in several GTEx tissue types, with the most significant enrichment in cerebellar tissue (not significant after correction for multiple testing; all *P*_adj_ > 0.32; Supplementary Fig. 44 and Supplementary Table 16). The analysis using the two BrainSpan datasets indicated the strongest enrichments 8–21 weeks post conception (not significant after correction; all *P*_adj_ > 0.09) and in early-to-mid prenatal phases, respectively (*P*_adj_ < 0.025; Supplementary Figs. 45 and 46 and Supplementary Tables 17 and 18).

Analyses based on Human Brain Atlas single-nucleus RNA sequencing data^27^ showed SNP-*h*^2^ enrichment using stratified LDSC^28,29^ in 2 out of the 31 tested superclusters (‘medium spiny neurons’: enrichment = 5.99, *z* = 3.58, *P* = 0.00017, *P*_adj_ = 0.0054; and ‘*LAMP5-LHX6* and Chandelier cells’: enrichment = 5.50, *z* = 3.53, *P* = 0.00021, *P*_adj_ = 0.0064; Supplementary Fig. 47 and Supplementary Table 19).

### Drug-target analysis

Of the 16 genes prioritized from the discovery GWAS meta-analysis through proximity to the six GWAS loci or in the gene-based test, none were highlighted as drug targets by Open Targets, although several showed potential tractability (Supplementary Fig. 48). The Genome for REPositioning drugs (GREP) pipeline revealed no significant enrichment for drug targets across any ICD or ATC category. The Drug–Gene Interaction Database (DGIdb) highlighted drug–gene interactions for *MYO1H* with lithium, *MVK* with alendronate sodium and *PCYT1B* with the unapproved substances CT-2584 and sphingosine. In an additional analysis with DRUGSETS^30^, based on MAGMA, testing the enrichment of the GWAS associations in 735 drug–gene sets; the most significant drugs included medications for neurological conditions (safinamide, gabapentin, lomerizine, oxcarbazepine), pain (prilocaine, tetracaine) and alcohol dependence (acamprosate), but also medications for metabolic or somatic conditions (Supplementary Table 20; all *P*_adj_ > 0.18).

### PGS

PGS for BPD were calculated using PRS-CS^31^ in the datasets for which individual-level genotype information was available using leave-one-out summary statistics, excluding the respective sample from the discovery GWAS. In addition, we calculated PGS in the two independent replication datasets (Methods). For comparison, PGS were additionally calculated based on the 2017 BPD GWAS^18^. The explained variance was converted to the liability scale (population prevalence, 1.5%)^3^.

**Fig. 3:**
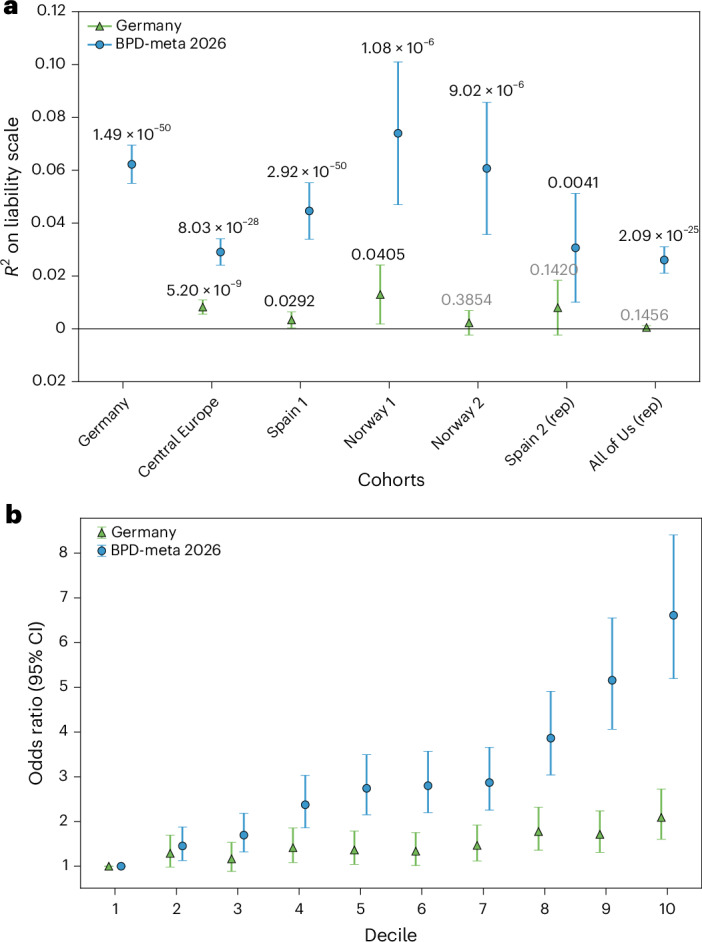
PGS analysis. **a**, The proportion of variance in case–control status explained by the PGS on the liability scale (*y* axis; Liability *R*^2^). **b**, OR for BPD by PGS deciles, with decile 1 as reference. Leave-one-out PGS were calculated for the datasets with available individual-level genotype, including Germany (*n*_cases_ = 993; *n*_controls_ = 1,539), Central Europe (*n*_cases_ = 1,285; *n*_controls_ = 1,332), Spain 1 (*n*_cases_ = 306; *n*_controls_ = 759), Norway 1 (*n*_cases_ = 57; *n*_controls_ = 700) and Norway 2 (*n*_cases_ = 64; *n* = 270 controls), and for two independent replication (rep) datasets: Spain 2 (replication; *n*_cases_ = 46; *n*_controls_ = 435) and All of Us (replication; *n*_cases_ = 639 cases; *n*_controls_ = 107,315) (Supplementary Tables 1 and 2) using PRS-CS. For each prediction, the respective dataset was excluded from the used discovery GWAS meta-analysis (*n*_cases_ = 12,339; *n*_controls_ = 1,041,717), while the full sample size was used for the two replication datasets (blue circles, 2026 meta-GWAS). To visualize the increase in variance explained by the PGS, we also calculated PGS based on the first published BPD GWAS, consisting of the Germany sample (green triangles, 2017 GWAS; *n*_cases_ = 993; *n*_controls_ = 1,539)^18^. In **a**, two-sided *P* values shown above each upper error bar were obtained from logistic regression assessing the associations between PGS and BPD case–control status. Nagelkerke’s pseudo-*R*^2^ was computed by comparing the full model (including PGS and ancestry principal components) with a reduced (covariate-only) model. The explained variance was converted to the liability scale of the population, assuming a lifetime disease risk of 1.5%. Error bars in **a**, s.e.m. of the proportion of variance in liability (Liability *R*^2^) explained by the PGS. The analysis presented in **b** excluded the All of Us dataset, as its individual-level data could not be merged with the other datasets owing to data protection regulations. Error bars in **b**, 95% CI. Source data

PGS explained a weighted average of 4.6% (area under the receiver operator characteristic curve (AUC) = 66.0%) of the phenotypic variance on the liability scale (assuming a lifetime prevalence of 1.5%)^3^ in the five datasets included in the discovery meta-analysis (Fig. 3 and Supplementary Table 21). In the independent replication samples, PGS explained 3.1% of the variance (*P* = 0.0041; AUC = 62.4%) in Spain 2 and 2.6% in All of Us (*P* = 2.09 × 10^−25^; AUC = 61.4%). The OR for BPD case–control status comparing the highest PGS decile to the lowest decile was 6.61 (95% CI, [5.20, 8.41]) for PGS based on the current meta-analysis, compared to OR = 2.09 (95% CI, [1.60, 2.72]) for PGS based on the 2017 GWAS. When comparing to the middle 10% of the distribution, we observed an OR of 2.37 (95% CI, [1.94, 2.89]) for the highest decile for PGS based on the current meta-analysis and an OR of 1.44 (95% CI, [1.05, 1.98]) for PGS based on the 2017 GWAS.

### Genetic correlations

#### Correlations with disorders and traits of interest

**Fig. 4:**
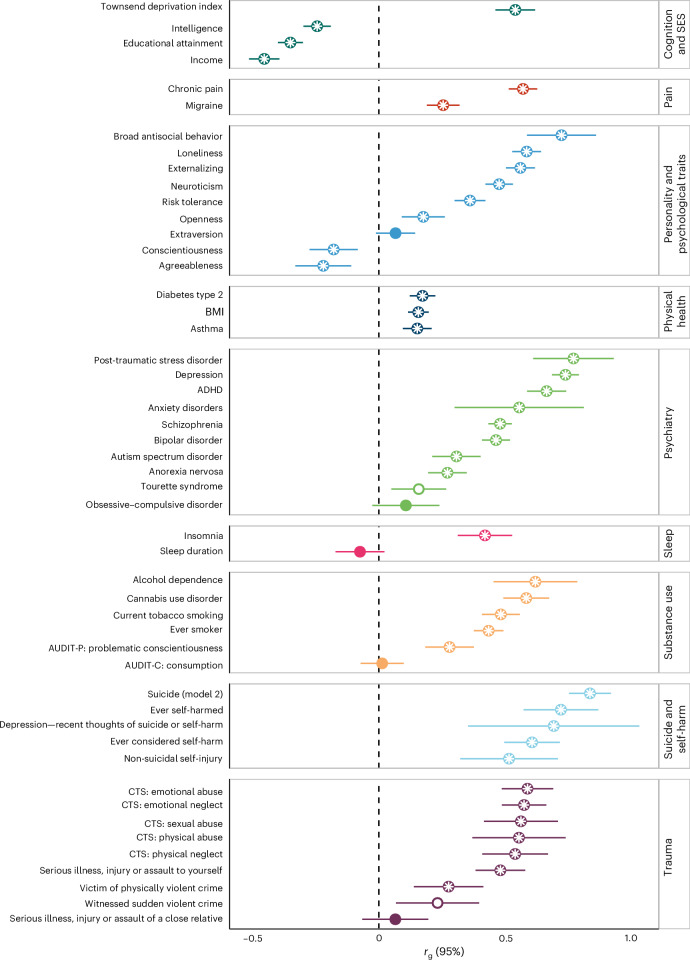
Genetic correlations of BPD with other phenotypes. Genetic correlations of BPD (total *n*_cases_ = 12,339; *n*_controls_ = 1,041,717) with 50 disorders and traits: within each group, disorders and traits are sorted by their genetic correlation. Data are presented as point estimates (genetic correlations) with error bars indicating 95% CIs. Two-sided significance of the genetic correlation is indicated by a white dot (*P* < 0.05) or a white star (*P* < 0.001; 0.05/50 tested correlations). Source and sample size of the used GWAS, as well as the exact *P* values of the tested genetic correlations, are listed in Supplementary Table 22. SES, socioeconomic status. AUDIT, Alcohol Use Disorders Identification Test; BMI, body mass index; CTS, Childhood Trauma Screener. Source data

In a targeted approach, we calculated genetic correlations with a selection of 50 GWAS of other disorders and traits relevant to BPD, including mental disorders, suicide, self-harm, trauma, substance use, physical health, pain, sleep, personality traits (Big Five GWAS including data from 23andMe) and cognition. After Bonferroni correction for multiple testing (*α* = 0.05/50 = 0.001), BPD showed significant genetic correlations with 43 out of the 50 tested phenotypes. Among the psychiatric disorders, BPD showed the strongest correlations with post-traumatic stress disorder (PTSD) (*r*_g_ = 0.77; 95% CI, [0.61–0.93]; *P* = 2.97 × 10^−21^), depression (*r*_g_ = 0.74; 95% CI, [0.69–0.79]; *P* = 6.06 × 10^−163^) and attention deficit hyperactivity disorder (ADHD) (*r*_g_ = 0.67; 95% CI, [0.59–0.75]; *P* = 7.20 × 10^−63^). In the other domains, substantial genetic correlations with |*r*_g_| > 0.5 included material deprivation, chronic pain, broad antisocial behavior, loneliness, externalizing traits, measures of suicide, self-harm and trauma. Lower but significant genetic correlations (*r*_g_ < 0.18) were observed for the somatic disorders type 2 diabetes and asthma, and for body mass index (Fig. 4 and Supplementary Table 22).

The sensitivity meta-analysis excluding individuals with BIP or SCZ showed comparable genetic correlations (Supplementary Fig. 49 and Supplementary Table 23) with the 50 disorders and traits. Notably, lower but still substantial genetic correlations were observed with BIP (*r*_g_ = 0.35; 95% CI, [0.28, 0.42] versus *r*_g_ = 0.47; 95% CI, [0.41–0.53]) and SCZ (*r*_g_ = 0.36; 95% CI, [0.31,0.41] versus *r*_g_ = 0.48; 95% CI, [0.43, 0.53]). The genetic correlation of the main meta-analysis and the sensitivity analysis showed *r*_g_ = 1.00 (95% CI, [0.98, 1.02]; *P* < 1 × 10^−308^).

### Phenome-wide association studies

**Fig. 5:**
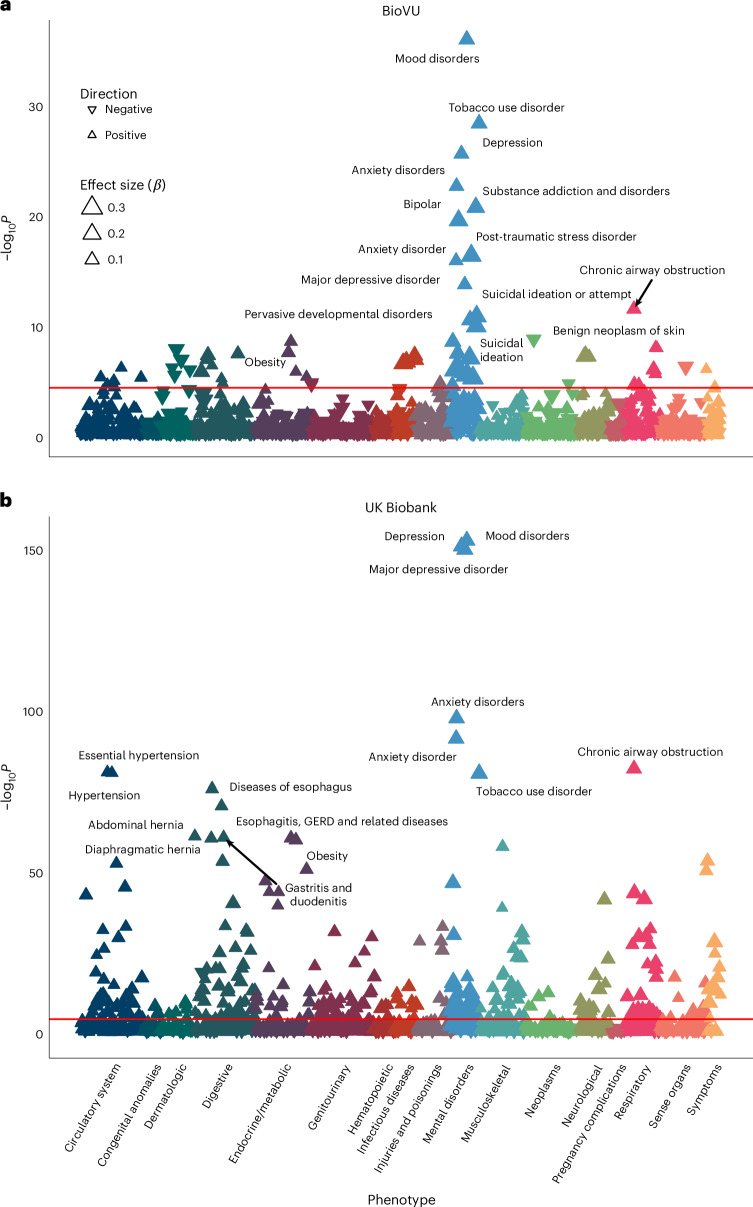
PheWAS of BPD-PGS. **a**,**b**, Association of BPD-PGS with 1,431 tested phecodes in BioVU (**a**) and 1,250 Hospital Episode Statistics phecodes in UKB (**b**). The two-sided statistical significance (−log_10_*P*) from the logistic regression models is plotted on the *y* axis, and diagnoses are grouped by category. The red line indicates the Bonferroni-corrected significance threshold for the tested phecodes (BioVU: *P* < 3.49 × 10^−5^ (0.05/1,431); UKB: *P* < 4.00 × 10^−5^ (0.05/1,250 )). The top 15 associations of each PheWAS are annotated. Upward-pointing triangles indicate positive associations, and downward-pointing triangles indicate negative associations. The size of the triangles indicates the effect size (*β*). Source data

Complementing the targeted genetic correlation analysis, we characterized the genetic signal identified in the BPD GWAS by performing two phenome-wide association studies (PheWAS). To this end, we tested the association of BPD-PGS (using PRS-CS^31^; excluding each target sample from discovery) with ‘phecodes’; that is, medical phenotypes based on ICD diagnoses documented in the electronic health records (EHR), using data from the Vanderbilt University Medical Center Biobank (BioVU)^32^ (66,325 individuals; 214 with the phecode 301.20: ‘antisocial/borderline personality disorder’) and Hospital Episode Statistics in the UK Biobank (UKB) (316,635 individuals; 211 with the phecode 301.20) (Fig. 5).

#### PheWAS in BioVU

**Extended Data Fig. 9:**
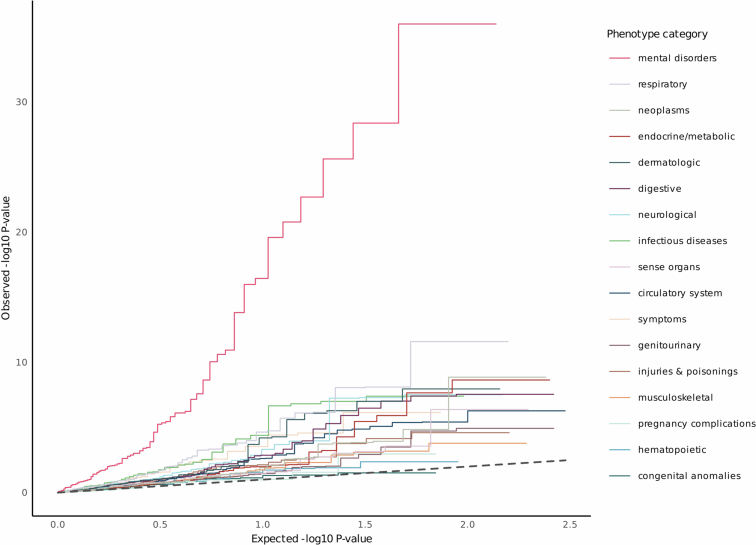
Quantile–quantile plot of the PheWAS analysis in BioVU (*n* = 66,325), stratified by phecode category (1,431 tested phecodes). Observed two-sided –log_10_ *P*-values obtained from the logistic regression analysis are shown on the *y*-axis, and expected –log_10_ *P*-values are shown on the *x*-axis. Detailed results can be found in Supplementary Table 24.

In the PheWAS analysis in BioVU, 69 out of the 1,431 tested diagnoses showed an association with BPD-PGS after Bonferroni correction (*P*_adj_ < 0.05; Supplementary Table 24). BPD-PGS showed the most statistically significant associations with codes from the mental disorders category (Extended Data Fig. 9), including mood disorders, substance use disorders, anxiety, PTSD and suicidal ideation or attempt. Associations were also observed in other categories, including neurological (for example, epilepsy) and somatic disorders (for example, type 2 diabetes, chronic airway obstruction, hypertension) and pain disorders and symptoms.

#### PheWAS in UKB

**Extended Data Fig. 10:**
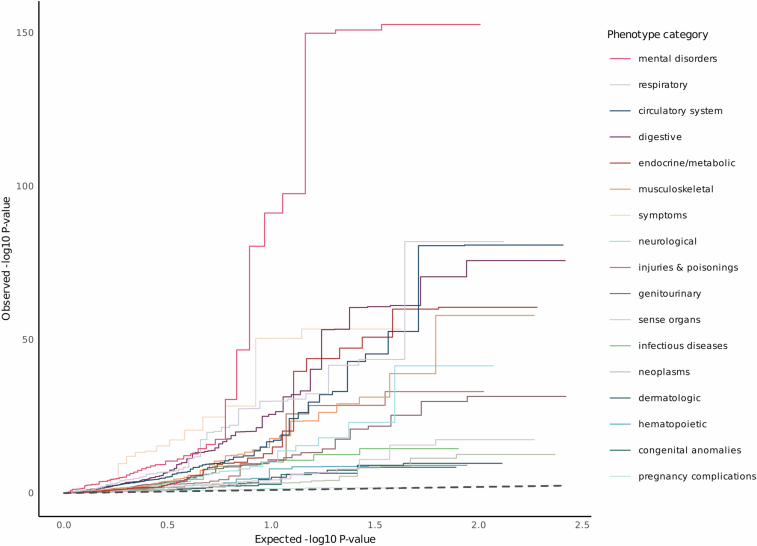
Quantile–quantile plot of the PheWAS analysis in UKB (*n* = 316,635), stratified by phecode category (1,250 tested phecodes). Observed two-sided –log_10_ *P-*values obtained from the logistic regression analysis are shown on the *y*-axis, and expected –log_10_ *P*-values are shown on the *x*-axis. Detailed results can be found in Supplementary Table 25.

In the PheWAS analysis in the UKB, 317 out of the 1,250 tested diagnoses showed an association with BPD-PGS (*P*_adj_ < 0.05; Supplementary Table 25). BPD-PGS showed the most statistically significant associations with codes from the mental disorders category (Extended Data Fig. 10), including mood disorders, tobacco use disorders and anxiety disorders. As in BioVU, significant associations after Bonferroni correction were observed in other categories, including respiratory (for example, chronic airway obstruction), digestive (for example, esophagus, gastroesophageal reflux disease), circulatory, endocrine/metabolic and neurological diagnoses, as well as pain disorders and symptoms.

Of the 69 diagnoses that were significant in the BioVU, 56 were also present with sufficient case numbers in the UKB. Of these, 51 showed effects in the same direction, and 41 also reached significance in the UKB (*P*_adj_ < 0.05). In both samples, the phecode 301.20 (‘antisocial/borderline personality disorder’) showed the strongest effect size (OR_BioVU_ = 1.41; 95% CI, [1.24, 1.63]; *P* = 7.61 × 10^−7^; OR_UKB_ = 1.54; 95% CI, [1.34, 1.76]; *P* = 5.82 × 10^−10^).

## Discussion

The present GWAS, including 12,339 BPD cases and 1,041,717 controls, represents a major advance in BPD genetics, demonstrating substantial SNP heritability, identifying genome-wide significant risk loci, revealing pathway and tissue enrichment and considerably improving the predictive value of derived PGS. To our knowledge, this study represents the first sex-stratified and X-chromosomal GWAS of BPD, as well as the first systematic investigation of shared genetic risk for the disorder. The validity and generalizability of the results is supported by the replication of the lead SNP associations and PGS analyses in independent datasets.

The substantial increase in sample size was achieved by using a strategy combining studies with different ascertainment strategies. Building on our previous study, which included only clinical studies that explicitly recruited patients with BPD^18^, we extended the approach to also include biobank and cohort studies in which diagnoses were primarily derived from electronic health records linked to genetic data. The high genetic correlation indicates that the two subsets capture a largely identical genetic architecture.

A substantial proportion of the heritability observed in family and twin studies (46–69%)^12,13^ was explained by common genetic variation (estimated SNP heritability of 17%). This is consistent with GWAS in other psychiatric disorders, in which SNP heritability often accounts for approximately one-third of the estimates from twin and family studies^16^. The substantial contribution of common genetic variation to BPD is also supported by the leave-one-out PGS analyses, predicting a weighted average of 4.6% of the variance on the liability scale. Importantly, the low LDSC intercept (1.03) and attenuation ratio of 11% suggest that the association is largely driven by the polygenic signal and not by confounding.

In the context of identifying genetic risk variants and genes, the present study is the first to identify genome-wide loci associated with BPD. The genetic risk loci were located in or near genes including *SGCD*, *EXD3*, *FOXP1* and *FOXP2*, and one locus mapped to several genes: *MVK*, *MMAB* and *UBE3B*. *SGCD* encodes sarcoglycan delta, a component of the sarcoglycan complex, and *SGCD* mutations have been associated with muscular and cardiovascular disorders^33^. Common variation in *SGCD* has also been associated with mental disorders, like SCZ^34,35^ and PTSD^36^, substance use phenotypes^37–39^ and measures of quality of life^40^. Converging findings in rodents and humans further suggest that the sarcoglycan complex is expressed in the central nervous system in both neurons and astrocytes^41,42^ and may be involved in GABAergic neurotransmission^41^ and cerebrovascular function^43^.

Among the genes mapped by proximity to the lead SNPs or in the MAGMA gene-based analysis, the highest PoPS scores were observed for *SGCD*, *FOXP2*, *FOXP1* and *DEPDC1B. FOXP1* and *FOXP2* are both members of the Forkhead-box (FOX) transcription factor family that are expressed in the brain and are both associated with speech and language development^44^. *FOXP2* has shown significant associations with externalizing behavior^45,46^ and related traits or disorders such as substance use disorders^47–50^, broad antisocial behavior^51^ and ADHD^52^ in several recent GWAS. Observed associations with childhood maltreatment^53^ and PTSD^36,54,55^ further suggest a trauma-related pathway for BPD, while recent GWAS of the Big Five personality traits showed an association of *FOXP2* with agreeableness^56,57^, conscientiousness^57^ and neuroticism^57^. Of the genes indicated by the combined analysis, *NME7* (NME/NM23 family member 7) and *KPNA2* (karyopherin subunit alpha 2) had the highest PoPS scores. NME7 is a component of the gamma-tubulin ring complex, which has a role in microtubule organization^58^. It has been associated with antidepressant treatment response in a gene expression study^59^ and a GWAS^60^. KPNA2, a key component of the nucleocytoplasmic transport system, has mainly been studied with respect to cancer development, but was recently found showing significant brain GWAS and quantitative trait locus associations in a transcriptomic analysis of major depressive disorder^61^.

We present the first GWAS of BPD to include both sex-stratified and X-chromosome analyses, an initial step towards investigating the sex-specific genetic architecture of the disorder, which is relevant concerning the sex differences in BPD prevalence and presentation. We observed substantially higher SNP heritability in females, which has also been described for PTSD and depression, the two mental disorders showing the strongest genetic correlation with BPD^62^. The lead SNPs of the main GWAS, however, showed the same direction and similar effect sizes in the stratified analyses in both sexes, and the genetic correlation of the sex-stratified GWAS was high and did not differ from 1. However, it must be noted that the sex-stratified analyses had limited statistical power, particularly the male-only analysis, as indicated by a SNP-heritability *z*-score of <4 (ref. ^63^). Nevertheless, we consider the sex-stratified analysis an important first step and provide it as a resource. Sex-stratified analyses in larger samples may help to elucidate sex-specific risk factors of BPD^64^. Notably, we comprehensively analyzed genetic variation on the X chromosome and identified the gene encoding choline-phosphate cytidylyltransferase B (*PCYT1B*) as significantly associated with BPD in the gene-based analysis, as well as an associated SNP in the gene with genome-wide significance in the combined analysis. *PCYT1B* is expressed in the brain^65,66^ but has not previously been reported to be associated with mental health phenotypes. However, many previous GWAS analyses have not reported X chromosome data^64^.

We also used genetic association data to investigate expression enrichment and potential drug repurposing for BPD, offering initial insights. The results suggest the importance of genes expressed in the brain and during early prenatal development. Single-cell analyses revealed significant SNP-heritability enrichment in genes expressed in the clusters ‘medium spiny neurons’ and ‘LAMP5-LHX6 and Chandelier cells’. Both clusters have previously been demonstrated to be enriched in mental health phenotypes in a systematic analysis, namely in SCZ, IQ, educational attainment and neuroticism^29^, with ‘medium spiny neurons’ additionally showing enrichment in major depressive disorder^29^ and the ‘Lamp5-LHX6 and Chandelier cell’ cluster being significantly enriched in the most recent BIP GWAS^67^. Together, these findings suggest that specific neuronal cell types with established relevance for psychiatric disorders may also have a role in BPD.

In addition, our analyses identified potential drug targets. Drug-target analysis of the 16 prioritized genes highlighted *MYO1H*, which has been associated with the response to treatment with lithium, a mood stabilizer also suggested to reduce the risk of suicide attempts^68^. However, it should be noted that the gene was indicated by proximity only in the lithium GWAS^69^. The drug-target analysis also suggested *PCYT1B* as a target of sphingosines. Sphingosine-1-phosphate receptor 3 (S1PR3) regulates various cellular processes when bound to its ligand, sphingosine-1-phosphate (S1P), has been implicated in stress resilience in animal studies and shown to be downregulated in PTSD^70^. Notably, the S1P–S1P3 pathway was the only significant gene set in the present analysis. The genome-wide DRUGSETS analysis indicated drugs with high biological plausibility, including medications for neurological disorders and pain; however, our results were not statistically significant after correction for multiple testing and should be interpreted with caution.

Through genetic correlation and PheWAS analyses, we address a critical gap in the literature by systematically assessing the shared genetic architecture of BPD across a broad spectrum of other disorders and traits. We confirmed the genetic correlations observed in the previous BPD GWAS^18^ with depression, BIP, SCZ^18^, neuroticism, openness to experience^71^ and loneliness^72^. With respect to the Big Five personality dimensions, the results highlight the enhanced power of the present BPD GWAS. We now additionally observe negative genetic correlations with agreeableness and conscientiousness, which is consistent with the profiles of the Big Five personality dimensions observed in cases with BPD^73–75^, as well as with data from twin models^14,76^. This suggests that genetic factors underlying variation in personality traits in the general population contribute to the risk for BPD. Among mental disorders, BPD showed the strongest genetic correlations with PTSD, depression, ADHD and anxiety. Notably, these disorders are frequently observed as preceding or comorbid conditions and share clinical features with BPD^5,7,77^. The associations with suicide and self-harm are consistent with the clinical presentation of BPD^78^, the correlations with trauma phenotypes highlight the role these experiences have in BPD^10^ and the strong correlations of BPD with disorders from the internalizing–externalizing spectrum disorders suggest that BPD risk is influenced by the liability for both dimensions^78,79^.

In both PheWAS samples, and consistent with the genetic correlation results, the BPD-PGS were significantly associated with mood disorders, anxiety disorders, PTSD, substance use disorders and suicidal ideation or suicide attempts. The strongest effect was observed for the phecode ‘antisocial/borderline personality disorder’, supporting the specificity of the GWAS signal for BPD. BPD-PGS were also associated with a range of physical health phenotypes, including chronic airway obstruction, type 2 diabetes, obesity and hypertension, for which an increased risk in BPD cases has been previously described^7^. The relatively small number of BPD cases in the PheWAS target samples makes it probable that these associations are driven by shared genetic risk and not solely by comorbidities of BPD cases in the target samples. These results provide a promising starting point for investigating shared genetic risks between BPD and somatic health, which is particularly relevant given that a substantial portion of the reduced life expectancy in individuals with BPD is attributable to physical health conditions^80^. Further research is warranted to understand the mechanisms through which inter-individual differences in genetic liability for BPD influence the risk for different disorders.

The present analyses cannot disentangle the extent to which comorbidities, overlapping symptoms and potential diagnostic misclassifications might have influenced our results^81^. Although we addressed the potential overlap with BIP and SCZ, more fine-grained analyses are needed. In BPD, comorbidity with other mental disorders is the rule rather than the exception^1,5^; for example, approximately 70% of the investigated BPD patients had a history of depression. Restricting GWAS cases to those without psychiatric comorbidity would reduce the number of available study participants and limit the analysis to a less representative (and drastically smaller) subset. Therefore, we consider it a strength of the present analysis that the biobanks and cohorts providing summary statistics performed the main analysis without excluding cases with psychiatric comorbidity. To assess the impact of this strategy, the sensitivity analysis excluded individuals with either a diagnosis of BIP or SCZ, which are common exclusion criteria in dedicated BPD studies, accounting for 30% of the cases. At the level of the single-variant associations, we observed comparable effect sizes in the sensitivity analysis. We therefore consider it unlikely that BIP or SCZ comorbidity substantially biased the GWAS hits. Comparing genetic correlations between the two iterations of the BPD GWAS and the GWAS of BIP and SCZ, we observe attenuated but still substantial genetic correlations in the sensitivity analysis, suggesting shared genetic risk beyond the co-occurrence of the disorders.

The present study represents substantial progress in the identification of the genetic factors underlying BPD. However, there were several limitations. First, the sample size is relatively small compared with studies involving other mental disorders^16^, and statistical power was limited, especially for variants with lower frequency. The inclusion of additional samples may lead to a substantial increase in the number of loci identified^82,83^. Furthermore, expanding the analyses to non-European ancestries is necessary to improve our understanding of the underlying genetic architecture and to facilitate the generalizability of the results^84,85^. Additionally, in this study, we investigated BPD as a categorical diagnosis, as it is commonly used in the clinical context. However, the diagnosis can be heterogeneous and might differ between cohorts. Future studies should consider the heterogeneity of the disorder and examine clinical BPD at a more fine-grained level; for example, by examining individual symptoms or symptom clusters^86,87^. In addition, functional dimensions such as RDoC^88^ and classification systems such as HiTop^89,90^, as well as borderline personality traits or symptom dimensions^2,17,91^ should be incorporated.

In summary, the present GWAS meta-analysis represents a major step forward towards an understanding of the genetic etiology of BPD. To our knowledge, it is the first GWAS of a personality disorder to identify genome-wide significant risk loci and genes, demonstrating that BPD, like other mental disorders, is a complex polygenic disorder.

## Methods

All study participants gave informed consent, and the studies were approved by the respective ethical committees (for details, see Supplementary Table 1 and Supplementary Note).

### Sample description

An overview of the analyses performed can be found in Fig. 1. We conducted a GWAS meta-analysis, including 1,054,056 participants of European ancestry (*n*_cases_ = 12,339; *n*_controls_ = 1,041,717; *n*_eff_ = 2 × 21,617). In total, 17 studies contributed individual-level data (*n*_cases_ = 2,705; *n*_controls_ = 4,600). This included data from the prior BPD GWAS^18^ and data from an additional 1,712 cases and 3,061 controls (meeting DSM-IV criteria for BPD). Furthermore, we included data from ten large-scale biobank or cohort studies that provided summary statistics from GWAS of BPD (*n*_cases_ = 9,634; *n*_controls_ = 1,037,117). Of those, nine cohorts identified BPD status following ICD codes (ICD-10: F60.3; ICD-9: 3018D/301.83; ICD-8: 3013), while in the GLAD study, a self-reported diagnosis was used.

Details on ascertainment, inclusion and exclusion criteria for cases and controls, along with country of origin for each sample, are documented in Supplementary Table 1. A detailed description of the study design, ascertainment of cases and controls, genotyping arrays used in each study and study-specific quality control and imputation procedures for studies sharing summary statistics is provided in Supplementary Table 2 and Supplementary Note.

### Genotyping, quality control and imputation

Samples providing genotype data at the individual level were grouped into datasets based on array and ancestry, resulting in five datasets (Supplementary Table 2). Quality control and imputation were carried out using the RICOPILI pipeline^92^. A detailed description can be found in Supplementary Note.

### GWAS

For studies providing genotype data at the individual level, GWAS analyses were conducted within each dataset using an additive logistic regression model implemented in PLINK (v.1.9)^93^ on imputed genetic dosage data, with the relevant ancestry principal components (details in Supplementary Note) included as covariates. Details on the association analyses for the cohorts providing summary statistics can be found in Supplementary Table 2 and Supplementary Note. In addition to the main analysis, we performed sex-stratified analyses. Dedicated studies of BPD often exclude individuals with BIP and SCZ. To explore the influence of comorbidity with these severe mental disorders on our results, as a sensitivity analysis, association analyses were performed excluding individuals with a history of BIP or SCZ from the studies, providing summary statistics (sensitivity analysis: *n*_cases_ = 8,618; *n*_controls_ = 1,027,690; Supplementary Tables 3–5). In the case of cohorts in which the control population was not filtered for BIP and SCZ in the main analysis (Copenhagen Hospital Biobank and Danish Blood Donor Study; deCODE; Mayo Clinic Biobank; FinnGen), individuals with BIP or SCZ were also excluded from the controls for the sensitivity analysis.

### Meta-analysis

Meta-analysis of all samples providing either individual-level data or summary statistics was conducted using the inverse-variance-weighted fixed-effects model in METAL^94^ as implemented in RICOPILI^92^ with a genome-wide significance threshold of 5 × 10^−8^. To evaluate consistency of effect sizes, we computed Cochran’s *Q*-statistics and the corresponding heterogeneity *P* values and *I*^2^ statistics, which quantify the percentage of total variation owing to heterogeneity. This procedure excluded SNPs with minor allele frequency of < 1% or an imputation quality score of <0.60. SNPs were translated to the HG19 genomic build (Human build GRCh37) when necessary, and both SNPs and SNP alleles were aligned to the Haplotype Reference Consortium reference genome to ensure standardization for the meta-analysis. To ensure a robust analysis with highly credible SNP sets, we excluded SNPs with highly significant heterogeneity (*P* < 0.001) and SNPs with an effective sample size of less than 85%. X chromosome markers were included in the meta-analysis for all samples except the Norwegian Mother, Father and Child Cohort Study, as those data were not available at the time of analysis. To assess the similarity between the five datasets of studies providing individual-level data and the ten studies providing summary statistics, separate meta-analyses were calculated for the two subsets.

The statistical power of the main analysis to detect genome-wide significant associations (*P* < 5 × 10^−8^) was calculated using the Genetic Power Calculator^19^. For a range of allele frequencies (0.01–0.99) and an effect size of 1.1, we calculated the power of the main analysis (*n*_eff_ = 2 × 21,617; calculated as previously described^95^) and the required sample size for a power over 80% (for details, see Supplementary Table 6 and Supplementary Fig. 1).

SNP heritability, based on the autosomal markers, was estimated using LDSC^20^, both as the observed SNP heritability and as the SNP heritability converted to the liability scale, using *n*_eff_ = 43,234 (2 × 21,617)^95^, a corresponding sample prevalence of 0.5 and assuming a population prevalence of 1.5% (ref. ^3^) for the main analysis. For the sex-stratified GWAS, we used sex-specific population prevalences of 2.25% in females and 0.75% in males for the conversion to the liability scale, corresponding to the previously described female-to-male ratio of 3:1 (refs. ^4,5^). To assess the contribution of confounding to the observed signal, we calculated the attenuation ratio $(\frac{\left({\mathrm{Intercept}}-1\right)}{\left({\mathrm{Mean}}\,{\chi }^{2}-1\right)})$.

To test whether the SNP heritability estimates of the sex-stratified GWAS differed (two-sided test), and to test whether the genetic correlations between GWAS subsets (males versus females; individual-level versus summary cohorts) were smaller than 1 (one-sided test), we used the block jackknife extension of LDSC^20^, which has previously been described^96^.

To assess replication of the observed SNP associations, lead SNPs for the thresholds *P* < 1 × 10^−6^ and *P* < 5 × 10^−8^ were tested for replication in a meta-analysis of two independent datasets (Spain 2 and All of Us; total *n*_cases_ = 685; total *n*_controls_ = 107,750). Additionally, for SNPs with *P* < 1 × 10^−6^ in the discovery GWAS analysis, discovery and replication datasets were analyzed in a combined GWAS meta-analysis, and loci with *P* < 5 × 10^−8^ in the combined analysis are reported.

### Gene-based and gene-set tests

Gene-based, gene-set and tissue-enrichment tests were carried out using MAGMA (v.1.07)^21^ as implemented in FUMA (v.1.5.2)^22^. Gene-based tests were performed using the summary statistics of the GWAS meta-analysis for a total of 19,843 genes, with SNPs assigned to genes based on their physical position. SNPs were included in the analysis using boundaries of 35 kb upstream and 10 kb downstream of the genes. A total of 9,237 gene sets were analyzed (MSigDB v.2023.1Hs; limited to gene sets with at least 20 mapped genes). For both gene-based and gene-set tests, a Bonferroni *P* value threshold, corrected for the number of respective tests, was applied (gene-based, *P* < 2.5 × 10^−6^ (0.05/19,843); gene set, *P* < 5.4 × 10^−6^ (0.05/9,237)).

Additionally, PoPS^23^ were calculated, which prioritize genes at GWAS loci using MAGMA gene-level association tests and over 57,000 gene features such as cell-type-specific gene expression, biological pathways and protein–protein interactions. PoPS scores were available for 17,702 autosomal genes, and PoPS scores and ranks are reported.

Tissue enrichment expression was carried out using GTEx (v.8) data for 54 tissue types^24^, and data from BrainSpan, representing 29 different ages and 11 general developmental stages^25^ as implemented in FUMA (v.1.5.2)^22^. In addition, we used the Human Brain Atlas dataset containing single-nucleus RNA sequencing data from approximately 3.369 million nuclei derived from adult post-mortem brain samples, covering 106 anatomical sections^27^. We used cell types at the level of 31 ‘superclusters’ and retained the existing cell-type nomenclature. We calculated expression proportion values for each cell type (‘gene specificity’, previously described in detail^29,97^), resulting in ~1,300 genes per cell type. We subsequently tested the enrichment of heritability in these gene sets using stratified LDSC^28^, adjusting for the 53 baseline annotations. To adjust for multiple hypothesis testing in the tissue expression analyses, we applied a Bonferroni *P* value threshold adjusted for the respective number of tested tissues.

### Drug-target analysis

For the drug-target analysis, we prioritized genes that either mapped to the genome-wide risk loci (Table 1) or were significant in the gene-based analysis. To identify drug targets among these genes, we extracted data from the Open Targets platform using the GraphSGL API. The information provided by Open Targets is based on the ChEMBL database^98^. Additionally, we performed a tractability analysis (that is, the potential to be modulated by a drug) to assess small molecule binding, the presence of accessible epitopes for antibody-based therapy, relevant data for using Proteolysis Targeting Chimeras (PROTACs) and the presence of compounds in clinical trials with modalities other than small molecules or antibodies. Databases were queried on 24 June 2024. Furthermore, we used the GREP (https://github.com/saorisakaue/GREP) pipeline to test for drug-target enrichment across clinical ATC or ICD categories^99^. Finally, we queried the DGIdb^100^.

In addition to these approaches, focusing on the prioritized 16 genes, we explored potential drug targets with the DRUGSETS approach^30^, which integrates the genome-wide association signal. Using this approach, we tested drug–gene sets, compiled from the Clue Repurposing Hub and the DGIdb, each comprising the genes whose protein products are known to be targeted by or to interact with a given drug. We then conducted a competitive gene-set analysis in MAGMA (v.1.08), conditioning on a background set of all 2,281 drug-target genes, to identify drug–gene sets significantly associated with BPD. To account for multiple testing across the 735 gene sets analyzed, we applied a Bonferroni threshold of *P* < 6.80 × 10^−5^ (0.05/735).

### PGS

We applied PGS to predict case–control status in the datasets for which individual-level genotype information was available. PGS were calculated using PRS-CS, a method that uses Bayesian regression to calculate updated (posterior) effect sizes by applying continuous shrinkage to the initial (prior) effect sizes from the discovery dataset using linkage disequilibrium information^31^. The posterior effect sizes account for the linkage disequilibrium between SNPs using external linkage disequilibrium reference panels constructed from the 1000 Genomes Project Phase 3 European samples. PGS were calculated based on both the present meta-analysis and the prior BPD GWAS from 2017 (consisting of the Germany dataset)^18^ for comparison. Using leave-one-out results of the present meta-analysis (2026 meta-GWAS), whereby the respective target dataset was always left out of the meta-analysis, we calculated PGS for all datasets with individual-level genotype information (Germany, Central Europe, Spain 1, Norway 1 and Norway 2, as well as the two replication cohorts (Spain 2 and All of Us; total *n*_cases_ = 685; total *n*_controls_ = 107,750); Supplementary Tables 1 and 2). Similarly, we used the results of the prior BPD GWAS (2017 GWAS) to calculate PGS for the datasets not included in that analysis (Central Europe, Spain 1, Norway 1, Norway 2, Spain 2, All of Us).

As an effect measure of the association between BPD-PGS and case–control status, the Nagelkerke-pseudo-*R*^2^ (Nk*R*^2^) from logistic regression was calculated, comparing the *R*^2^ of the full model including PGS and covariates (ancestry principal components) as predictors to the reduced (null) model including the covariates only. The resulting Nk*R*^2^ was then converted to the liability scale^101^ of the population, assuming a lifetime disease risk of 1.5% (ref. ^3^). Additionally, the AUC was calculated for each target dataset. Average Nk*R*^2^ and AUC were calculated across the five target datasets and weighted by the effective sample size of the respective target samples.

To calculate the OR of the deciles of the PGS distribution, PRS-CS were normalized to have a mean of zero and unit variance before combining them into two sets of scores for comparison: one based on Witt 2017 (*n*_controls_ = 3,496; *n*_cases_ = 1,758) and the other on BPD-meta 2026 (*n*_controls_ = 5,035; *n*_cases_ = 2,751). This part of the PGS analysis excluded the All of Us dataset, as its individual-level data could not be merged owing to data protection regulations. Using the normalized PRS-CS, we categorized the data into ten deciles through quantile binning, assigning each observation a decile number ranging from one (lowest PRS-CS) to ten (highest PRS-CS). We then created dummy variables by coding observations within each decile as cases; those outside that decile were coded as controls. The dummy variable ranged from deciles two (Q2) to ten (Q10), with decile one (Q1) serving as the reference category.

To assess the association between the deciles based on PRS-CS and the actual case and control status, we conducted logistic regression analyses using the decile-based dummy variables for Q2 to Q10. The ORs for each decile were calculated by exponentiating the coefficients from the logistic regression model, controlling for principal components (1, 2, 3, 4, 5, 6, 8, 10, 12, 14, 15 and 20). For the reference comparison (Q1/Q1), an OR of 1 was assigned, indicating no effect. The ORs for each of the remaining deciles (Q2 to Q10) were computed accordingly: OR_Q1/Q*j*_ = exp(*βj*) for *j* = 2,3,…,10. Additionally, 95% CI for the odds ratios were calculated using the standard errors of the coefficients (CI_lower_ = exp(*βj* − 1.96 × s.e.*j*); CI_upper_ = exp(*βj* + 1.96 × s.e.*j*)). As an additional measure of effect, the highest decile (Q10) was compared to the middle 10% of the distribution as a reference, and OR and CI were calculated as described above.

### Genetic correlations

Genetic correlations between subsets and with other disorders and traits were calculated using LDSC^20^. Calculations were carried out with a free intercept and the 1000 Genomes dataset (EUR) as a reference linkage disequilibrium structure panel^102^.

In a targeted approach, genetic correlations were calculated with 50 GWAS incorporating a range of other disorders and traits relevant to BPD, selected based on reported phenotypic and genetic associations in the literature, theoretical considerations and data availability. Those included GWAS of mental disorders, suicide, self-harm, trauma, substance use, physical health, pain, sleep, personality traits (Big Five GWAS including data from 23andMe) and cognition (Supplementary Table 22). A Bonferroni *P* value threshold, corrected for the number of respective tests, was applied (*P* < 1 × 10^−3^ (0.05/50)).

### PheWAS

The targeted genetic correlation analyses were complemented by PheWAS, systematically exploring associations of the polygenic predisposition for BPD with over 1,000 diagnosis-based phenotypes across the diagnostic spectrum.

### PheWAS in BioVU (EHR data)

Data from the BioVU^32^ were used to test the association of BPD-PGS with medical phenotypes based on two or more ICD diagnoses (‘phecodes’; Phecode Map 1.2) documented in the EHRs. For each phecode, a case was defined as having ≥1 ICD code in the phecode group on ≥2 distinct dates, and a control was defined as having no codes in that phecode group. Individuals with only one instance of a code in that phecode group were excluded from the case–control definition for that phecode. PGS were calculated in 66,325 unrelated individuals of European genetic ancestry (214 individuals with phecode 301.20 (‘antisocial/borderline personality disorder’) based on summary statistics excluding the BioVU samples from the discovery meta-analysis (12,024 cases; 1,038,567 controls). PGS were computed using a continuous shrinkage prior to SNP effect sizes using PRS-CS^31^ and standardized to have a mean of 0 and a standard deviation of 1. Phecodes were tested for association with BPD-PGS when at least 100 cases were available using logistic regression models with sex (defined as sex reported at birth from the EHR), age and the first ten genetic principal components as covariates. A total of 1,431 phecodes were included, and a Bonferroni-corrected threshold of *P* < 3.49 × 10^−5^ (0.05/1,431) was applied.

### PheWAS in UKB

An additional PheWAS analysis was carried out using data from the UKB. PGS for BPD were calculated using PRS-CS based on summary statistics from the discovery meta-analysis, excluding the UKB samples (12,157 cases; 1,039,897 controls). The PheWAS was calculated using phecodes (Phecode Map 1.2) based on diagnoses recorded in Hospital Episode Statistics in a maximum sample of 316,635 individuals (211 individuals with phecode 301.20). For each phecode, a case was defined as having ≥1 ICD code in the phecode group, and a control was defined as having no codes in that phecode group. A total of 1,250 phecodes with at least 100 cases were tested for association with sex, age, batch, the first 40 genetic principal components and assessment center as covariates using a Bonferroni-corrected threshold of *P* < 4.00 × 10^−5^ (0.05/1,250).

### Reporting summary

Further information on research design is available in the Nature Portfolio Reporting Summary linked to this article.

## Online content

Any methods, additional references, Nature Portfolio reporting summaries, source data, extended data, supplementary information, acknowledgements, peer review information; details of author contributions and competing interests; and statements of data and code availability are available at 10.1038/s41588-026-02654-3.

## Supplementary information

Supplementary Information Supplementary Note and Supplementary Figs. 1–49.

Reporting Summary

Peer Review File

Supplementary Tables Supplementary Tables 1–25.

## Source data

Source Data Fig. 3 Statistical source data, PGS analyses.

Source Data Fig. 4 Statistical source data, genetic correlation analyses.

Source Data Fig. 5 Statistical source data, PheWAS analyses.

Source Data Extended Data Fig. 1 Statistical source data, gene-based analyses, main analysis.

Source Data Extended Data Fig. 3 Statistical source data, gene-based analyses, female analysis.

Source Data Extended Data Fig. 5 Statistical source data, gene-based analyses, male analysis.

Source Data Extended Data Fig. 6 Statistical source data, comparison of lead SNP effects.

Source Data Extended Data Fig. 8 Statistical source data, gene-based analyses, sensitivity analysis.

## Data Availability

Individual-level data are not publicly available owing to ethical restrictions. The results of the meta-analysis and PGS weights are publicly available through the website of the Psychiatric Genomics Consortium (https://pgc.unc.edu/for-researchers/download-results). GWAS summary statistics of other traits and disorders used for analyses in this study are publicly available (sources listed in Supplementary Table 22). The exceptions are the GWAS summary statistics for self-harm^103^, which were provided by the respective corresponding authors, and the GWAS used for the Big Five personality traits (except neuroticism), which include data from 23andMe and can be made available to qualified investigators if they enter into an agreement with 23andMe that protects participant confidentiality. This study used data from the All of Us Research Program’s Controlled Tier Dataset v7, available to authorized users on the Researcher Workbench. Source data are provided with this paper.

## Code availability

No custom code was developed for the present analyses. GWAS in datasets with individual data were carried out within each dataset using PLINK (v.1.9; http://pngu.mgh.harvard.edu/purcell/plink). Quality control and imputation were carried out using RICOPILI (https://sites.google.com/a/broadinstitute.org/ricopili; https://github.com/Ripkelab/ricopili). Prephasing and imputation was carried out using EAGLE (v.2.4.1; https://alkesgroup.broadinstitute.org/Eagle) and MINIMAC3 (http://genome.sph.umich.edu/wiki/minimac3) using the Haplotype Reference Consortium reference panel (Release 1.1, dataset EGAD00001002729). Meta-analyses of GWAS were performed using METAL (http://www.sph.umich.edu/csg/abecasis/metal). Genetic correlations and heritability estimates were calculated using LDSC (https://github.com/bulik/ldsc). FUMA (v.1.5.2) was used for downstream analyses, including gene-based and gene-set analyses (https://fuma.ctglab.nl), based on MAGMA (v.1.07; http://ctglab.nl/software/magma). Functional annotation within FUMA used data from MSigDB v2023.1Hs, GTEx (v.8; https://fuma.ctglab.nl/downloadPage) and BrainSpan (https://www.brainspan.org). Additionally, genes were prioritized using PoPS (v.0.2; https://github.com/FinucaneLab/pops). Enrichment for drug targets was carried out using GREP (https://github.com/saorisakaue/GREP) and DRUGSETS (https://github.com/nybell/drugsets). Weights for polygenic scores were generated using PRS-CS (v.1.1.0; https://github.com/getian107/PRScs).
