# Supplementary material for: Genome-wide association analyses of borderline personality disorder identify 11 loci and highlight shared risk with mental and somatic disorders
Source: Nat Genet. 2026 Jul 20;58(8):1831–44. doi: 10.1038/s41588-026-02654-3 (PMC13447106; doi:10.1038/s41588-026-02654-3)
Supplement: Supplementary file 2 [file 41588_2026_2654_MOESM2_ESM.pdf]

nature portfolio

Corresponding author(s): Fabian Streit

Last updated by author(s): Dec 1, 2025

# Reporting Summary

Nature Portfolio wishes to improve the reproducibility of the work that we publish. This form provides structure for consistency and transparency in reporting. For further information on Nature Portfolio policies, see our Editorial Policies and the Editorial Policy Checklist.

## Statistics

For all statistical analyses, confirm that the following items are present in the figure legend, table legend, main text, or Methods section.

| n/a | Confirmed | |
|---|---|---|
| ☐ | ☒ | The exact sample size (*n*) for each experimental group/condition, given as a discrete number and unit of measurement |
| ☐ | ☒ | A statement on whether measurements were taken from distinct samples or whether the same sample was measured repeatedly |
| ☐ | ☒ | The statistical test(s) used AND whether they are one- or two-sided<br>*Only common tests should be described solely by name; describe more complex techniques in the Methods section.* |
| ☐ | ☒ | A description of all covariates tested |
| ☐ | ☒ | A description of any assumptions or corrections, such as tests of normality and adjustment for multiple comparisons |
| ☐ | ☒ | A full description of the statistical parameters including central tendency (e.g. means) or other basic estimates (e.g. regression coefficient) AND variation (e.g. standard deviation) or associated estimates of uncertainty (e.g. confidence intervals) |
| ☐ | ☒ | For null hypothesis testing, the test statistic (e.g. *F*, *t*, *r*) with confidence intervals, effect sizes, degrees of freedom and *P* value noted<br>*Give P values as exact values whenever suitable.* |
| ☒ | ☐ | For Bayesian analysis, information on the choice of priors and Markov chain Monte Carlo settings |
| ☒ | ☐ | For hierarchical and complex designs, identification of the appropriate level for tests and full reporting of outcomes |
| ☐ | ☒ | Estimates of effect sizes (e.g. Cohen's *d*, Pearson's *r*), indicating how they were calculated |

*Our web collection on statistics for biologists contains articles on many of the points above.*

## Software and code

Policy information about availability of computer code

| | |
|---|---|
| Data collection | Data collection was not part of this study. |
| Data analysis | RICOPILI: https://sites.google.com/a/broadinstitute.org/ricopili/; https://github.com/Ripkelab/ricopili<br>PLINK 1.9: http://pngu.mgh.harvard.edu/purcell/plink/<br>EAGLE 2.4.1: https://alkesgroup.broadinstitute.org/Eagle/<br>MINIMAC 3: http://genome.sph.umich.edu/wiki/minimac3<br>METAL: http://www.sph.umich.edu/csg/abecasis/metal/<br>MAGMA 1.07: http://ctglab.nl/software/magma<br>FUMA 1.5.2: https://fuma.ctglab.nl/<br>Polygenic Priority Score (PoPS) v0.2: https://github.com/FinucaneLab/pops<br>GREP: https://github.com/saorisakaue/GREP<br>PRS-CS v.1.1.0: https://github.com/getian107/PRScs<br>LDSC: https://github.com/bulik/ldsc<br>DRUGSETS: https://github.com/nybell/drugsets |

For manuscripts utilizing custom algorithms or software that are central to the research but not yet described in published literature, software must be made available to editors and reviewers. We strongly encourage code deposition in a community repository (e.g. GitHub). See the Nature Portfolio guidelines for submitting code & software for further information.

## Data

Policy information about availability of data

All manuscripts must include a data availability statement. This statement should provide the following information, where applicable:

- Accession codes, unique identifiers, or web links for publicly available datasets
- A description of any restrictions on data availability
- For clinical datasets or third party data, please ensure that the statement adheres to our policy

Individual-level data are not publicly available due to ethical restrictions. The results of the meta-analysis and PGS weights are publicly available via the website of the Psychiatric Genomics Consortium (PGC) https://pgc.unc.edu/for-researchers/download-results/.
GWAS summary statistics of other traits and disorders used for analyses in this study are publicly available (sources listed in Supplementary Table 22). The exceptions are the GWAS summary statistics for self-harm104 which were provided by the respective corresponding authors and the GWAS used for the Big Five personality traits (except Neuroticism) which include data from 23andMe, and can be made available to qualified investigators, if they enter into an agreement with 23andMe that protects participant confidentiality. This study used data from the All of Us Research Program's Controlled Tier Dataset v7, available to authorized users on the Researcher Workbench.

## Research involving human participants, their data, or biological material

Policy information about studies with human participants or human data. See also policy information about sex, gender (identity/presentation), and sexual orientation and race, ethnicity and racism.

| | |
|---|---|
| Reporting on sex and gender | Biological sex was used in the study, and analyses stratified by sex are reported additionally. |
| Reporting on race, ethnicity, or other socially relevant groupings | All studies consisted of subjects of European ancestry. The analysis was controlled for effects of population stratification by a) excluding outliers based on ancestry principal components, and b) adjusting for ancestry principal components in the association analyses. |
| Population characteristics | Additional meta-analyses were calculated stratified by sex (female only, male only), and as a sensitivity analysis excluding subjects with comorbidity of schizophrenia or bipolar disorder. The number of female and male subjects in each analysis are listed in the Supplementary Tables S2-S5. Further details on the included populations can be found in Supplementary Table S1 and the Supplementary Note |
| Recruitment | Detailed descriptions of recruitment of all included 28 studies can be found in the Supplementary Table 1 ("study overview") and supplementary methods ("description of studies"). |
| Ethics oversight | All subjects gave informed consent, and the studies were approved by the respective ethical committees (details see Supplementary Table 1 and Supplementary Note). |

Note that full information on the approval of the study protocol must also be provided in the manuscript.

# Field-specific reporting

Please select the one below that is the best fit for your research. If you are not sure, read the appropriate sections before making your selection.

☒ Life sciences ☐ Behavioural & social sciences ☐ Ecological, evolutionary & environmental sciences

For a reference copy of the document with all sections, see nature.com/documents/nr-reporting-summary-flat.pdf

# Life sciences study design

All studies must disclose on these points even when the disclosure is negative.

| | |
|---|---|
| Sample size | Total sample size was defined as the sum of all participants included in each meta-analysis. To account for the disparity of cases and control groups, the effective sample size is additionally reported for each meta-analysis. To maximize power to detect genetic loci, we included all cohorts for which eligible data were available and collaboration was feasible; no a priori sample size calculation was performed. A post-hoc power analysis demonstrated >80% power to detect common variant effects (MAF 0.3-0.7) at genome-wide significance, indicating that the achieved sample size was sufficient for the study aims. |
| Data exclusions | We applied filtering criteria to samples and genetic markers (samples and SNPs with genotype call rate < 98%; difference in SNP genotyping call rate between cases and controls >2%, deviation of autosomal heterozygosity from the mean (\|Fhet\|>0.2), deviation from Hardy-Weinberg equilibrium (HWE; p<1×10−10 in cases; p<1×10−6 in controls); minor allele frequency <1% (5% for X chromosome); INFO score < 0.6; significant heterogeneity (p <0.001), effective sample size <85%). All analyses using individual-level genotype data were adjusted for ancestry principal components (GWAS, PGS analyses, PheWAS). In addition, PheWAS analyses and studies that provided summary statistics adjusted the analyses in their data sets for additional covariates where appropriate including age, sex, and technical or study-specific covariates (e.g., genotyping array, study center). |
| Replication | To maximize discovery, most available studies were included in the discovery analysis (12,339 cases and 1,041,717 controls). Two independent replication cohorts (n total: 685 cases and 107,750 controls) were used to replicate the lead loci, and polygenic risk score |

prediction. All six genome-wide significant lead SNPs (6/6), and 77% of the lead SNPs associated with p<1x10-6 (24/31) showed effects in the same direction in the replication analysis. In the independent replication samples, PGS were associated with case-control status and explained 3.1% of the variance (p=0.0041, AUC=62.4%) in Spain 2, and 2.6% in All of Us (p=2.09x10-25, AUC=61.4%).

Randomization — There was no randomization in the used GWAS design.

Blinding — There was no blinding in the used (observational) design.

# Reporting for specific materials, systems and methods

We require information from authors about some types of materials, experimental systems and methods used in many studies. Here, indicate whether each material, system or method listed is relevant to your study. If you are not sure if a list item applies to your research, read the appropriate section before selecting a response.

## Materials & experimental systems

| n/a | Involved in the study |
|---|---|
| ☒ | ☐ Antibodies |
| ☒ | ☐ Eukaryotic cell lines |
| ☒ | ☐ Palaeontology and archaeology |
| ☒ | ☐ Animals and other organisms |
| ☒ | ☐ Clinical data |
| ☒ | ☐ Dual use research of concern |
| ☒ | ☐ Plants |

## Methods

| n/a | Involved in the study |
|---|---|
| ☒ | ☐ ChIP-seq |
| ☒ | ☐ Flow cytometry |
| ☒ | ☐ MRI-based neuroimaging |

## Plants

Seed stocks — *Report on the source of all seed stocks or other plant material used. If applicable, state the seed stock centre and catalogue number. If plant specimens were collected from the field, describe the collection location, date and sampling procedures.*

Novel plant genotypes — *Describe the methods by which all novel plant genotypes were produced. This includes those generated by transgenic approaches, gene editing, chemical/radiation-based mutagenesis and hybridization. For transgenic lines, describe the transformation method, the number of independent lines analyzed and the generation upon which experiments were performed. For gene-edited lines, describe the editor used, the endogenous sequence targeted for editing, the targeting guide RNA sequence (if applicable) and how the editor was applied.*

Authentication — *Describe any authentication procedures for each seed stock used or novel genotype generated. Describe any experiments used to assess the effect of a mutation and, where applicable, how potential secondary effects (e.g. second site T-DNA insertions, mosiacism, off-target gene editing) were examined.*
