# Supplementary material for: Genome-wide association analyses of borderline personality disorder identify 11 loci and highlight shared risk with mental and somatic disorders
Source: Nat Genet. 2026 Jul 20;58(8):1831–44. doi: 10.1038/s41588-026-02654-3 (PMC13447106; doi:10.1038/s41588-026-02654-3)
Supplement: Supplementary file 3 [file 41588_2026_2654_MOESM3_ESM.pdf]

nature portfolio

Peer Review File

Genome-wide association analyses of borderline personality disorder identify 11 loci and highlight shared risk with mental and somatic disorders

Corresponding Author: Dr Fabian Streit

Version 0:

Decision Letter:

10th January 2025

Dear Dr. Streit,

Your Article "Genome-wide association study of borderline personality disorder identifies six loci and highlights shared risk with mental and somatic disorders" has been seen by three referees. You will see from their comments below that, while they find your work of interest, they have raised several relevant points. We are interested in the possibility of publishing your study in Nature Genetics, but we would like to consider your response to these points in the form of a revised manuscript before we make a final decision on publication.

To guide the scope of the revisions, the editors discuss the referee reports in detail within the team, including with the chief editor, with a view to identifying key priorities that should be addressed in revision, and sometimes overruling referee requests that are deemed beyond the scope of the current study. In this case, we ask that you address all technical queries related to the association analyses and their interpretation, revising the presentation where appropriate and extending the analyses where feasible as requested by the referees. We hope you will find this prioritized set of referee points to be useful when revising your study. Please do not hesitate to get in touch if you would like to discuss these issues further.

We therefore invite you to revise your manuscript taking into account all reviewer and editor comments. Please highlight all changes in the manuscript text file. At this stage, we will need you to upload a copy of the manuscript in MS Word .docx or similar editable format.

We are committed to providing a fair and constructive peer-review process. Do not hesitate to contact us if there are specific requests from the reviewers that you believe are technically impossible or unlikely to yield a meaningful outcome.

When revising your manuscript:

*1) Include a “Response to referees” document detailing, point-by-point, how you addressed each referee comment. If no action was taken to address a point, you must provide a compelling argument. This response will be sent back to the referees along with the revised manuscript.

*2) If you have not done so already, please begin to revise your manuscript so that it conforms to our Article format instructions, available
here.
Refer also to any guidelines provided in this letter.

*3) Include a revised version of any required Reporting Summary: https://www.nature.com/documents/nr-reporting-summary.pdf
It will be available to referees (and, potentially, statisticians) to aid in their evaluation if the manuscript goes back for peer review.
A revised checklist is essential for re-review of the paper.

Please be aware of our guidelines on digital image standards.

EXTENDED DATA FIGURES

When re-submitting your manuscript, please ensure that any supplementary figures and tables that are crucial to the manuscript's conclusions are converted into Extended Data figures and tables to increase visibility of these data. Extended Data figures and tables are online-only (present in the online PDF and full-text HTML versions of the paper), peer-reviewed display items that provide essential background to the article but are not included in the main article due to space constraints. A maximum of ten Extended Data display items (figures and tables) is permitted.

Please use the link below to submit your revised manuscript and related files:

Link Redacted

Note: This URL links to your confidential home page and associated information about manuscripts you may have submitted, or that you are reviewing for us. If you wish to forward this email to co-authors, please delete the link to your homepage.

We hope to receive your revised manuscript within 8-12 weeks. If you cannot send it within this time, please let us know.

Please do not hesitate to contact me if you have any questions or would like to discuss these revisions further.

Nature Genetics is committed to improving transparency in authorship. As part of our efforts in this direction, we are now requesting that all authors identified as 'corresponding author' on published papers create and link their Open Researcher and Contributor Identifier (ORCID) with their account on the Manuscript Tracking System (MTS), prior to acceptance. ORCID helps the scientific community achieve unambiguous attribution of all scholarly contributions. You can create and link your ORCID from the home page of the MTS by clicking on 'Modify my Springer Nature account'. For more information, please visit www.springernature.com/orcid.

We look forward to seeing the revised manuscript and thank you for the opportunity to review your work.

Sincerely,
Kyle

Kyle Vogan, PhD
Senior Editor
Nature Genetics
https://orcid.org/0000-0001-9565-9665

Referee expertise:

Referee #1: Genetics, psychiatric disorders

Referee #2: Genetics, psychiatric disorders

Referee #3: Genetics, psychiatric disorders

Reviewers' Comments:

Reviewer #1 (Remarks to the Author):

Streit et al report the largest GWAS of borderline personality disorder (BPD) to date, including 12,339 cases and 1,041,717 controls of European ancestry. They find 6 GWS loci, SNP-h2 of 17.3% and PGS explaining ~4.6% of phenotypic variance. I appreciated that the X chromosome was included in the GWAS and that sex-specific GWAS were also presented. The study used genetic correlation analyses and PheWAS in two cohorts to identify phenotypes with shared genetic etiology with BPD, which highlighted other psychiatric disorders, suicide and self-harm related traits, and medical conditions including obstructive pulmonary disease and diabetes. Overall, this is a high quality GWAS which makes a meaningful contribution to the literature on the etiology of BPD. I have two main comments:

• The study would be enhanced by adding a single-cell gene expression enrichment analysis to gain insight into the brain cell-types most relevant for BPD. Similarly, an enrichment analysis of genes expressed in the brain at different developmental timepoints would be of interest.

• The drug-target analyses were performed by investigating 16 genes (implicated in GWS loci or gene-based tests) for drug tractability or being existing drug targets using online databases. It would be valuable to test enrichment across the entire genome in the targets of existing medications or classes of medications here as well. When focusing on 16 genes, the search space is very small, whereas the polygenic signal from this GWAS is quite robust.

I also had the following specific comments:

Introduction
The characteristics of BPD were described but including the specific diagnostic criteria and further information on the psychiatric and general medical comorbidities would be helpful. For example, it wasn't clear to me why the sensitivity analyses excluding BIP and SCZ were performed but some additional background information would set this up (e.g., are these the disorders most commonly comorbid with BPD, is misdiagnoses between BPD and BIP/ SCZ common, or was this to match the phenotyping of the biobanks with the clinical samples, which typically exclude BIP/SCZ as noted in the discussion?).

Results
What was the se on the intercept and the LD Score Regression attenuation ratio?

Where genetic correlations are reported between cohorts with individual-level data and summary statistics, or between GWAS in males and females, I recommend adding a test for whether the genetic correlation is significantly different from 1.

Figure 2 – what is the difference between the loci in red and orange?

Table 1 – which allele do the test statistics correspond to?

What were the SNP-h2 estimates of BPD in males and females and were they significantly different from each other?

In the PGS section of the results, it was not clear whether the R2 values in the text were on the liability scale and the values in Figure 3A are presented as Nagelkerke's R2. Please present all results on the liability scale.

In the comparison of the OR for the top versus lowest decile, adding the CIs to the text would be helpful. I would also like to see a comparison of the top versus middle decile, since the individuals in the lowest decile are just as much outliers in the population as those in the top decile.

Figure 3A could be improved by using a color gradient for P value rather than the stars (a different shape could be used for the 2017 and 2024 GWAS) or just printing the P value on top of each bar.
How were the 50 traits tested for genetic correlation selected?

In the initial paragraph of the PheWAS section of the results, it would be helpful to note the number of individuals with BPD in each of the cohorts, so the reader is aware that they were not excluded.
In Fig 5, would it be possible to size the triangles by their effect size to incorporate additional information? (Mood disorders are most significantly associated in with the BPD PGS in both cohorts, which is likely because they are common, whereas other phenotypes had much larger effect sizes, as shown in the supplementary tables).

Methods
In the gene-set tests, was there are minimum number of genes required in the set to ensure a sufficiently powered test?

Which version of the phecode map was used?

How were the controls in the pheWAS defined – all non-cases, or those with 0 ICD codes, so that individuals with 1 ICD code were excluded from the analysis?

Reviewer #2 (Remarks to the Author):

This manuscript reports on the largest genome-wide association study (GWAS) of borderline personality disorder (BPD) to date. While the study makes an important contribution to understanding the genetic architecture of BPD, I have identified several methodological concerns that should be addressed before publication:

1. Incomplete reporting of meta-analysis methodology:
- The authors do not specify whether a fixed-effects or random-effects model was used.
- The choice of model can impact results and interpretation, particularly in the presence of between-study heterogeneity.
- Clarification on the chosen model and assessment of heterogeneity is needed for proper interpretation of the findings.

2. Absence of power calculations:
- The authors do not present power calculations, despite the substantial case-control imbalance (12,339 cases vs. over 1 million controls).
- Power calculations are essential for evaluating the minimum detectable effect sizes and the impact of the imbalance on the study's ability to detect associations.
- Including power calculations would allow readers to better assess the reliability and interpretability of the findings.

3. Lack of independent replication:

- The study does not include an independent replication sample to validate the six genome-wide significant loci.
- While the authors conduct several validity analyses (genetic correlations, PheWAS, polygenic scoring), these do not substitute for formal replication.
- Given the large sample size, the authors could have held out a portion for validation or used an external dataset (e.g., UK Biobank, deCODE) for replication.
- Including replication would strengthen the credibility and conclusiveness of the findings.

Addressing these methodological concerns would enhance the rigor, reliability, and impact of the study. I recommend that the authors provide clarification on the meta-analysis model, include power calculations, and consider adding an independent replication sample. With these improvements, the manuscript would make a more substantial contribution to our understanding of the genetic basis of BPD.

Reviewer #3 (Remarks to the Author):

A large genome-wide association study performed for borderline personality disorder, a relatively rare psychiatric trait of great interest due to the intersection with many outcomes and behaviors, from depression to suicide to personality. This is an important advance for the study of the underlying genetic architecture of BPD. The biggest concern is the relative lack of power and number of cases recruited.

Very large number of controls compared to a relatively modest number of cases. It's an important first step and it's good to see that significant SNP heritability is identified (and GWS loci are identified).

Could more care be taken to match the controls on demographics of the BPD cases, given the massive overrepresentation of controls? I found Tables S1 to be strangely truncated and confusing; can the control exclusion criteria be spelled out more clearly? In the sensitivity analyses, were individuals excluded from both the cases and controls?

Is it too early/ambitious to begin stratifying into sex? The primary analysis already seems to just barely cross the threshold into locus discovery. The female analysis resembles a slightly depowered version of the primary GWAS, with some GWS loci sinking just below the significance threshold as a function of power and not real sex differences.

The male loci are not extremely convincing, particularly the locus on chromosome 2. Why so few variants in LD? Is the lead SNP typed or imputed in most of the samples? Given the relatively small number of loci, regional plots might be useful to readers.

It is encouraging to see the consistency of the lead SNPs across the various stratified analyses

When conducting the liability scale adjustment to the heritability, what was the sample prevalence reported and the sample N provided to LDSC? Was the effective sample size used, or the raw N?

Reported inflation looks fine and is not a concern. What was the attenuation ratio of the LD score regression?

Could more be said about the selection criteria and justification for the traits selected for genetic correlations, other than picking a round number (50), with most traits that end up being statistically significant (43)? The selection seems somewhat arbitrary as written.

Is the MDD comorbidity known in the cases? Could this be reported?

I appreciate the historical perspective presented in the PRS Figure 3. Trait-specific progress is important to keep in mind in this kind of research.

Many of the techniques used are well-worn yet appropriate or cutting edge, a good mix of innovative and feasible for this kind of paper.

Excellent that X chromosome is included in this analysis. It is a critically understudied part of genomic analyses. Was the X chromosome incorporated into any of the heritability calculations?

Minor: It's standard to refer to X chromosome as '23' for various formatting reasons within the data/plink, perhaps it would be better labeled as 'X' in the plots for readers that aren't accustomed to that convention?

Could more be done to highlight the innovations in the analyses, aside from the largest available sample size? Maybe it makes sense given the available power, but many of the analyses prominently featured in writing or discussion are not particularly novel.

Reviewer #3 (Remarks on figshare data availability):

Data appears to be as described. Good practice to make the summary statistics available at such an early stage.

Version 1:

Decision Letter:

Our ref: NG-A67211R

31st July 2025

Dear Fabian,

Your revised manuscript "Genome-wide association study of borderline personality disorder identifies 11 loci and highlights shared risk with mental and somatic disorders" (NG-A67211R) has been seen by the original reviewers. As you will see from their comments below, they are satisfied with the revision and have no further requests, and therefore we will be happy in principle to publish your study in Nature Genetics as an Article pending final revisions to comply with our editorial and formatting guidelines.

We are now performing detailed checks on your paper, and we will send you a checklist detailing our editorial and formatting requirements soon. Please do not upload the final materials or make any revisions until you receive this additional information from us.

Thank you again for your interest in Nature Genetics. Please do not hesitate to contact me if you have any questions.

Sincerely,
Kyle

Kyle Vogan, PhD
Senior Editor
Nature Genetics
https://orcid.org/0000-0001-9565-9665

Reviewer #1 (Remarks to the Author):

The authors have done an excellent job of addressing all of my comments and I have no further questions or concerns.

Reviewer #2 (Remarks to the Author):

I am very appreciative of the authors' thorough attention to my concerns and comments. They have meticulously addressed all three points that I raised:

1. The meta-analysis methodology is now clearly specified as a fixed-effects model, with a comprehensive heterogeneity assessment included.
2. Power calculations have been incorporated, which have addressed the case-control imbalance and now provide a much clearer regarding the study's statistical power.
3. A second independent replication cohort has since been added, with replication analyses extended to the SNP level, confirming the robustness of the findings.

Overall, these revisions are meticulously documented in the revised manuscript. This significantly enhances the rigor and reproducibility.

Reviewer #3 (Remarks to the Author):

My comments have been adequately addressed. No more concerns.

Reviewer #3 (Remarks on figshare data availability):

Better than field standard.

Open Access This Peer Review File is licensed under a Creative Commons Attribution 4.0 International License, which permits use, sharing, adaptation, distribution and reproduction in any medium or format, as long as you give appropriate credit to the original author(s) and the source, provide a link to the Creative Commons license, and indicate if changes were made.
In cases where reviewers are anonymous, credit should be given to 'Anonymous Referee' and the source.
The images or other third party material in this Peer Review File are included in the article's Creative Commons license, unless indicated otherwise in a credit line to the material. If material is not included in the article's Creative Commons license and your intended use is not permitted by statutory regulation or exceeds the permitted use, you will need to obtain permission directly from the copyright holder.
To view a copy of this license, visit https://creativecommons.org/licenses/by/4.0/

Response Letter

We would like to thank the reviewers for their thoughtful and constructive comments as well as the overall positive evaluation of our manuscript. All points raised are considered in the following point-by-point response (in blue) and manuscript changes are also presented here (highlighted in bright yellow). We hope we have addressed the reviewers' concerns adequately and that the manuscript is now acceptable for publication.

Reviewer #1 (Remarks to the Author):

General comments:

Streit et al report the largest GWAS of borderline personality disorder (BPD) to date, including 12,339 cases and 1,041,717 controls of European ancestry. They find 6 GWS loci, SNP-h2 of 17.3% and PGS explaining ~4.6% of phenotypic variance. I appreciated that the X chromosome was included in the GWAS and that sex-specific GWAS were also presented. The study used genetic correlation analyses and PheWAS in two cohorts to identify phenotypes with shared genetic etiology with BPD, which highlighted other psychiatric disorders, suicide and self-harm related traits, and medical conditions including obstructive pulmonary disease and diabetes. Overall, this is a high-quality GWAS which makes a meaningful contribution to the literature on the etiology of BPD. I have two main comments:

Response:

We thank the reviewer for their detailed and helpful comments.

Comment 1:

The study would be enhanced by adding a single-cell gene expression enrichment analysis to gain insight into the brain cell-types most relevant for BPD.

Similarly, an enrichment analysis of genes expressed in the brain at different developmental timepoints would be of interest.

Response:

We have now added the enrichment of Brainspan data sets reflecting age groups and developmental stages to the tissue enrichment analysis as implemented in FUMA, and see some evidence of an influence in early expressed genes. We additionally added a single-cell enrichment analysis based on the Human Brain Atlas single-nucleus RNA sequencing dataset by Siletti et al.. We found significant enrichment in *medium spiny neurons*, and *LAMP5-LHX6 and Chandelier* cells. We now present and discuss this data in the revised version of the manuscript.

Manuscript Changes:

Results:

> *"Enrichment analysis*
>
> *Enrichment of gene-sets and tissue expression using Genotype-Tissue Expression (GTEx; version 8) data for 53 tissue types[24] and expression data from 29 different ages and 11 general developmental stages (BrainSpan)[25] was tested with MAGMA (version 1.07)[21] as implemented in FUMA[22]: One significant gene-set was identified: the S1P-S1P3 (Sphingosine-1-phosphate - sphingosine-1-phosphate receptor 3) pathway[26] (p_{adj}=0.018; Supplementary Table S15). Nominally significant enrichment was observed in several GTEx tissue types, with the most significant enrichment in cerebellar tissue (not significant after correction for multiple testing; all p_{adj}>0.32; Supplementary Fig. S44, Supplementary Table S16). The analysis using the two BrainSpan data sets indicated the strongest enrichments 8–21 weeks post conception (not significant after correction; all p_{adj}>0.09), and in early to mid prenatal phases respectively (p_{adj}<0.025; Supplementary Fig. S45&46, Supplementary Table S17&18). Analyses based on Human Brain Atlas single-nucleus RNA sequencing data[27], showed SNP-h² enrichment using stratified LDSC[28,29] in 2 of the 31 tested superclusters ("medium spiny neurons";"LAMP5-LHX6 and Chandelier cells"; p_{adj}<0.0064; Supplementary Fig. S47, Supplementary Table S19)."*

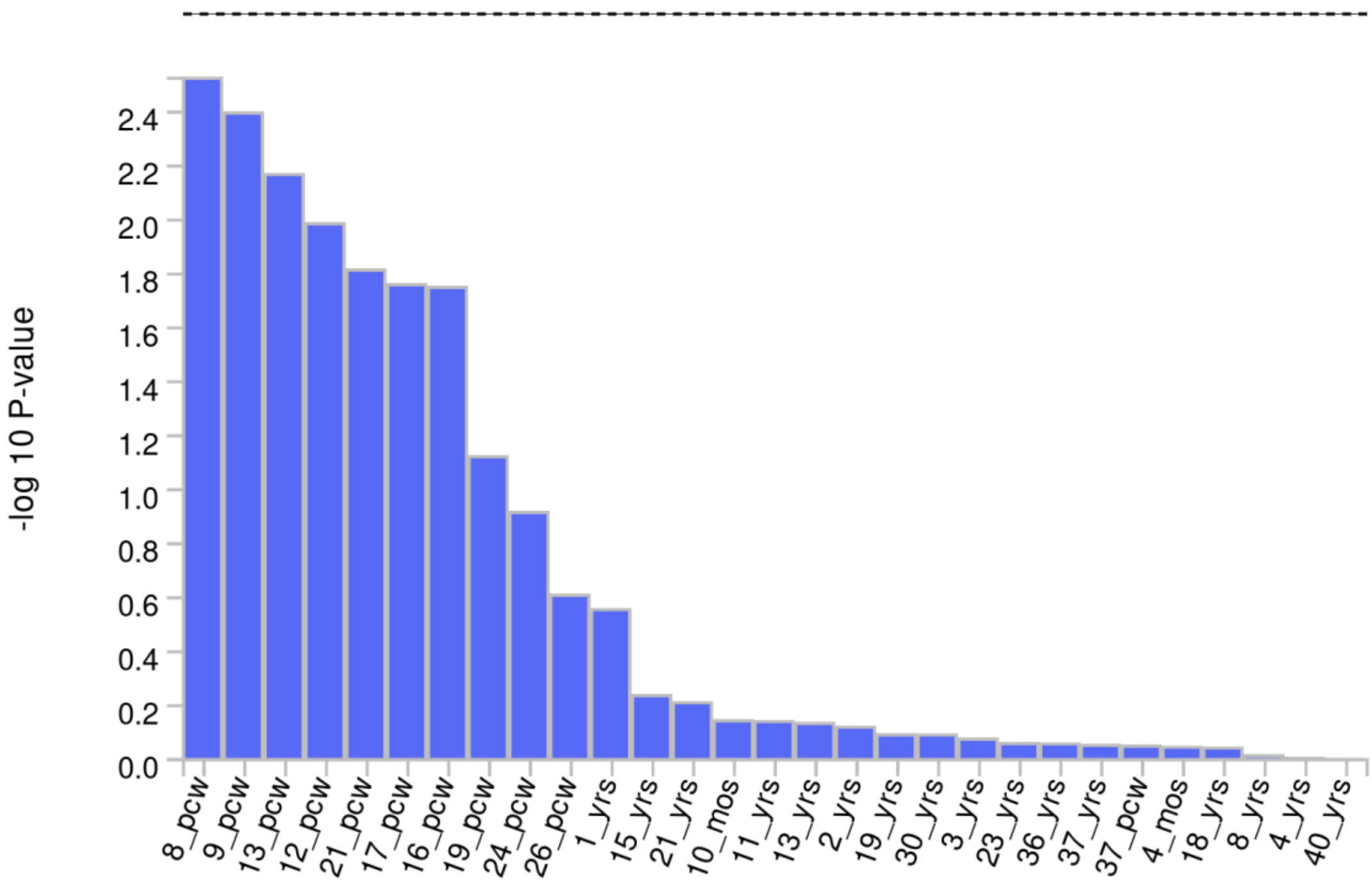

Figure S45: BrainSpan age groups: enrichment of expression of genes associated with BPD as implemented in FUMA. -log10 p-values are indicated on the y-axis.; pcw = post-conceptional Weeks; mos = months; yrs = years

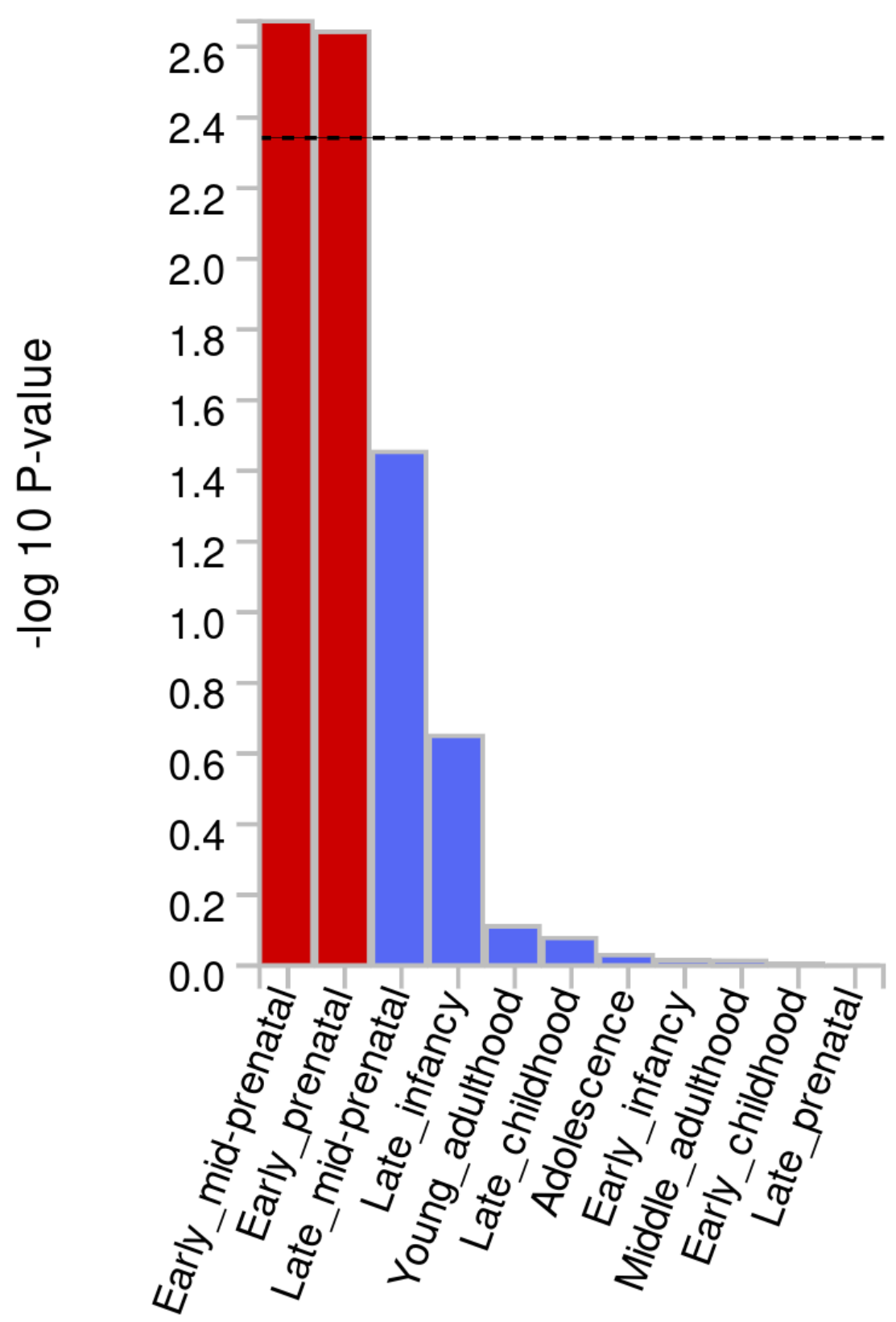

Figure S46: BrainSpan developmental stages: enrichment of expression of genes associated with BPD as implemented in FUMA. -log10 p-values are indicated on the y-axis. Red bars indicate significance after correction for multiple testing.

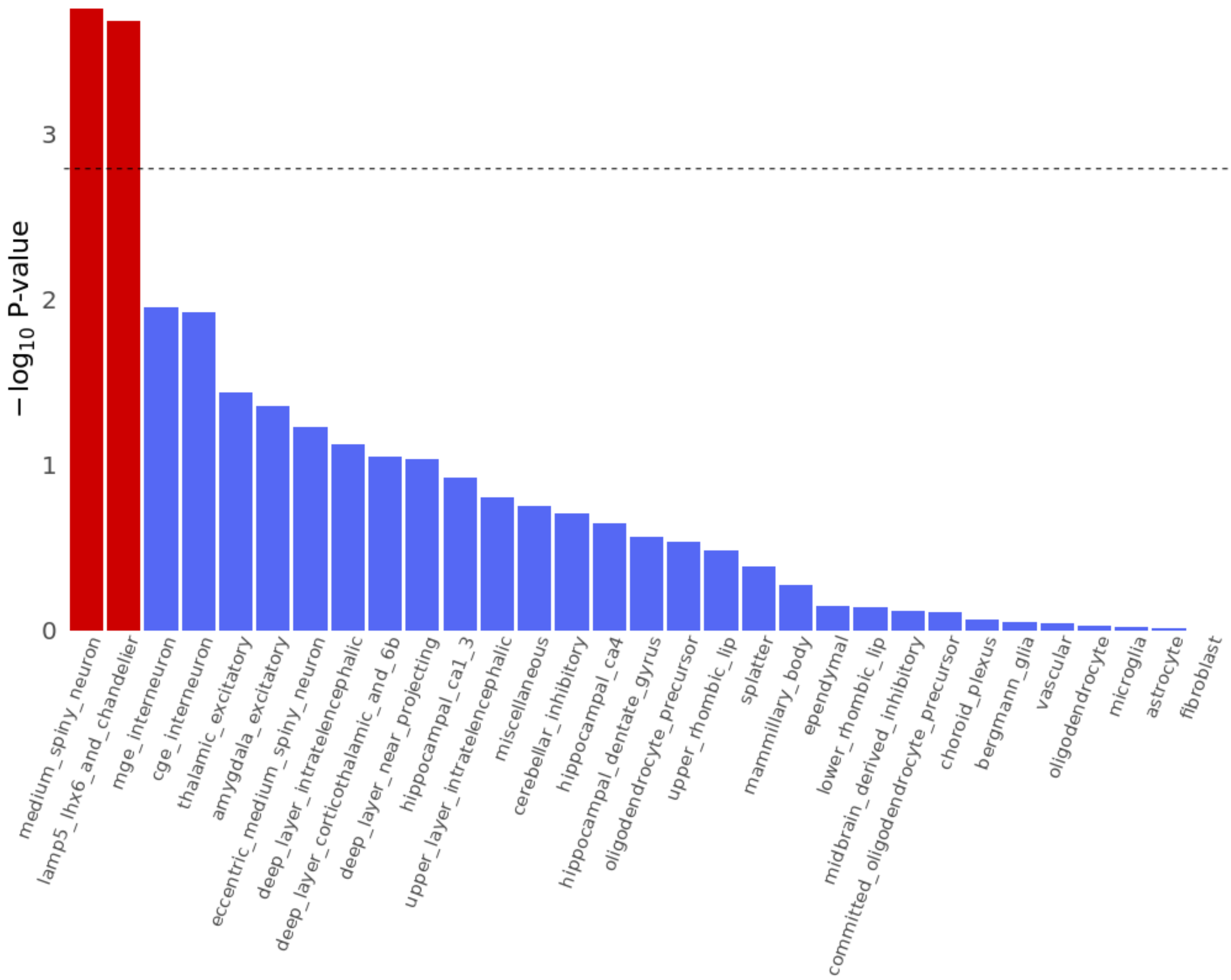

Figure S47: SNP-h² enrichment for BPD on the supercluster-level based on Human Brain Atlas single-nucleus RNA sequencing data. -log10 p-values are indicated on the y-axis. Red bars indicate significance after correction for multiple testing.

Discussion:

> *"This study is the first to systematically use genetic association data to investigate expression enrichment and potential drug repurposing for BPD, offering initial insights: The results suggest the importance of genes expressed in the brain and during early prenatal development. Single-cell analyses revealed significant SNP-heritability*

enrichment in genes expressed in the clusters "medium spiny neurons" as well as "LAMP5-LHX6 and Chandelier cells". Both clusters have previously been demonstrated to be enriched in mental health phenotypes in a systematic analysis, namely in schizophrenia, IQ, educational attainment, and neuroticism[29], with "medium spiny neurons" additionally showing enrichment in MDD[29], and the "Lamp5-LHX6 and Chandelier cell" cluster being significantly enriched in the most recent bipolar disorder GWAS[67]. Together, these findings suggest that specific neuronal cell types with established relevance for psychiatric disorders may also play a role in BPD."

Methods:

"Tissue enrichment expression was carried out using Genotype-Tissue Expression (GTEx; version 8) data for 53 tissue types[24], and data from BrainSpan, representing 29 different ages and 11 general developmental stages[25] as implemented in FUMA (version 1.5.2)[22]. In addition, we utilized the Human Brain Atlas dataset containing single-nucleus RNA sequencing data from approximately 3.369 million nuclei derived from adult post-mortem brain samples, covering 106 anatomical sections[27]. We used the cell-types at the level of 31 "superclusters", and maintained the existing nomenclature for cell-types. We calculated expression proportion values for each cell-type ("gene specificity", described in detail in[29] and[98]), resulting in ~1300 genes per cell-type. We subsequently tested the enrichment of heritability in these gene sets using stratified LDSC [28], adjusting for the 53 baseline annotations. To adjust for multiple hypothesis testing in the tissue expression analyses, we applied a Bonferroni p-value threshold adjusted of the respective number of tested tissues."

Comment 2:

The drug-target analyses were performed by investigating 16 genes (implicated in GWS loci or gene-based tests) for drug tractability or being existing drug targets using online databases. It would be valuable to test enrichment across the entire genome in the targets of existing medications or classes of medications here as well. When focusing on 16 genes, the search space is very small, whereas the polygenic signal from this GWAS is quite robust.

Response:

We thank the reviewer for the suggestions. We have now run additional analyses using a recently developed tool, which is MAGMA based and uses the genome-wide association signal to test for drug target enrichment, DRUGSETS. (https://www.medrxiv.org/content/10.1101/2022.09.06.22279660v2)

The top hits of the analysis indicate medications for neurological conditions (safinamide, gabapentin, lomerizine, oxcarbazepine), pain (prilocaine, tetracaine), and include acamprosate, a medication to maintain abstinence from alcohol in alcohol dependent patients, but also include medications for metabolic/somatic conditions. However, no drug reached significance after correction for multiple testing (735 drugs tested).

Please find the top ten drugs below :

**DRUG**	**NGENES**	**BETA**	**P**	**Clinical_Indication**
safinamide	15	0.025816	0.000244	Parkinson's disease
gabapentin	39	0.022784	0.000649	Restless leg syndrome, seizures, shingles
dehydroepiandrosterone	49	0.019812	0.002408	Menopause
oxcarbazepine	20	0.019331	0.003986	Seizures
acamprosate	23	0.01936	0.006844	Abstinence from alcohol
tolazamide	7	0.017756	0.007267	Diabetes mellitus
prilocaine	11	0.019432	0.009896	Anesthetic
tetracaine	11	0.019432	0.009896	Anesthetic
miglitol	5	0.016806	0.012465	Diabetes mellitus
niraparib	9	0.016809	0.016779	Primary peritoneal cancer (PPC)

We now present these analyses in the manuscript, results, methods and discussion sections (The complete results including all 735 tested drugs has now been added as Supplementary table S20):

Manuscript Changes:

Results:

"In an additional analysis with DRUGSETS[30], based on MAGMA, testing the enrichment of the GWAS associations in 735 drug–gene sets, the most significant drugs included medications for neurological conditions (safinamide, gabapentin, lomerizine, oxcarbazepine), pain (prilocaine, tetracaine), and alcohol-dependence (acamprosate), but also medications for metabolic/somatic conditions (Supplementary Table S20; all $p_{adj}>0.18$*)."*

Methods:

"In addition to these approaches focussing on the prioritized 16 genes, we explored potential drug targets with the DRUGSETS approach[30], which integrates the genome-wide association signal. Using this approach we tested drug–gene sets, compiled from the Clue Repurposing Hub and the Drug–Gene Interaction Database, each comprising the genes whose protein products are known to be targeted by or to interact with a given drug. We then conducted a competitive gene-set analysis in MAGMA v1.08, conditioning on a background set of all 2,281 drug-target genes, to identify drug-gene sets significantly associated with BPD. To account for multiple testing across the 735 gene sets analyzed, we applied a Bonferroni threshold of $p<0.05/735 = 6.80\times10^{-5}$*."*

Discussion:

"The genome-wide DRUGSETS analysis indicated drugs with high biological plausibility, including medication for neurological disorders and pain, albeit our results were not statistically significant after correction for multiple testing and should be interpreted with caution."

Comment 3:

Introduction

The characteristics of BPD were described but including the specific diagnostic criteria and further information on the psychiatric and general medical comorbidities would be helpful. For example, it wasn't clear to me why the sensitivity analyses excluding BIP and SCZ were performed but some additional background information would set this up (e.g., are these the disorders most commonly comorbid with BPD, is misdiagnoses between BPD and BIP/ SCZ common, or was this to match the phenotyping of the biobanks with the clinical samples, which typically exclude BIP/SCZ as noted in the discussion?).

Response:

We thank the reviewer for the comment. We now give more information in the introduction and clarify the sensitivity analysis in the results section:

Manuscript Changes:

Introduction:

> *"Borderline Personality Disorder (BPD) is a mental disorder characterized by pervasive instability in emotions, interpersonal relationships, and self-image, and by impulsive behavior (symptoms see Supplementary Methods)[1,2]. BPD has a prevalence of 0.92–1.90% in Western countries[3], with symptom onset typically occurring during adolescence. Women are more frequently diagnosed with BPD than men by a ratio of ~3:1, for which a substantial contribution of diagnostic as well as selection bias has been postulated[4,5]. Individuals with BPD display high rates of self-harm, suicidal ideation, and suicide attempts. BPD shows substantial symptom overlap and comorbidity with other mental disorders[5,6], and comorbidity with neurological and somatic health conditions[6,7]. While some psychotherapies are effective in treating BPD[8], no psychopharmacological treatments are FDA- approved specifically for BPD[2,9].*
>
> *In addition to environmental risk factors such as early interpersonal trauma[10,11], genetic factors substantially contribute to disorder risk. Twin and family studies estimate the heritability of BPD to be 46–69%[12,13], and demonstrate that the genetic risk for BPD is*

partially shared with other mental disorders, but as well as with continuous traits, e.g. the Big Five personality traits[14,15]. However, a systematic assessment of shared genetic risk with a broad range of disorders and traits is missing."

Results:

"Additionally, we performed meta-analyses stratified by sex (female-only; male-only) as well as a sensitivity analysis excluding subjects with a history of BIP or SCZ—conditions commonly excluded in BPD studies—from the studies providing summary statistics (Supplementary Tables S3–S5)."

...

"Sensitivity analysis excluding BIP&SCZ

"Dedicated studies of BPD often exclude subjects with schizophrenia and bipolar disorder. To explore the influence of comorbidity with these severe mental disorders on our results, as a sensitivity analysis, association analyses were performed excluding subjects with a history of BIP or SCZ from the studies providing summary statistics (sensitivity analysis: N_{cases}=8,618, $N_{controls}$=1,027,690; Supplementary Tables S3–S5). In the case of the cohorts, where the control population was not filtered for BIP and SCZ in the main analysis (Copenhagen Hospital Biobank and Danish Blood Donor Study; deCODE; Mayo Clinic Biobank; FinnGen), subjects with BIP or SCZ were also excluded from the controls for the sensitivity analysis."

Comment 4:

What was the se on the intercept and the LD Score Regression attenuation ratio?

Response:

For the main analysis, the intercept and SE were 1.0293 (0.0085) and the attenuation ratio was 0.1056 (0.0306). With an intercept in the range of 1.0 - 1.05 and an attenuation ratio of ~.1, we conclude that the large part of our association signal is not attributable to confounding. We now report this in the results, discussion, and methods section.

Manuscript Changes:

Results:

“We observed an SNP-heritability of 28.4% (95% confidence interval (CI) [24.9%, 31.8%]), corresponding to an SNP-heritability of 17.2% (95%-CI [15.2%, 19.4%]) on the liability scale (assuming a population prevalence of 1.5%)[3]. The linkage disequilibrium score regression (LDSC)[20] intercept was 1.03 (standard error (SE)=0.0085), with an attenuation ratio of 0.11 (SE=0.031), and we observed a genomic inflation factor (λ_{GC}) of 1.22 (λ_{1000}=1.01). There was a high genetic correlation between the studies with individual-level data and those providing summary statistics (r_g=0.85, 95%-CI [0.68, 1.03]), which was significantly smaller than 1 (p=0.038).“

Discussion:

“A substantial proportion of the heritability observed in family and twin studies (46–69%)[12,13] was explained by common genetic variation (estimated SNP-heritability of 17%). This is consistent with GWAS in other psychiatric disorders, where SNP-heritability often accounts for approximately a third of the estimates from twin and family studies[16]. The substantial contribution of common genetic variation to BPD is also supported by the leave-one-out PGS analyses, predicting a weighted average of 4.6% of the variance on the liability scale. Importantly, the low LDSC intercept (1.02) and the attenuation ratio of 11% indicate the association is largely driven by the polygenic signal and not by confounding.”

Methods:

“SNP-heritability, based on the autosomal markers, was estimated using LDSC[20], both as the observed SNP-heritability and as the SNP-heritability converted to the liability scale, using the N_{eff} (2×21,617=43,234)[96], a corresponding sample prevalence of 0.5, and assuming a population prevalence of 1.5%[3] for the main analysis. For the sex-stratified GWAS, we used sex-specific population prevalences of 2.25% in females and 0.75% in males for the conversion to the liability scale, corresponding to the previously described female:male ratio of 3:1[4,5]. To assess the contribution of confounding to the observed signal, we calculated the attenuation ratio ($\frac{(Intercept - 1)}{(mean\ \chi 2^{\blacksquare} - 1)}$).”

Comment 5:

Where genetic correlations are reported between cohorts with individual-level data and summary statistics, or between GWAS in males and females, I recommend adding a test for whether the genetic correlation is significantly different from 1.

Response:

We have now applied the block jackknife method to test the difference of the genetic correlations from 1 (and to test differences in SNP-heritability). Here the genetic correlation of males vs. females was not significantly different from 1, but the genetic correlation between the cohorts with individual-level data vs. summary statistics differed significantly from 1. However, it has to be noted, that the z-score of the heritability of the male-only analysis was below 4, a threshold often recommended for reliable interpretation of the results using LDSC. We now describe this approach and the results in the manuscript:

Manuscript Changes:

Results:

> *"There was a high genetic correlation between the studies with individual-level data and those providing summary statistics (r_g=0.85, 95%-CI [0.68, 1.03]), which was significantly smaller than 1 (p=0.038)."*
>
> …
>
> *"We carried out sex-stratified GWAS meta-analyses for male (N_{cases}=2,260, $N_{controls}$=485,444) and female subjects (N_{cases}=10,025, $N_{controls}$=547,333) (Supplementary Tables S3&S4).*
> *We used sex-specific population prevalences of 2.25% for females and 0.75% for males to convert heritability estimates to the liability scale[4,5]. For females, we observed an SNP-heritability of 30.3% (95%-CI [26.1%, 34.5%]), corresponding to 20.5% (95%-CI [17.7%, 23,4%]) on the liability scale. For males, the observed SNP-heritability was*

19,8% (95%-CI [7.9%, 31.6%]), corresponding to 10.2% (95%-CI [4.1%, 16.3%] on the liability scale, which was significantly lower than in females (p=0.00090).

There was a high genetic correlation between the two analyses (r_g=0.80, 95%-CI [0.53, 1.07]), which did not differ from 1 (p=0.055).”

Discussion:

“We present the first GWAS to include both sex-stratified and X-chromosome analyses — an initial step towards investigating the sex-specific genetic architecture of the disorder, which is relevant concerning the sex differences in BPD prevalence and presentation. We observed a substantially higher SNP-heritability in females, which has also been described for the two mental disorders showing the strongest genetic correlation with BPD–PTSD and depression[62]. The lead SNPs of the main GWAS, however, showed the same direction and similar effect sizes in the stratified analyses in both sexes, and the genetic correlation of the sex-stratified GWAS was high and did not differ from 1. However, it must be noted that the sex-stratified analyses had limited statistical power, particularly the male-only analysis, which was also indicated by a z-score of the SNP-heritability under 4[63]. Nevertheless, we consider the sex-stratified analysis an important first step and provide it as a resource. Sex-stratified analyses in larger samples may help to elucidate sex-specific risk factors of BPD[64].”

Methods:

“To test whether the SNP-heritability estimates of the sex-stratified GWAS differed, and to test whether the genetic correlations between GWAS subsets (males vs. females; individual-level vs summary cohorts) differed from 1, we used the block jackknife extension of LDSC[20], which has previously been described[97].”

Comment 6:

Figure 2 – what is the difference between the loci in red and orange?

Response:

The color coding (orange vs. red) was intended to differentiate between loci in chromosomes with odd and even numbers. As only one locus was on a chromosome with an even number

we agree that this approach was not helpful. As the revised version of the manuscript now includes a replication analysis using the All of Us dataset, we amended the figure, to represent the *p*-values of the main analysis, and to additionally display the *p*-values in the combined analysis (original meta-analysis plus replication dataset), for the loci that reach genomewide significance either in the main or in the combined analysis.

Manuscript Changes:

Figure:

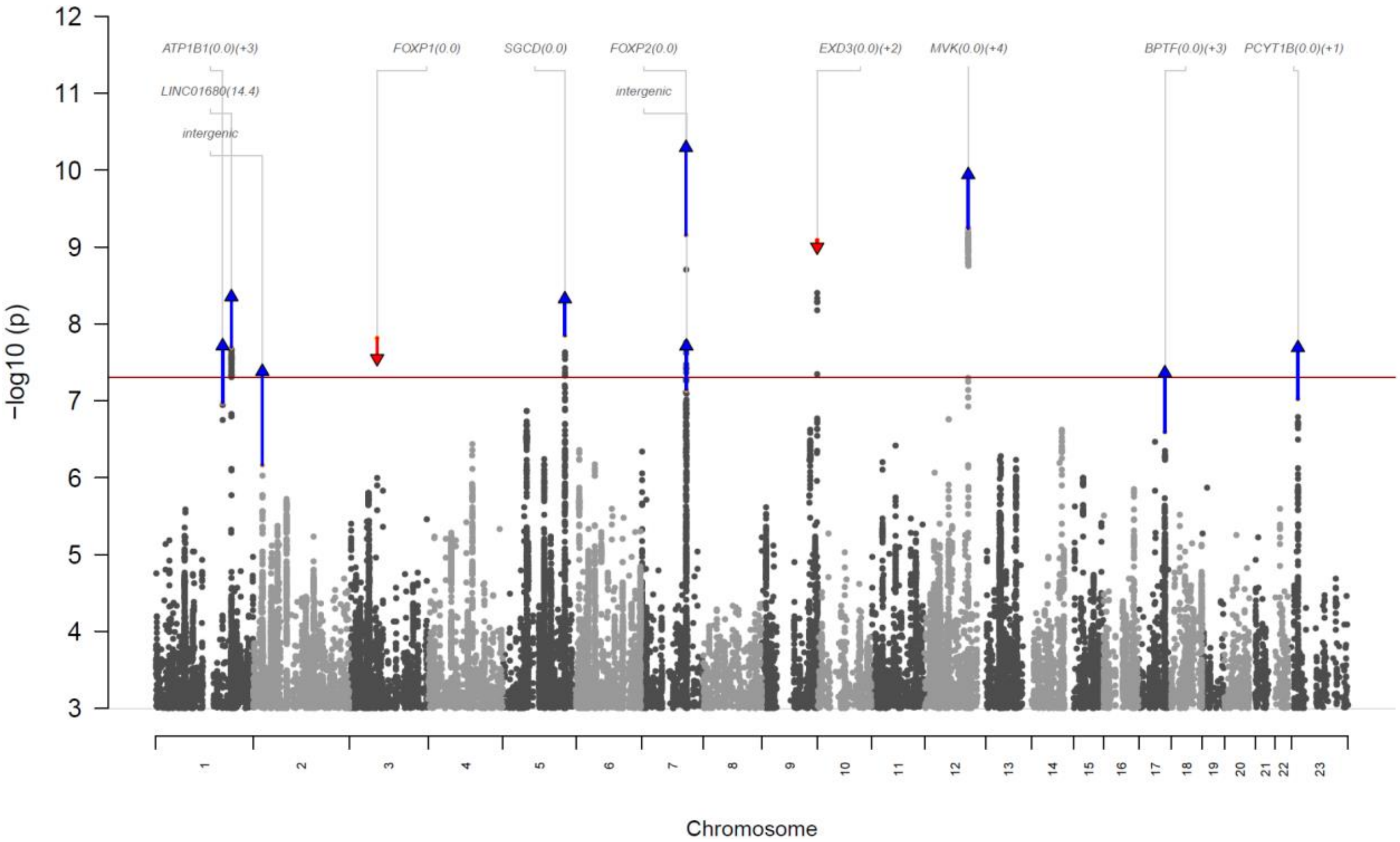

Figure 2: Manhattan plot of the genome-wide association study (GWAS) of borderline personality disorder (BPD). *The -log10 p-value for each single nucleotide polymorphism (SNP) in the discovery GWAS (N_{cases}=12,339, $N_{controls}$=1,041,717) is indicated on the y-axis (chromosomal position shown on the x-axis). The red line horizontal indicates genome-wide significance ($p<5\times10^{-8}$). Index SNPs representing independent genome-wide significant associations either in the discovery GWAS or the combined analysis (N_{cases}=13,024, $N_{controls}$=1,149,467) are highlighted (upward-pointing blue triangle: increased significance in combined analysis, downward-pointing red triangle: reduced significance in combined analysis). Combined p-values are plotted for lead loci and vertical lines indicate the p-value change from the discovery to the combined analysis.*

Comment 7

Table 1 – which allele do the test statistics correspond to?

Response:

The test statistic reported in Table 1 corresponds to allele 1 (A1). We now state this in the table legend. We have additionally added information on the results of the SNPs in the replication sample and in the combined meta-analysis. Additional SNPs were included in the table if they reached genome-wide significance in the combined analysis.

Manuscript Changes:

Results:

Table 1: Lead 11 genome-wide significant SNPs associated (p<5×10^{-8}) with BPD

Locus	CHR	SNP	BP	Locus (start..stop)	A1/ A2	FRQ case	FRQ control	OR	SE	*p*	OR repl.	*p* repl.	OR comb.	*SE comb.*	*p* comb.	Mapped Genes
comb. 1	1	rs2143288	169084340	169082364..169084344	G/A	0.858	0.868	0.89	0.022	1.06×10^{-7}	0.86	0.035	0.89	0.021	**1.23×10^{-8}**	*LINC00970(0.0),LOC101928596(0.0), ATP1B1(0.0),NME7(0.0)*
1	1	rs9970854	191125935	191005935..191148335	G/A	0.595	0.597	0.92	0.015	**1.99×10^{-8}**	0.93	0.183	0.92	0.014	**7.42×10^{-9}**	*LINC01680(14.4)*
comb. 2	2	rs12466671	22583435	22548735..22606235	A/G	0.587	0.599	0.93	0.015	6.80×10^{-7}	0.85	0.002	0.92	0.014	**2.16×10^{-8}**	-
2	3	rs6549383	71266885	71261185..71267720	T/C	0.503	0.503	1.09	0.015	**1.53×10^{-8}**	1.02	0.664	1.08	0.014	**2.45×10^{-8}**	*FOXP1(0.0)*
3	5	rs2135029	155856538	155743538..155868438	A/G	0.594	0.599	0.92	0.014	**1.41×10^{-8}**	0.92	0.141	0.92	0.014	**4.30×10^{-9}**	***SGCD(0.0)***
4	7	rs4727799	114110568	114021868..114224568	T/C	0.663	0.67	1.10	0.015	**6.92×10^{-10}**	1.15	0.015	1.10	0.015	**4.05×10^{-11}**	***FOXP2(0.0)***
comb. 3	7	rs10953781	114974695	114963595..115112695	C/T	0.528	0.534	1.08	0.014	7.13×10^{-8}	1.06	0.313	1.08	0.014	**4.38×10^{-8}**	-
5	9	rs73581580	140251458	140242728..140278158	G/A	0.836	0.839	0.89	0.019	**8.09×10^{-10}**	0.98	0.798	0.89	0.019	**1.45×10^{-9}**	*NRARP(-4.8)*, ***EXD3(0.0),*** *NOXA1(16.4)*
6	12	rs7304862	109988891	114021868..114224568	G/T	0.529	0.513	1.09	0.014	**5.71×10^{-10}**	1.09	0.114	1.09	0.014	**1.68×10^{-10}**	*MYO1H(-52.7), KCTD10 (-23.5), UBE3B(0.0),* ***MMAB(0.0)***, ***MVK(0.0)***
comb. 4	17	rs7210027	65977053	65825053..66098053	T/C	0.77	0.782	0.92	0.017	2.54×10^{-7}	0.87	0.031	0.91	0.016	**3.12×10^{-8}**	*BPTF(0.0),C17orf58(0.0),KPNA2(4.7) ,LINC00674(70.6)*
comb. 5	X	rs5944622	24613189	24578589..24933189	T/C	0.57	0.58	0.93	0.013	9.47×10^{-8}	0.92	0.037	0.93	0.013	**2.05×10^{-8}**	*PDK3 (0), PCYT1B (0), PCYT1B−AS1 (+5.0), POLA1 (+48.8)*

Variants are sorted by chromosomal position. CHR=chromosome; Locus (start..stop)=start and end of the boundaries of the region within r^2 of 0.6 of the index SNP; A1=allele 1, A2=allele 2, FRQ=frequency of A1 in cases/controls; BP=base pair position; comb. = combined analysis; OR=odds ratio of A1; repl. = replication analysis; SE=standard error; SNP=single nucleotide polymorphism; BPD=borderline personality disorder; Mapped genes=genes within r^2 of 0.6 and 50 kb of the index SNP (distance to the 50kb for boundary of the lead SNP in kb is indicated in brackets); Genes in bold were also identified by the gene-based association tests. *P* values in bold indicate genome-wide significance ($p<5x10^{-8}$). OR, SE, and p are reported for the main analysis (N_{cases}=12,339, $N_{controls}$=1,041,717), the replication analysis (repl.) N_{cases}=685, total $N_{controls}$=107,750), and the combined (comb.) meta-analysis (N_{cases}=13,024, $N_{controls}$=1,149,467) of the two datasets.

Comment 8:

What were the SNP-h2 estimates of BPD in males and females and were they significantly different from each other?

Response:

We now additionally report the SNP-h² estimates for males and females. Indeed we observe a higher h² for the females.

Manuscript Changes:

Results:

> *"We carried out sex-stratified GWAS meta-analyses for male (N_{cases}=2,260, $N_{controls}$=485,444) and female subjects (N_{cases}=10,025, $N_{controls}$=547,333) (Supplementary Tables S3&S4).*
> *We used sex-specific population prevalences of 2.25% for females and 0.75% for males to convert heritability estimates to the liability scale[4,5]. For females, we observed an SNP-heritability of 30.3% (95%-CI [26.1%, 34.5%]), corresponding to 20.5% (95%-CI [17.7%, 23,4%]) on the liability scale. For males, the observed SNP-heritability was 19,8% (95%-CI [7.9%, 31.6%]), corresponding to 10.2% (95%-CI [4.1%, 16.3%] on the liability scale, which was significantly lower than in females (p=0.00090)."*

Discussion:

> *"We present the first GWAS to include both sex-stratified and X-chromosome analyses — an initial step towards investigating the sex-specific genetic architecture of the disorder, which is relevant concerning the sex differences in BPD prevalence and presentation. We observed a substantially higher SNP-heritability in females, which has also been described for the two mental disorders showing the strongest genetic correlation with BPD–PTSD and depression[62]. The lead SNPs of the main GWAS, however, showed the same direction and similar effect sizes in the stratified analyses in both sexes, and the genetic correlation of the sex-stratified GWAS was high and did not differ from 1. However, it must be noted that the sex-stratified analyses had limited*

statistical power, particularly the male-only analysis, which was also indicated by a z-score of the SNP-heritability under 4[63]. Nevertheless, we consider the sex-stratified analysis an important first step and provide it as a resource. Sex-stratified analyses in larger samples may help to elucidate sex-specific risk factors of BPD[64].”

Methods:

“For the sex-stratified GWAS, we used sex-specific population prevalences of 2.25% in females and 0.75% in males for the conversion to the liability scale, corresponding to the previously described female:male ratio of 3:1[4,5]. To assess the contribution of confounding to the observed signal, we calculated the attenuation ratio ($\frac{(Intercept - 1)}{(mean\ \chi 2^{\blacksquare} - 1)}$).”

Comment 9:

In the PGS section of the results, it was not clear whether the R2 values in the text were on the liability scale and the values in Figure 3A are presented as Nagelkerke’s R2. Please present all results on the liability scale.

Response:

We have now clarified that the PGS results are all presented on the liability scale (previously this was only mentioned in the method section).

Manuscript Changes:

Results:

*“**Polygenic scoring***

BPD-PGS were calculated using PRS-CS[31] in the datasets where individual-level genotype information was available using leave-one-out summary statistics, excluding the respective sample from the discovery GWAS. In addition, we calculated PGS in the two independent replication data sets (Online Methods). For comparison, PGS were additonally calculated based on the first BPD GWAS from 2017[18]. The explained variance was converted to the liability scale (population prevalence = 1.5%[3]).”

We have also added this information to the labeling of axis y in Figure 3A, and the respective figure legend (see response to Comment 11).

Comment 10:

In the comparison of the OR for the top versus lowest decile, adding the CIs to the text would be helpful. I would also like to see a comparison of the top versus middle decile since the individuals in the lowest decile are just as much outliers in the population as those in the top decile.

Response:

We have now added the CIs to the text, and have added the middle 10% of the distribution as an additional reference.

Manuscript Changes:

Results:

> *"PGS explained a weighted average of 4.6% (AUC=66.0%) of the phenotypic variance on the liability scale (assuming a lifetime prevalence of 1.5%[3]) in the five datasets included in the discovery meta-analysis (Fig. 3, Supplementary Table S21). In the independent replication samples, PGS explained 3.1% of the variance (p=0.0041, AUC=62.4%) in Spain 2, and 2.6% in All of Us ($p=2.09x10^{-25}$, AUC=61.4%). The odds ratio for BPD case-control status comparing the highest PGS decile to the lowest decile was OR=6.61 (95%-CI [5.20, 8.41]) for PGS based on the current meta-analysis, compared to OR=2.09 (95%-CI [1.60, 2.72]) for PGS based on the 2017 GWAS. When comparing to the middle 10% of the distribution, we observed an OR of 2.37 (95%-CI [1.94, 2.89])) for the highest decile for PGS based on the current meta-analysis and an OR of 1.44 (95%-CI [1.05, 1.98]) for PGS based on the 2017 GWAS."*

Methods:

> *"To assess the association between the deciles based on PRS-CS and the actual case and control status, we conducted logistic regression analyses using the decile-based*

dummy variables for Q2 to Q10. The odds ratios (OR) for each decile were calculated by exponentiating the coefficients from the logistic regression model, controlling for PCs (1, 2, 3, 4, 5, 6, 8, 10, 12, 14, 15, and 20). For the reference comparison (Q1/Q1), an odds ratio of 1 was assigned, indicating no effect. The odds ratios for each of the remaining deciles (Q2 to Q10) were computed accordingly $OR_{Q1/Qj}=exp(\beta j)$ for $j=2,3,\ldots,10$. Additionally, 95%-CI for the odds ratios were calculated using the standard errors of the coefficients ($CI_{lower}=exp(\beta j-1.96\times SEj)$; $CI_{upper}=exp(\beta j+1.96\times SEj)$). As an additional measure of effect, the highest decile (Q10) was compared to the middle 10% of the distribution as reference, and OR and CI were calculated as described above."

Comment 11:

Figure 3A could be improved by using a color gradient for P value rather than the stars (a different shape could be used for the 2017 and 2024 GWAS) or just printing the P value on top of each bar.

Response:

We thank the reviewer for the comment. We have now added the exact p-values to the figure, and used the shape to differentiate between the two GWAS.

Manuscript Changes:

Results:

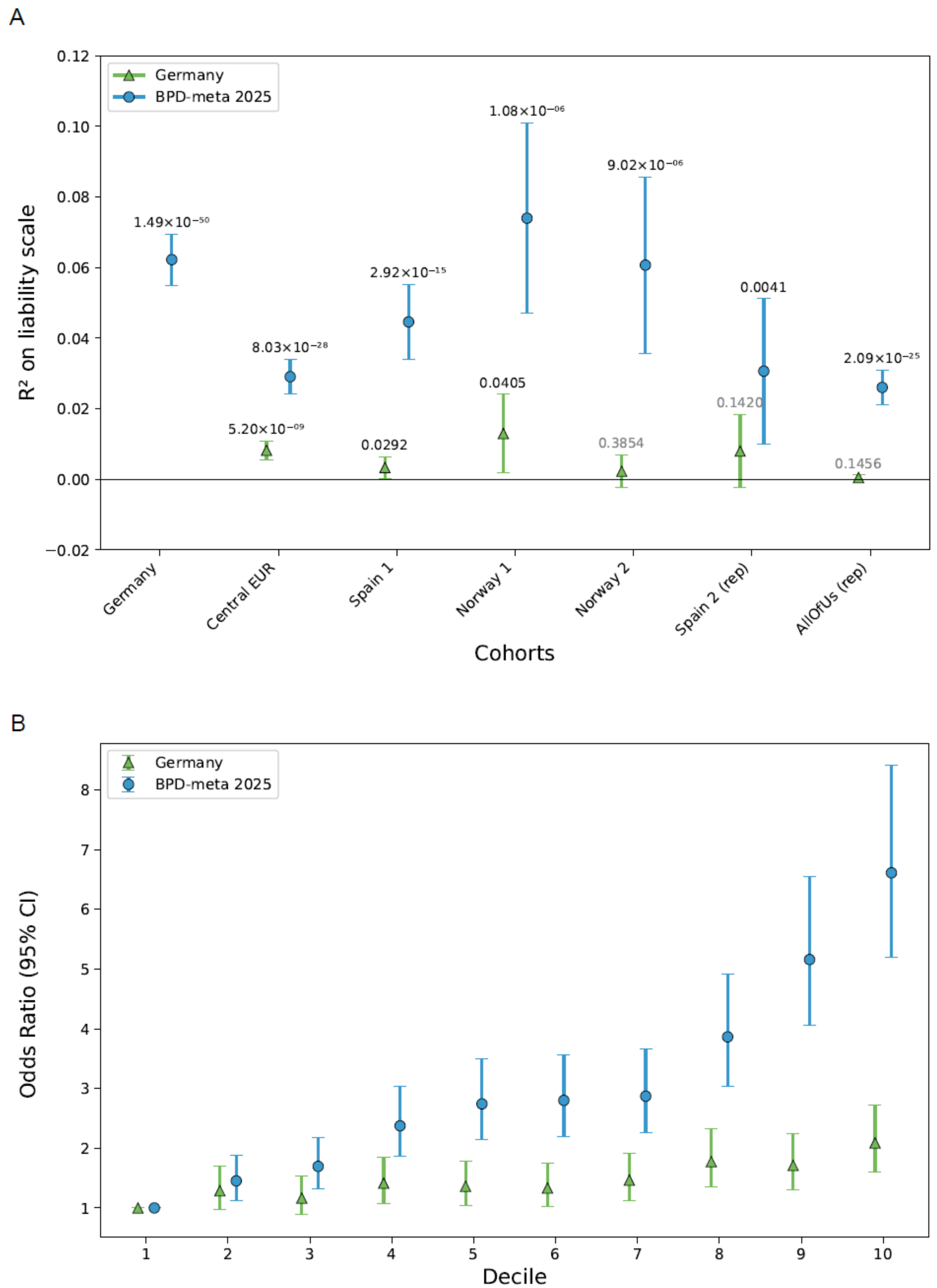

Figure 3: Polygenic score (PGS) analysis. A) The proportion of variance in case-control status explained by the PGS on the liability scale (y-axis; Liability R^2) B) Odds ratio for BPD by PGS deciles, with decile 1 as reference. Leave-one-out PGS were calculated for the datasets where individual-level genotype data was available, and in an additional independent PGS target sample (Spain2 (rep.), sample information in Supplementary Tables S1&S2) using PRS-CS. For each prediction, the respective dataset was excluded from the used discovery genome-wide association study (GWAS) meta-analysis. PGS in the independent target dataset were based on the complete current GWAS meta-analysis (blue circles: 2025 meta-GWAS). The explained variance was converted to the liability scale of the population assuming a lifetime disease risk of 1.5%. The analysis presented in Panel B excluded the All of Us dataset, as its individual-level data could not be merged with the other data sets due to data protection regulations. To visualize the increase in variance explained by the PGS, we also calculated PGS based on the first published BPD GWAS, consisting of the Germany sample (green triangles: 2017 GWAS) [18]. Significance: 1*: p<0.05; 2*: p<0.01; 3*: p<0.005; 4*: p<0.001; 5*: $p<1\times10^{-4}$; 6*: $p<1\times10^{-8}$; 7*: $p<1\times10^{-12}$, 8*: $p<1\times10^{-20}$, *: $p<1\times10^{-30}$

Comment 12:

How were the 50 traits tested for genetic correlation selected?

Response:

We thank the reviewer for the comment. The resulting round number was not intended but occurred by coincidence. We selected phenotypes of interest, i.e., disorders or traits reported to be associated with BPD, e.g. risk factors, or potential comorbid conditions. We decided on a rather targeted approach for the genetic correlations, to contrast with the rather exploratory and hypothesis-free PheWAS presented in the same manuscript. We have now added more information on the selection in the introduction and methods sections:

Manuscript Changes:

Methods:

> *"In a targeted approach, genetic correlations were calculated with 50 GWAS of a range of other disorders and traits relevant to BPD, selected based on reported phenotypic and genetic associations in the literature, theoretical consideration, and data availability. Those included GWAS of mental disorders, suicide, self-harm, trauma, substance use, physical health, pain, sleep, personality traits (Big Five GWAS including data from 23andMe, Inc.), and cognition (Supplementary Table S22). A Bonferroni p-value threshold, corrected for the number of respective tests was applied ($\alpha=0.05/50=1\times10^{-3}$)."*

We also explain more clearly now, that the targeted genetic correlations and the more exploratory PheWAS analyses represent complementary approaches to characterize the polygenic signal detected in our BPD GWAS.

Results:

> ***"Phenome-wide association studies (PheWAS)***
>
> *Complementing the targeted genetic correlation analysis, we characterized the genetic signal identified in the BPD GWAS, by performing two phenome-wide association studies (PheWAS), systematically exploring associations of the polygenic predisposition for BPD with over 1,000 diagnosis-based phenotypes across the diagnostic spectrum. For this, we tested the association of BPD-PGS with "phecodes" (Phecode Map 1.2), i.e., medical phenotypes based on International Classification of Disease (ICD) diagnoses documented in the electronic health records (EHR). PheWAS were calculated using data from the Vanderbilt University Medical Center Biobank (BioVU)[39] (66,325 subjects; 214 with the phecode 301.20: "antisocial/borderline personality disorder") and Hospital Episode Statistics (HES) in the UK Biobank (UKB) (316,635 subjects; 211 with the phecode 301.20). PGS were calculated using PRS-CS[34] and were based on summary statistics excluding the respective target sample from the discovery meta-analysis."*

Comment 13:

In the initial paragraph of the PheWAS section of the results, it would be helpful to note the number of individuals with BPD in each of the cohorts, so the reader is aware that they were not excluded.

Response:

We thank the reviewer for the comment. We have now added this information to the results and methods section:

Manuscript Changes:

Results:

"Phenome-wide association studies (PheWAS)

Complementing the targeted genetic correlation analysis, we characterized the genetic signal identified in the BPD GWAS, by performing two phenome-wide association studies (PheWAS), systematically exploring associations of the polygenic predisposition for BPD with over 1,000 diagnosis-based phenotypes across the diagnostic spectrum. For this, we tested the association of BPD-PGS with "phecodes" (Phecode Map 1.2), i.e., medical phenotypes based on International Classification of Disease (ICD) diagnoses documented in the electronic health records (EHR). PheWAS were calculated using data from the Vanderbilt University Medical Center Biobank (BioVU)[39] (66,325 subjects; 214 with the phecode 301.20: "antisocial/borderline personality disorder") and Hospital Episode Statistics (HES) in the UK Biobank (UKB) (316,635 subjects; 211 with the phecode 301.20). PGS were calculated using PRS-CS[34] and were based on summary statistics excluding the respective target sample from the discovery meta-analysis."

Methods:

"Phenome-wide association studies (PheWAS)

PheWAS in BioVU (EHR data)

Data from the Vanderbilt University Medical Center Biobank (BioVU) [39] were used to test the association of BPD-PGS with medical phenotypes based on two or more International Classification of Disease (ICD) diagnoses ("phecodes"; Phecode Map 1.2) documented in the electronic health records (EHR). For each phecode, a case was defined as having >=1 ICD code in the phecode group on >=2 distinct dates, and control was defined as having 0 codes in that phecode group. Individuals with only 1 single instance of a code in that phecode group were excluded from the case/control definition for that phecode. PGS were calculated in 66,325 unrelated subjects of European genetic ancestry (214 individuals with phecode 301.20 ("antisocial/borderline personality disorder") based on summary statistics excluding the BioVU samples from the discovery meta-analysis (12,024 cases; 1,038,567 controls). PGS were computed using a continuous shrinkage prior (CS) to SNP effect sizes using PRS-CS[34] and standardized to have a mean of 0 and a standard deviation of 1. Phecodes were tested for association with BPD-PGS when at least 100 cases were available using logistic regression models with sex (defined as sex reported at birth from the EHR), age, and

the first 10 genetic PCs as covariates. 1,431 phecodes were included, and a Bonferroni-corrected threshold of α=0.05/1,431=3.49×10^-5 tests was applied.

PheWAS in UKB

An additional PheWAS analysis was carried out using data from the UK Biobank (UKB). Here, PGS for BPD were calculated using PRS-CS based on summary statistics excluding the UKB samples from the discovery meta-analysis (12,157 cases; 1,039,897 controls) generated by excluding the UKB. The PheWAS was calculated using phecodes (Phecode Map 1.2) based on diagnoses recorded in Hospital Episode Statistics (HES) in a maximum sample of 316,635 subjects (211 individuals with phecode 301.20). For each phecode, a case was defined as having >=1 ICD code in the phecode group, and control was defined as having 0 codes in that phecode group. A total of 1,250 phecodes with at least 100 cases were tested for association using a Bonferroni-corrected threshold of α=0.05/1,250=4.00×10^-5 tests."

Comment 14:

In Fig 5, would it be possible to size the triangles by their effect size to incorporate additional information? (Mood disorders are most significantly associated with the BPD PGS in both cohorts, which is likely because they are common, whereas other phenotypes had much larger effect sizes, as shown in the supplementary tables).

Response:

We thank the reviewer for the suggestion. We have now adapted the figure accordingly

Manuscript Changes:

Figure:

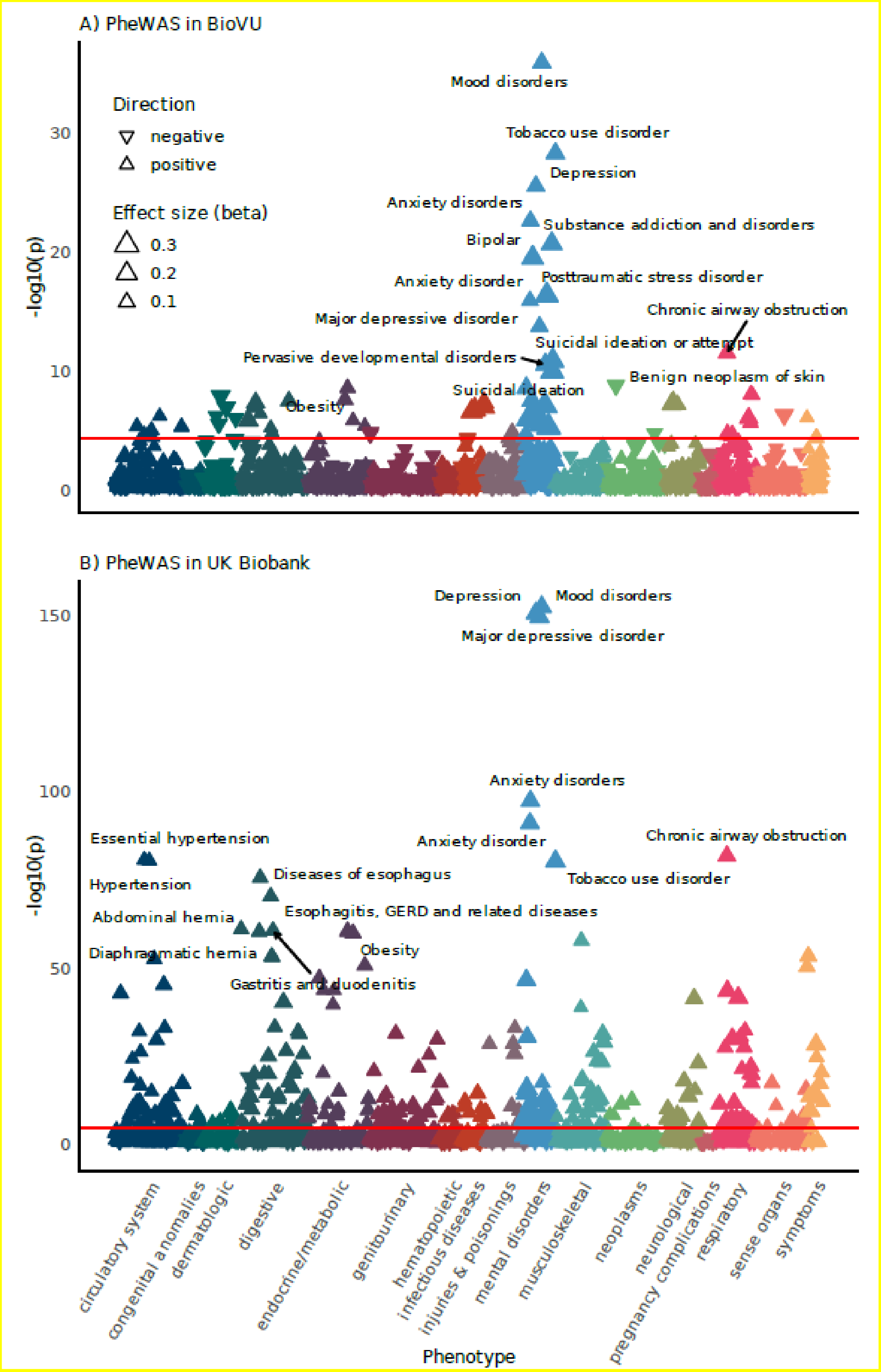

Figure 5: PheWAS of borderline personality disorder polygenic scores (BPD-PGS) in A) BioVU, and B) UKB: Association of BPD-PGS with 1,431 tested phecodes in BioVU and 1,250 Hospital Episode Statistics (HES) phecodes in UKB. Statistical significance (-log10(*p*)) is plotted on the y-axis and diagnoses are grouped by category. The red line indicates the significance threshold for Bonferroni correction for the tested phecodes (BioVU: 0.05/1,431=3.49×10^{-5}; UKB: 0.05/1,250=4.00×10^{-5}). The top 15 associations of each PheWAS are annotated. Upward-pointing triangles indicate positive associations, and downward-pointing triangles indicate negative associations. The size of the triangles indicates the effect size (beta).

Additionally, we now state more clearly that the associations were most significant with mood disorders, while the strongest effect size was observed for the phecode "antisocial/borderline personality disorder".

Results:

"PheWAS in BioVU (EHR data)

In the PheWAS analysis in the BioVU sample, 69 of the 1,431 tested diagnoses showed an association with BPD-PGS after Bonferroni correction (Supplementary Table S24). BPD-PGS showed the most statistically significant associations with codes from the mental disorders category (Extended Data Fig. 9), including mood disorders, substance use disorders, anxiety and PTSD, and suicidal ideation or attempt. Associations were also observed in other categories, including neurological (e.g., epilepsy) and somatic disorders (e.g., type 2 diabetes, chronic airway obstruction, hypertension), and pain disorders and symptoms.

PheWAS in UKB (HES data)

In the PheWAS analysis of HES codes in the UKB sample, 317 of the 1,250 tested diagnoses showed an association with BPD-PGS after Bonferroni correction (Supplementary Table S25). BPD-PGS showed the most statistically significant associations with codes from the mental disorders category (Extended Data Fig. 10), including mood disorders, tobacco use disorders, and anxiety disorders. As in BioVU, significant associations after Bonferroni correction were observed in other categories, including respiratory (e.g., chronic airway obstruction), digestive (e.g., esophagus, gastroesophageal reflux disease), circulatory, endocrine/metabolic, and neurological diagnoses, and pain disorders and symptoms.

Of the 69 diagnoses that were significant in the BioVU, 56 were also present with sufficient case numbers in the UKB. Of these, 51 showed effects in the same direction and 41 also reached significance after correction for multiple testing in the UKB

($p<0.05/1{,}225=4.08\times10^{-5}$). In both the BioVU and the UKB PheWAS, the phecode 301.20 ("antisocial/borderline personality disorder") showed the strongest effect size with the BPD-PGS ($OR_{BioVU}=1.41$; $OR_{UKB}=1.54$). "

Comment 15:

Methods

In the gene-set tests, was there a minimum number of genes required in the set to ensure a sufficiently powered test?

Response:

We did not limit the number of included genes in the tested gene sets in the initial analysis. For the revised version of the manuscript, we have now limited the gene sets to gene sets containing at least 20 mapped genes (n = 9,237) and adjusted the p-value threshold ($\alpha=0.05/9{,}237=5.4\times10^{-6}$). This change did not result in more significant associations after correction for multiple testing; one gene-set remained significantly associated. We have now updated this in the manuscript text and in Supplementary Table S14.

Manuscript Changes:

Methods:

"Gene-based, gene-set, and tissue enrichment tests were carried out using MAGMA (version 1.07) [26] as implemented in FUMA (version 1.5.2) [27]. Gene-based tests were performed using the summary statistics of the GWAS meta-analysis for a total of 19,843 genes, with SNPs assigned to genes based on their physical position. SNPs were included in the analysis using boundaries of 35 kilobases (kb) upstream and 10kb downstream of the genes. Gene-sets were tested by analyzing a total of 9,237 gene-sets (MSigDB v2023.1Hs; limited to gene sets with at least 20 mapped genes). For both gene-based and gene-set tests, a Bonferroni p-value threshold, corrected for the number of respective tests, was applied (gene-based $\alpha=0.05/19{,}843=2.5\times10^{-6}$; gene-set $\alpha=0.05/9{,}237=5.4\times10^{-6}$)."

Comment 16:

Which version of the phecode map was used? How were the controls in the pheWAS defined – all non-cases, or those with 0 ICD codes, so that individuals with 1 ICD code were excluded from the analysis?

Response:

In both cases, Phecode Map 1.2 was used. For BioVu, it was required that the phecode was recorded on at least two dates, while for UKB, the phecode was assigned with one occurrence only. These details are now mentioned in the methods and results section of the manuscript.

Manuscript Changes:

Results:

> *"Phenome-wide association studies (PheWAS)*
>
> *Complementing the targeted genetic correlation analysis, we characterized the genetic signal identified in the BPD GWAS, by performing two phenome-wide association studies (PheWAS), systematically exploring associations of the polygenic predisposition for BPD with over 1,000 diagnosis-based phenotypes across the diagnostic spectrum. For this, we tested the association of BPD-PGS with "phecodes" (Phecode Map 1.2), i.e., medical phenotypes based on International Classification of Disease (ICD) diagnoses documented in the electronic health records (EHR). PheWAS were calculated using data from the Vanderbilt University Medical Center Biobank (BioVU)[39] (66,325 subjects; 214 with the phecode 301.20: "antisocial/borderline personality disorder") and Hospital Episode Statistics (HES) in the UK Biobank (UKB) (316,635 subjects; 211 with the phecode 301.20). PGS were calculated using PRS-CS[34] and were based on summary statistics excluding the respective target sample from the discovery meta-analysis."*

Methods:

> *"Phenome-wide association studies (PheWAS)*
>
> ***PheWAS in BioVU (EHR data)***
>
> *Data from the Vanderbilt University Medical Center Biobank (BioVU) [39] were used to test the association of BPD-PGS with medical phenotypes based on two or more*

International Classification of Disease (ICD) diagnoses ("phecodes"; Phecode Map 1.2) documented in the electronic health records (EHR). For each phecode, a case was defined as having ≥1 ICD code in the phecode group on ≥2 distinct dates, and control was defined as having 0 codes in that phecode group. Individuals with only 1 single instance of a code in that phecode group were excluded from the case/control definition for that phecode. PGS were calculated in 66,325 unrelated subjects of European genetic ancestry (214 individuals with phecode 301.20 ("antisocial/borderline personality disorder") based on summary statistics excluding the BioVU samples from the discovery meta-analysis (12,024 cases; 1,038,567 controls). PGS were computed using a continuous shrinkage prior (CS) to SNP effect sizes using PRS-CS[34] and standardized to have a mean of 0 and a standard deviation of 1. Phecodes were tested for association with BPD-PGS when at least 100 cases were available using logistic regression models with sex (defined as sex reported at birth from the EHR), age, and the first 10 genetic PCs as covariates. 1,431 phecodes were included, and a Bonferroni-corrected threshold of $\alpha=0.05/1{,}431=3.49\times10^{-5}$ tests was applied."

PheWAS in UKB

An additional PheWAS analysis was carried out using data from the UK Biobank (UKB). Here, PGS for BPD were calculated using PRS-CS based on summary statistics excluding the UKB samples from the discovery meta-analysis (12,157 cases; 1,039,897 controls) generated by excluding the UKB. The PheWAS was calculated using phecodes (Phecode Map 1.2) based on diagnoses recorded in Hospital Episode Statistics (HES) in a maximum sample of 316,635 subjects (211 individuals with phecode 301.20). For each phecode, a case was defined as having ≥1 ICD code in the phecode group, and control was defined as having 0 codes in that phecode group. A total of 1,250 phecodes with at least 100 cases were tested for association using a Bonferroni-corrected threshold of $\alpha=0.05/1{,}250=4.00\times10^{-5}$ tests."

Reviewer #2 (Remarks to the Author):

General comments:

This manuscript reports on the largest genome-wide association study (GWAS) of borderline personality disorder (BPD) to date. While the study makes an important contribution to understanding the genetic architecture of BPD, I have identified several methodological concerns that should be addressed before publication.

Response:

We thank the reviewer for the helpful comments.

Comment 1:

1. Incomplete reporting of meta-analysis methodology:

- The authors do not specify whether a fixed-effects or random-effects model was used.
- The choice of model can impact results and interpretation, particularly in the presence of between-study heterogeneity.
- Clarification on the chosen model and assessment of heterogeneity is needed for proper interpretation of the findings.

Response:

We thank the reviewer for the comment and now clarify the used meta analysis method:

Manuscript Changes:

Methods:

> *"Meta-analysis of all samples providing either individual-level data or summary statistics was conducted using the inverse variance weighted fixed effects model in METAL[99] as implemented in RICOPILI[97] with a genome-wide significance threshold of*

5×10^{-8}. To evaluate consistency of effect sizes, we computed Cochran's Q-statistics and the corresponding heterogeneity p-values and I^2 statistics, which quantifies the percentage of total variation due to heterogeneity."

We now also describe in more detail how we filtered for heterogeneity:

Results:

> *"SNP association analysis revealed six independent genome-wide significant loci ($p<5\times10^{-8}$, Fig. 2; Table 1; Supplementary Figs. S2–S14; Supplementary Table S7), and no marker showed significant heterogeneity between studies* after multiple testing correction (smallest heterogeneity p-value=0.023)*."*

Methods:

> "To ensure a robust analysis with highly credible SNP sets, we excluded SNPs with highly significant heterogeneity ($p<0.001$) and SNPs with an effective sample size of less than 85%"

Comment 2:

Absence of power calculations:

- The authors do not present power calculations, despite the substantial case-control imbalance (12,339 cases vs. over 1 million controls).
- Power calculations are essential for evaluating the minimum detectable effect sizes and the impact of the imbalance on the study's ability to detect associations.
- Including power calculations would allow readers to better assess the reliability and interpretability of the findings.

Response:

We now include power calculations and describe the analysis in the results, discussion, and methods sections:

Manuscript Changes:

Results:

"The statistical power of the main analysis to detect genome-wide significant associations ($p<5\times10^{-8}$), was calculated using the Genetic Power Calculator[22]. For a range of allele frequencies (0.01–0.99), and an effect size of 1.1, we calculated the power of the main analysis (N_{eff} = 2×21,617, calculated as described in [21]), and the required sample size for a power over 80% (details see Supplementary Table S6, Supplementary Figure S1)."

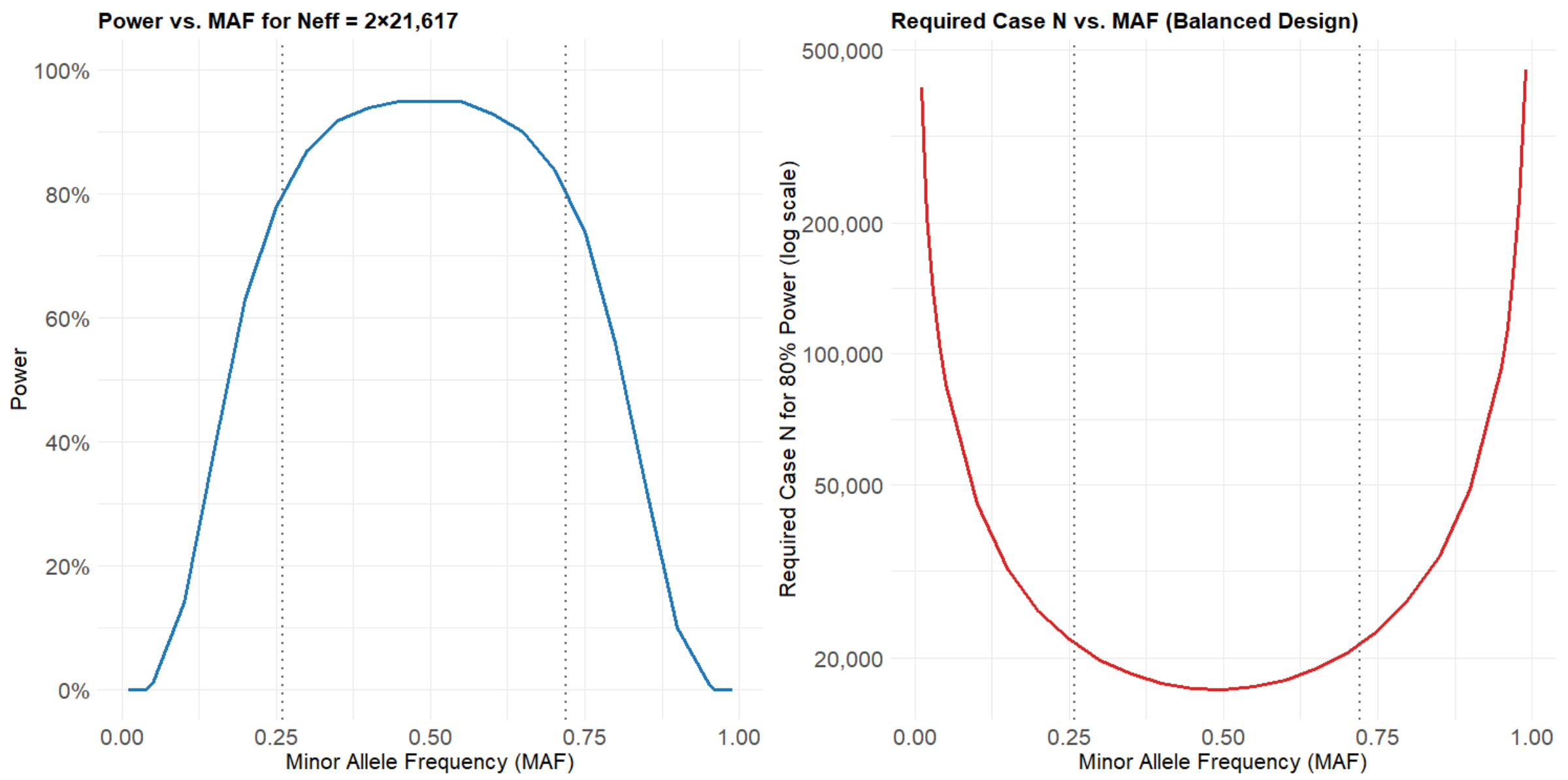

Supplementary Figure S1: Power analysis of the discovery GWAS analysis. The left panel indicates the statistical power to detect effects of 1.1 for the effective N (Neff) of 2x21,617, and in dependence on the effect allele frequency. The right panel indicates the required case N in a balanced design to detect an effect of 1.1 with a power of 80% in depending on the effect allele frequency. Power and the required N were calculated using the Genetic Power Calculator (Purcell, Cherny, and Sham 2003)

Discussion:

"However, the study has several limitations. First, the sample size is still relatively small compared to those of other mental disorders[16], and the power analysis indicated that the statistical power was limited, especially for variants with a lower frequency. As with other mental disorders, the inclusion of additional samples will likely lead to a substantial increase in the number of loci identified[87,88]. "

Methods:

> *"The statistical power of the main analysis to detect genome-wide significant associations ($p<5\times10^{-8}$), was calculated using the Genetic Power Calculator[22]. For a range of allele frequencies (0.01–0.99), and an effect size of 1.1, we calculated the power of the main analysis (N_{eff} = 2×21,617, calculated as described in [21]), and the required sample size for a power over 80% (details see Supplementary Table S6, Supplementary Figure S1)"*

Comment 3:

Lack of independent replication:

The study does not include an independent replication sample to validate the six genome-wide significant loci. While the authors conduct several validity analyses (genetic correlations, PheWAS, polygenic scoring), these do not substitute for formal replication. Given the large sample size, the authors could have held out a portion for validation or used an external dataset (e.g., UK Biobank, deCODE) for replication. Including replication would strengthen the credibility and conclusiveness of the findings.

Addressing these methodological concerns would enhance the rigor, reliability, and impact of the study. I recommend that the authors provide clarification on the meta-analysis model, include power calculations, and consider adding an independent replication sample. With these improvements, the manuscript would make a more substantial contribution to our understanding of the genetic basis of BPD.

Response:

We thank the reviewer for the suggestion. For the initial submission, we had opted to include as many cohorts as possible in the discovery sample to maximize the power of the sample to discover genetic variants. As an exception, we used the sample Spain 2 (repl.) as a replication sample. As the n was limited (46 cases, 435 controls), we only used it to replicate the PGS association, and could demonstrate a significant association of BPD-PGS with BPD case-control status in the sample (3.1% R^2 on the liability scale, p=0.0041, AUC=62.4%). We agree that extending the replication to the SNP level strengthens the findings of the present study. We have now added a second replication cohort, the All of Us cohort (634 cases, n = 98,598 controls), and extended the replication analyses to the SNP-level. We now report the sign tests

for $p<5x10^{-8}$ and $p<10x10^{-6}$, and the associations of the lead SNPs (Table 1) in the replication in the manuscript. We additionally performed a meta-analysis over the combined sample (main analysis + replication sample). In this analysis, the initial six loci remained significant at $p<5x10^{-8}$. Five additional loci showed genome-wide significant associations in the combined analysis and are now reported in the manuscript.

Manuscript Changes:

Results:

> *"To assess replication of the observed associations, the lead SNPs and PGS derived from the main analysis were tested for replication in two independent datasets (Spain 2 & All Of Us; total N_{cases}=685, total $N_{controls}$=107,750). Additionally, SNPs with $p<1x10^{-6}$ in the discovery GWAS analysis were analyzed in a combined GWAS meta-analysis (N_{cases}=13,024, $N_{controls}$=1,149,467)."*
>
> *…*
>
> *"In the sign test all six genome-wide significant lead SNPs showed effects in the same direction in the replication analysis (6/6, p=0.016, Tables 1), and 77% of the lead SNPs associated with $p<1x10^{-6}$ showed effects in the same discretion in the replication analysis (24/31, p=0.0017). In the combined analysis, the six loci remained genome-wide significant (Supplementary Figs. S2–S14), and five additional loci with $p<5x10^{-8}$ were observed (Table 1, Supplementary Figs. S15–S24)."*

Table 1: Lead 11 genome-wide significant SNPs associated ($p<5\times10^{-8}$) with BPD

Locus	CHR	SNP	BP	Locus (start..stop)	A1/ A2	FRQ case	FRQ control	OR	SE	*p*	OR repl.	*p* repl.	OR comb.	*SE comb.*	*p* comb.	Mapped Genes
comb. 1	1	rs2143288	169084340	169082364..169084344	G/A	0.858	0.868	0.89	0.022	1.06×10^{-7}	0.86	0.035	0.89	0.021	**1.23×10^{-8}**	*LINC00970(0.0),LOC101928596(0.0), ATP1B1(0.0),NME7(0.0)*
1	1	rs9970854	191125935	191005935..191148335	G/A	0.595	0.597	0.92	0.015	**1.99×10^{-8}**	0.93	0.183	0.92	0.014	**7.42×10^{-9}**	*LINC01680(14.4)*
comb. 2	2	rs12466671	22583435	22548735..22606235	A/G	0.587	0.599	0.93	0.015	6.80×10^{-7}	0.85	0.002	0.92	0.014	**2.16×10^{-8}**	-
2	3	rs6549383	71266885	71261185..71267720	T/C	0.503	0.503	1.09	0.015	**1.53×10^{-8}**	1.02	0.664	1.08	0.014	**2.45×10^{-8}**	*FOXP1(0.0)*
3	5	rs2135029	155856538	155743538..155868438	A/G	0.594	0.599	0.92	0.014	**1.41×10^{-8}**	0.92	0.141	0.92	0.014	**4.30×10^{-9}**	***SGCD(0.0)***
4	7	rs4727799	114110568	114021868..114224568	T/C	0.663	0.67	1.10	0.015	**6.92×10^{-10}**	1.15	0.015	1.10	0.015	**4.05×10^{-11}**	***FOXP2(0.0)***
comb. 3	7	rs10953781	114974695	114963595..115112695	C/T	0.528	0.534	1.08	0.014	7.13×10^{-8}	1.06	0.313	1.08	0.014	**4.38×10^{-8}**	-
5	9	rs73581580	140251458	140242728..140278158	G/A	0.836	0.839	0.89	0.019	**8.09×10^{-10}**	0.98	0.798	0.89	0.019	**1.45×10^{-9}**	*NRARP(-4.8)*, ***EXD3(0.0),*** *NOXA1(16.4)*
6	12	rs7304862	109988891	114021868..114224568	G/T	0.529	0.513	1.09	0.014	**5.71×10^{-10}**	1.09	0.114	1.09	0.014	**1.68×10^{-10}**	*MYO1H(-52.7), KCTD10 (-23.5), UBE3B(0.0),* ***MMAB(0.0)***, ***MVK(0.0)***
comb. 4	17	rs7210027	65977053	65825053..66098053	T/C	0.77	0.782	0.92	0.017	2.54×10^{-7}	0.87	0.031	0.91	0.016	**3.12×10^{-8}**	*BPTF(0.0),C17orf58(0.0),KPNA2(4.7) ,LINC00674(70.6)*
comb. 5	X	rs5944622	24613189	24578589..24933189	T/C	0.57	0.58	0.93	0.013	9.47×10^{-8}	0.92	0.037	0.93	0.013	**2.05×10^{-8}**	*PDK3 (0), PCYT1B (0), PCYT1B−AS1 (+5.0), POLA1 (+48.8)*

Variants are sorted by chromosomal position. CHR=chromosome; Locus (start..stop)=start and end of the boundaries of the region within r^2 of 0.6 of the index SNP; A1=allele 1, A2=allele 2, FRQ=frequency of A1 in cases/controls; BP=base pair position; comb. = combined analysis; OR=odds ratio of A1; repl. = replication analysis; SE=standard error; SNP=single nucleotide polymorphism; BPD=borderline personality disorder; Mapped genes=genes within r^2 of 0.6 and 50 kb of the index SNP (distance to the 50kb for boundary of the lead SNP in kb is indicated in brackets); Genes in bold were also identified by the gene-based association tests. *P* values in bold indicate genome-wide significance ($p<5x10^{-8}$). OR, SE, and p are reported for the main analysis (N_{cases}=12,339, $N_{controls}$=1,041,717), the replication analysis (repl.) N_{cases}=685, total $N_{controls}$=107,750), and the combined (comb.) meta-analysis (N_{cases}=13,024, $N_{controls}$=1,149,467) of the two datasets.

Discussion:

> "The present study represents substantial progress in the identification of the genetic factors underlying BPD. With a more than 12-fold increase in the number of cases compared to the last GWAS[18], it is the first GWAS of BPD to identify both associated loci with genome-wide significance, and a polygenic signal with substantial prediction of derived PGS. We consider it a key strength of our study that the lead SNP associations and PGS analyses replicate in independent samples, which strengthens the confidence in the validity and generalizability of the genetic findings. However, the study has several limitations."

Methods:

> *"To assess replication of the observed SNP associations, lead SNPs for the thresholds $p<1x10^{-6}$ and $p<5x10^{-8}$ were tested for replication in a meta-analysis of two independent datasets (Spain 2 & All Of Us; total N_{cases} = 685, total $N_{controls}$ = 107,750). Additionally, for SNPs with $p<1x10^{-6}$ in the discovery GWAS analysis, discovery and replication datasets were analyzed in a combined GWAS meta-analysis, and loci with $p<5x10^{-8}$ in the combined analysis are reported."*

Reviewer #3 (Remarks to the Author):

General comments:

A large genome-wide association study performed for borderline personality disorder, a relatively rare psychiatric trait of great interest due to the intersection with many outcomes and behaviors, from depression to suicide to personality. This is an important advance for the study of the underlying genetic architecture of BPD. The biggest concern is the relative lack of power and number of cases recruited.

Comment 1:

Very large number of controls compared to a relatively modest number of cases. It's an important first step and it's good to see that significant SNP heritability is identified (and GWS loci are identified).

Response:

We thank the reviewer for the helpful comments!

Comment 2:

Could more care be taken to match the controls on demographics of the BPD cases, given the massive overrepresentation of controls? I found Tables S1 to be strangely truncated and confusing; can the control exclusion criteria be spelled out more clearly? In the sensitivity analyses, were individuals excluded from both the cases and controls?

Response:

We have now adapted the layout of the table to make it easier to read. Further details on the individual studies can be found in the Supplementary text. We have also added information on age and sex distribution in cases and controls, and on depression comorbidity in the cases to Table S2. While the cohorts that provided summary statistics to the meta-analysis differ in their case-control ratio, we see no evidence of demographic bias within individual cohorts, as indicated by the genomic control lambda values.

For the sensitivity analysis, the 10 cohorts providing summary statistics excluded subjects with SCZ/BIP comorbidity. With respect to the main analysis, for 6 of these 10 cohorts, the controls with SCZ/BIP were already excluded (BioVU, Estonian Biobank, HUNT, MoBa, GLAD, UK Biobank), whereas for 4 of the 10 cohorts, subjects with SCZ/BIP comorbidity were included as a (small) share of the control set for the main analysis (Copenhagen Hospital Biobank and Danish Blood Donor Study; deCODE; Mayo Clinic Biobank; FinnGen). In the individual level data samples, controls were either not screened for SCZ/BIP, or controls with SCZ/BIP were excluded from all analyses. We have now clarified this in the methods section:

Manuscript Changes:

Results:

> *"Details on ascertainment, inclusion, and exclusion criteria for cases and controls of each study are documented in Supplementary Table S1. A summary of the sample sizes, basic demographics (sex and age), depression comorbidity, the total number of tested single-nucleotide polymorphisms (SNPs), as well as quality control, imputation, and analysis details specific to studies sharing summary statistics, are provided in Supplementary Table S2 and the Supplementary Methods."*

Methods:

> *"Furthermore, as a sensitivity analysis, association analyses were performed excluding subjects with a history of BIP or SCZ from the case group in the studies providing summary statistics (Supplementary Tables S3–S5). In the case of the cohorts, where the control population was not filtered for BIP and SCZ in the main analysis (Copenhagen Hospital Biobank and Danish Blood Donor Study; deCODE; Mayo Clinic Biobank; FinnGen), subjects with BIP or SCZ were also excluded from the controls for the sensitivity analysis."*

Comment 3:

Is it too early/ambitious to begin stratifying into sex? The primary analysis already seems to just barely cross the threshold into locus discovery. The female analysis resembles a slightly depowered version of the primary GWAS, with some GWS loci sinking just below the significance threshold as a function of power and not real sex differences.

Response:

We agree with the reviewer, that especially the male-only analysis is still very limited in power. We therefore had initially limited the sex-specific analyses to a restricted set, and did not perform extensive downstream analyses. However, we feel it is the right decision to include the results, and also to make them available for future analyses (e.g. meta-analyses, and sex-specific cross-disorder analyses). In response to comment 8 of reviewer 1, we now added the

SNP-heritability estimates for the analysis. In the revised version, we now clarify and discuss the limited power in more detail (also regarding the limited z-score of the heritability estimate):

Manuscript Changes:

Discussion:

> *"We present the first GWAS to include both sex-stratified and X-chromosome analyses — an initial step towards investigating the sex-specific genetic architecture of the disorder, which is relevant concerning the sex differences in BPD prevalence. We observed a substantially higher SNP heritability in females. Notably, a higher SNP-heritability in females has been described for the two mental disorders that showed the strongest genetic correlation with BPD in our analyses, PTSD, and depression[71]. The lead SNPs of the main GWAS, however, showed the same direction and similar effect sizes in the stratified analyses in both sexes, and the genetic correlation of the sex-stratified GWAS was high and not different from 1. However, it must be noted that the sex-stratified analyses had limited statistical power, particularly the male-only analysis, which was also indicated by a z-score of the SNP heritability under 4[27]. Nevertheless, we consider the sex-stratified analysis an important first step and provide it as a resource for the research community, e.g., for sex-specific meta-analyses or cross-disorder analyses. In the future, sex-stratified analyses in larger samples may help to elucidate sex-specific risk factors of BPD[72]."*

Comment 4:

The male loci are not extremely convincing, particularly the locus on chromosome 2. Why so few variants in LD? Is the lead SNP typed or imputed in most of the samples? Given the relatively small number of loci, regional plots might be useful to readers.

Response:

We thank the reviewer for the comment. Both lead SNPs were imputed in the three genotype samples and two biobank cohorts. For the remaining (seven) cohorts, imputation status is not available. The regional plots were provided in the original submission (Supplementary Figure S22 & S24), and are now added below this answer. We agree that the male results need to be

interpreted with care due to reduced power in the male specific analysis and limited LD-tagging of SNPs, particularly at the chromosome 2 locus. Accordingly, we limited the presentation and discussion of the sex-specific results. We now also added this as a general note to the discussion (see answer to the previous comment). However, we think it is important to provide the results to the interested reader and as a resource for future research.

Manuscript Changes:

Figure:

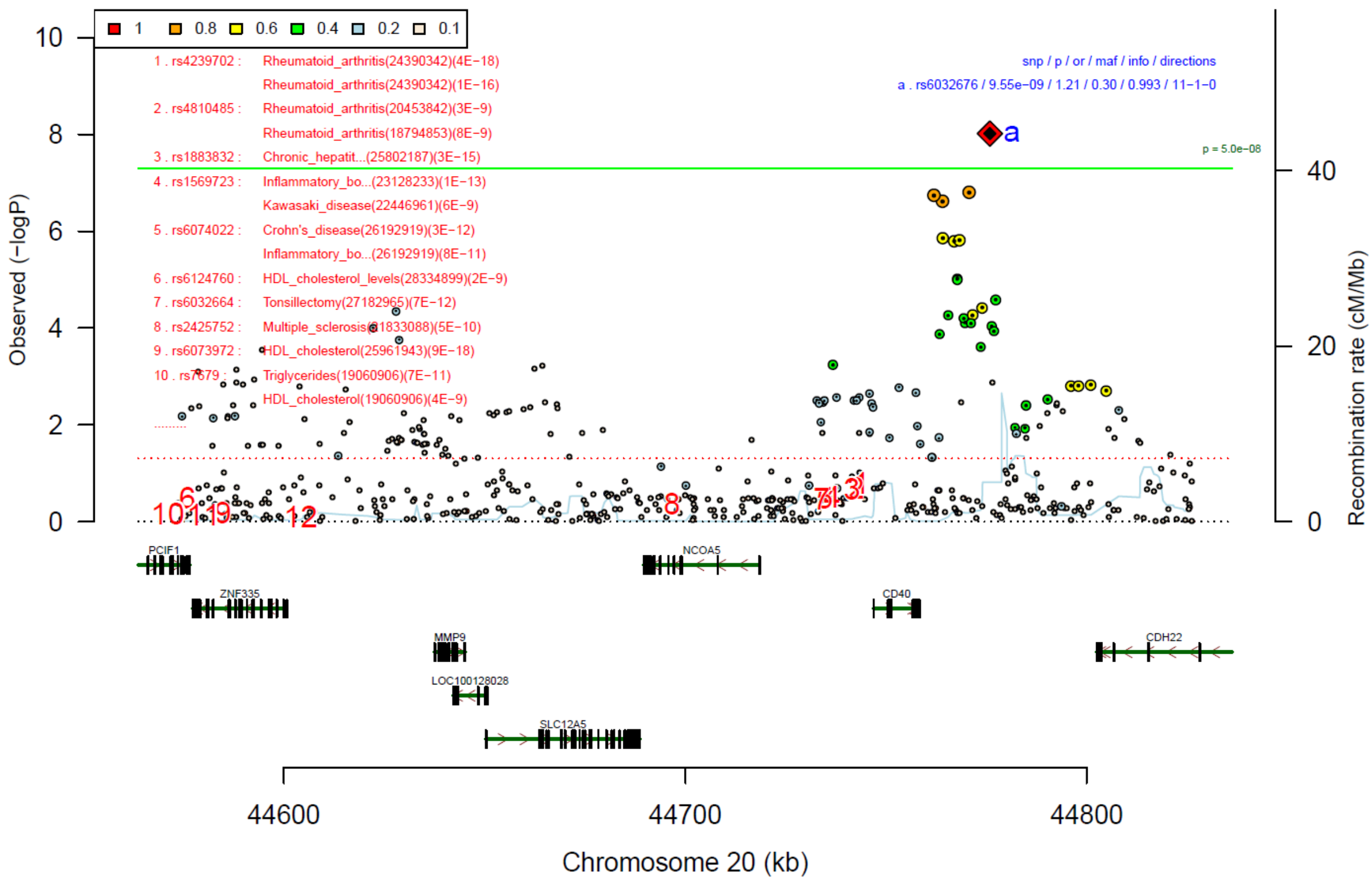

Figure S22: Male subset - region plot of rs6032676. The lead SNP is indicated in diamond shape, and the linkage disequilibrium of the other SNPs (r^2) with the index SNP is represented by the colors. The green line indicates genome-wide significance ($P < 5*10^{-8}$).

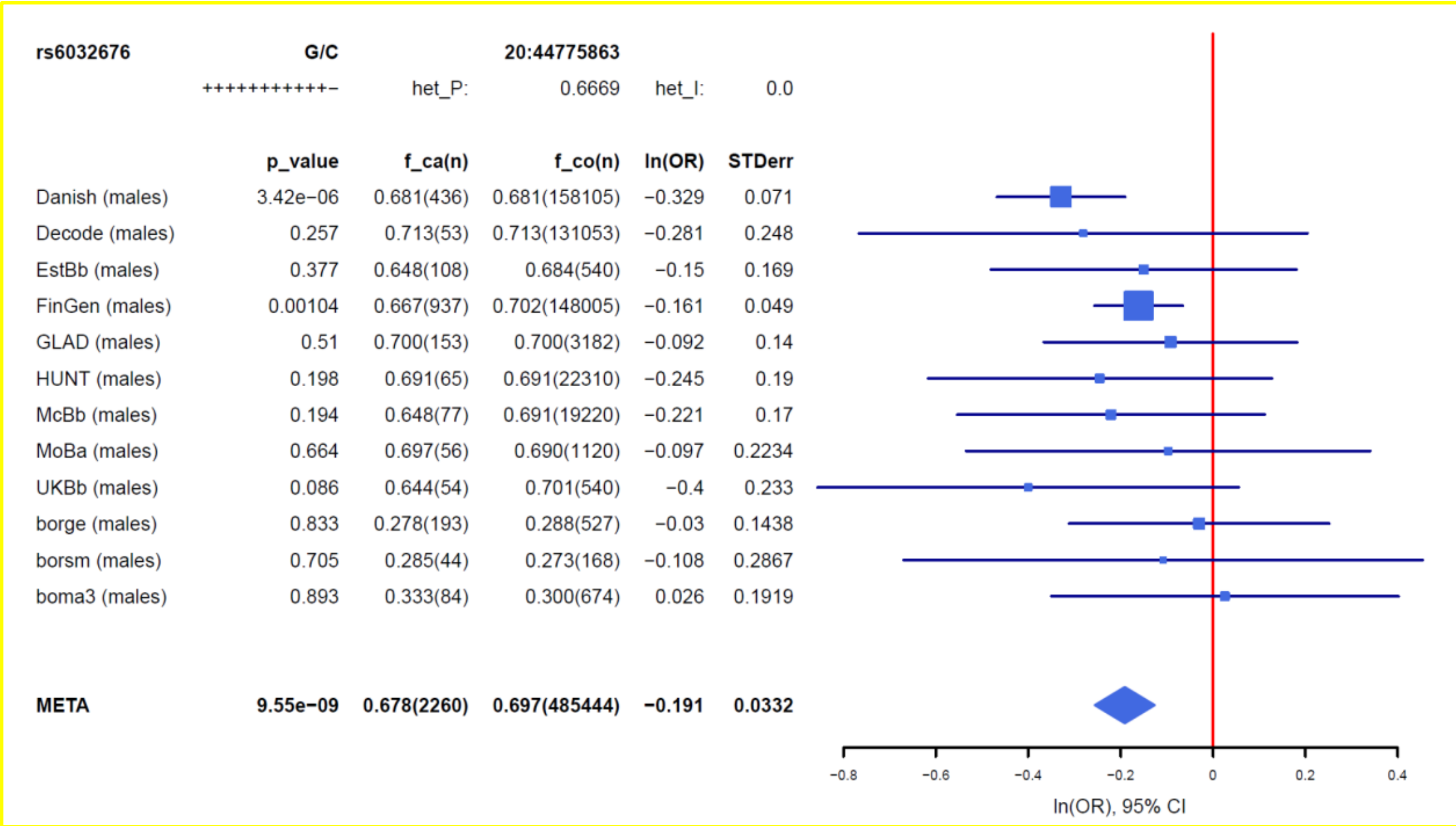

rs6032676	G/C		20:44775863		
	+++++++++++−	het_P:	0.6669	het_I:	0.0
	p_value	f_ca(n)	f_co(n)	ln(OR)	STDerr
Danish (males)	3.42e−06	0.681(436)	0.681(158105)	−0.329	0.071
Decode (males)	0.257	0.713(53)	0.713(131053)	−0.281	0.248
EstBb (males)	0.377	0.648(108)	0.684(540)	−0.15	0.169
FinGen (males)	0.00104	0.667(937)	0.702(148005)	−0.161	0.049
GLAD (males)	0.51	0.700(153)	0.700(3182)	−0.092	0.14
HUNT (males)	0.198	0.691(65)	0.691(22310)	−0.245	0.19
McBb (males)	0.194	0.648(77)	0.691(19220)	−0.221	0.17
MoBa (males)	0.664	0.697(56)	0.690(1120)	−0.097	0.2234
UKBb (males)	0.086	0.644(54)	0.701(540)	−0.4	0.233
borge (males)	0.833	0.278(193)	0.288(527)	−0.03	0.1438
borsm (males)	0.705	0.285(44)	0.273(168)	−0.108	0.2867
boma3 (males)	0.893	0.333(84)	0.300(674)	0.026	0.1919
**META**	**9.55e−09**	**0.678(2260)**	**0.697(485444)**	**−0.191**	**0.0332**

Figure S23: Male subset - forest plot of rs6032676. The plot shows the size and direction of the SNP's effect in each analyzed cohort. Additionally, cohort-specific information and meta-analytic information on the SNP (imputed vs. genotyped SNP (ngt), info score (info), p value, frequency in cases and controls (f_ca(n), f_co(n)) effect size (ln(OR)) and standard error (STDerr)) are given in the table.

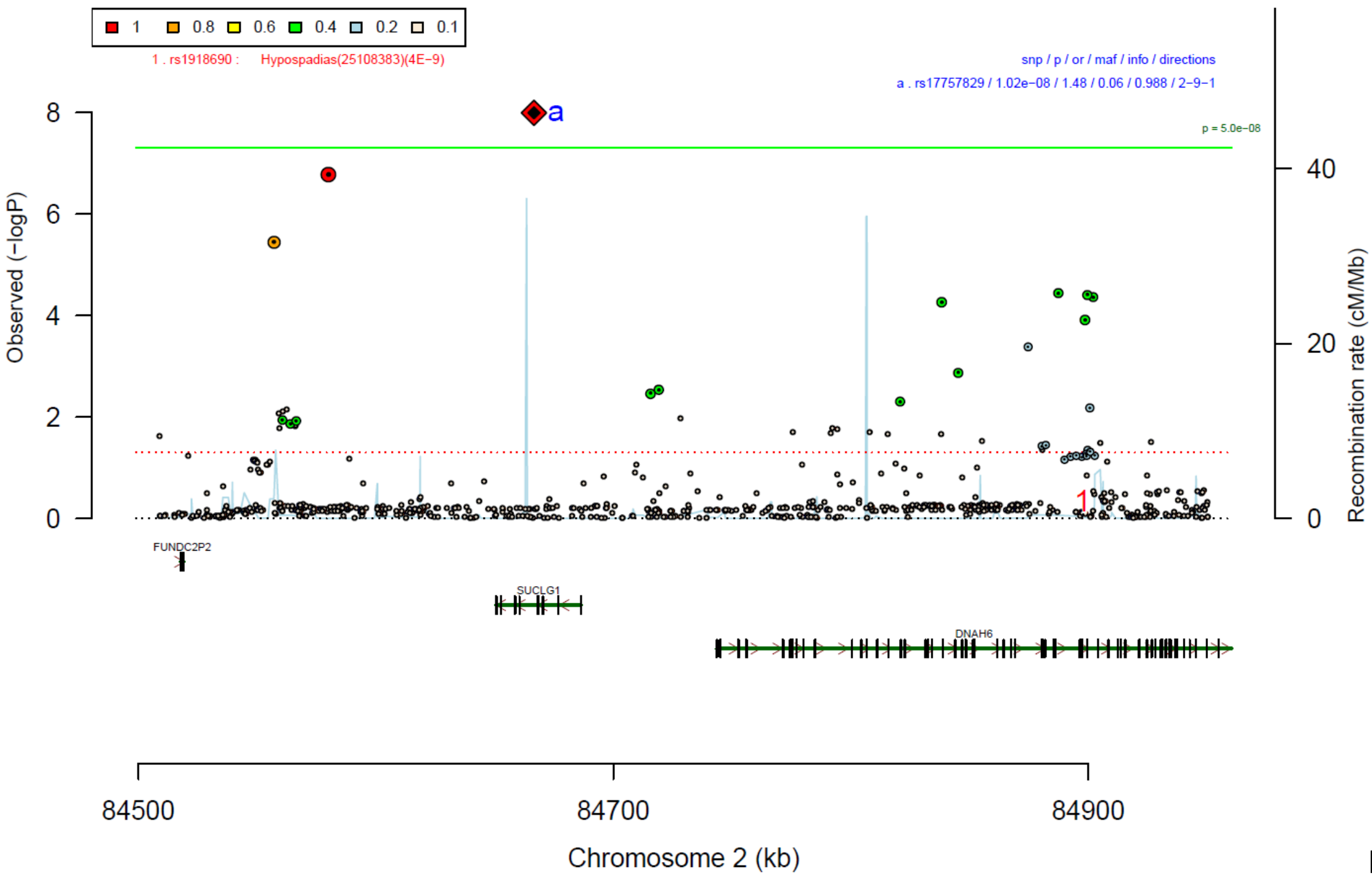

Figure S24: Male subset - region plot of rs17757829. The lead SNP is indicated in diamond shape, and the linkage disequilibrium of the other SNPs (r^2) with the index SNP is represented by the colors. The green line indicates genome-wide significance ($P < 5*10^{-8}$).

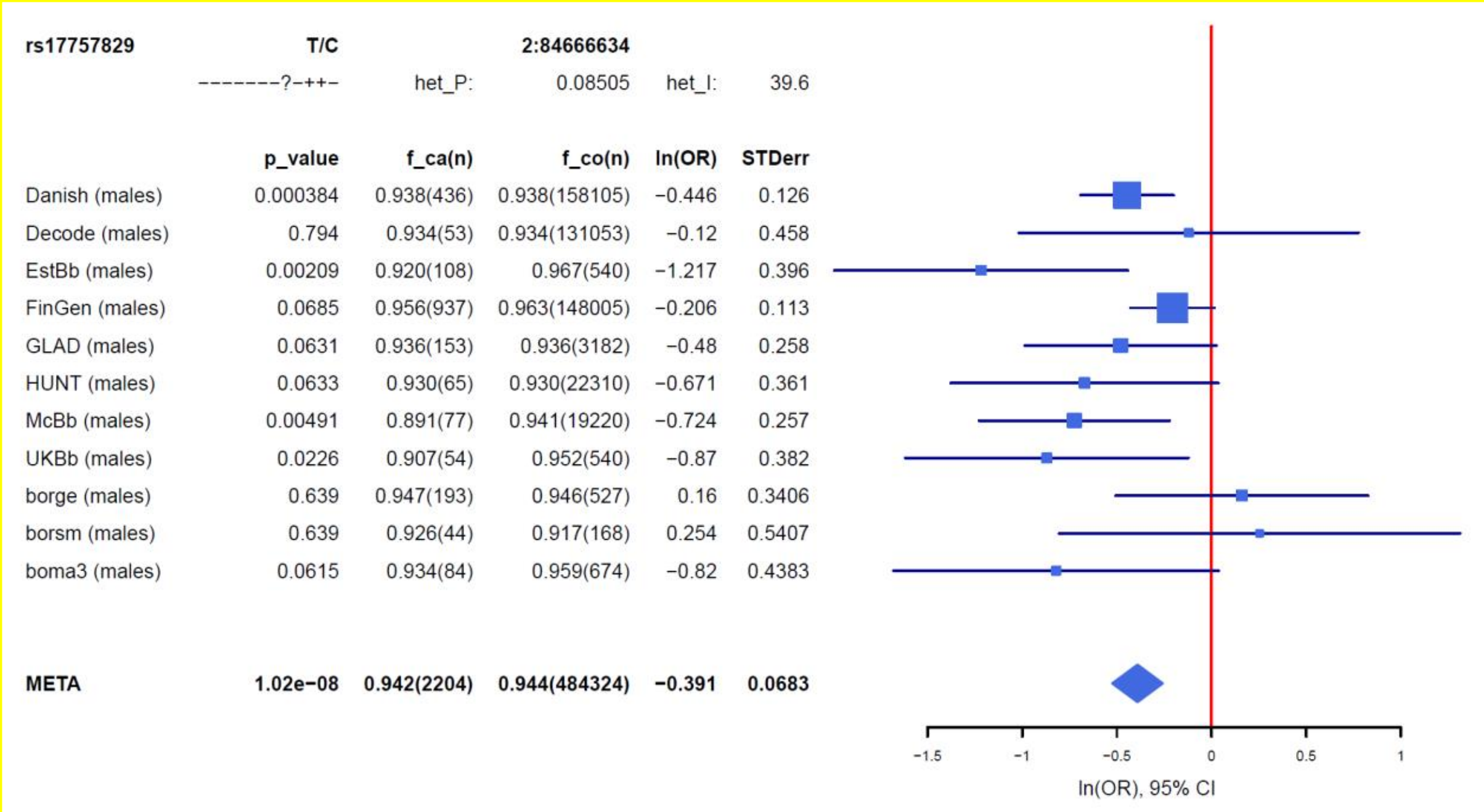

rs17757829	T/C		2:84666634		
	-------?-++-	het_P:	0.08505	het_I:	39.6
	**p_value**	**f_ca(n)**	**f_co(n)**	**ln(OR)**	**STDerr**
Danish (males)	0.000384	0.938(436)	0.938(158105)	−0.446	0.126
Decode (males)	0.794	0.934(53)	0.934(131053)	−0.12	0.458
EstBb (males)	0.00209	0.920(108)	0.967(540)	−1.217	0.396
FinGen (males)	0.0685	0.956(937)	0.963(148005)	−0.206	0.113
GLAD (males)	0.0631	0.936(153)	0.936(3182)	−0.48	0.258
HUNT (males)	0.0633	0.930(65)	0.930(22310)	−0.671	0.361
McBb (males)	0.00491	0.891(77)	0.941(19220)	−0.724	0.257
UKBb (males)	0.0226	0.907(54)	0.952(540)	−0.87	0.382
borge (males)	0.639	0.947(193)	0.946(527)	0.16	0.3406
borsm (males)	0.639	0.926(44)	0.917(168)	0.254	0.5407
boma3 (males)	0.0615	0.934(84)	0.959(674)	−0.82	0.4383
**META**	**1.02e−08**	**0.942(2204)**	**0.944(484324)**	**−0.391**	**0.0683**

Figure S25: Male subset - forest plot of rs17757829. The plot shows the size and direction of the SNP's effect in each analyzed cohort. Additionally, cohort-specific information and meta-analytic information on the SNP (imputed vs. genotyped SNP (ngt), info score (info), p value, frequency in cases and controls (f_ca(n), f_co(n)) effect size (ln(OR)) and standard error (STDerr)) are given in the table.

Comment 5:

It is encouraging to see the consistency of the lead SNPs across the various stratified analyses

Response:

We thank the reviewer for his comment. We have now also added an independent replication cohort, in which all 6 lead SNPs also show effects in the same direction (see the response to Comment 3 of Reviewer 3; Table 1).

Comment 6:

When conducting the liability scale adjustment to the heritability, what was the sample prevalence reported and the sample N provided to LDSC? Was the effective sample size used,

or the raw N?

Response:

We used the effective sample size to calculate the heritability estimates with LDSC, therefore correspondingly used a sample prevalence of 0.5 and the sample size of n = 43,234 for the main analysis. To account for differences in case control ratio of the different included studies, the effective sample size was calculated for each study using the formula NEff = 4 / (1/NCases + 1/NControls), and then these values were summed to obtain the total effective sample size for the respective meta-analysis (as described in Grotzinger, de la Fuente, Privé, Nivard, & Tucker-Drob, 2022; DOI: 10.1016/j.biopsych.2022.05.029).

We clarify this now in the methods section:

Manuscript Changes:

Methods:

> *"SNP heritability, based on the autosomal markers, was estimated using LDSC[23], both as the observed SNP heritability and as the SNP heritability converted to the liability scale, using the N_{eff} (2×21,617=43,234)[21], a corresponding sample prevalence of 0.5, and assuming a population prevalence of 1.5%[3] for the main analysis. For the sex-stratified GWAS, we used sex-specific population prevalences of 2.25% in females and 0.75% in males for the conversion to the liability scale, corresponding to the previously described female:male ratio of 3:1[4,5]. To assess the contribution of confounding to the observed signal, we calculated the attenuation ratio ($\frac{(Intercept - 1)}{(mean\ \chi2^{\blacksquare} - 1)}$)"*

Comment 7:

Reported inflation looks fine and is not a concern. What was the attenuation ratio of the LD score regression?

Response:

For the main analysis, the intercept and SE were 1.0293 (0.0085) and the attenuation ratio was 0.1056 (0.0306). With an intercept in the range of 1.0 - 1.05 and an attenuation ratio of

~.1 we conclude that the large part of our association signal is not attributable to confounding. We now report this in the results, discussion, and methods section:

Manuscript Changes:

Results:

”Linkage disequilibrium score regression (LDSC) was used to estimate SNP heritability and genetic correlations[23]. We observed an SNP-heritability (h_{SNP}^2) of 28.4% (95% confidence interval (CI) [24.9%, 31.8%]), corresponding to an SNP heritability of 17.2% (95%-CI [15.2%, 19.4%]) on the liability scale (assuming a population prevalence of 1.5%) [3]. The LDSC intercept was 1.03 (standard error (SE)=0.0085), with an attenuation ratio of 0.11 (SE=0.031), and we observed a genomic inflation factor (λ_{GC}) of 1.22 (λ_{1000}=1.01).”

Discussion:

“The results indicate that a substantial proportion of the heritability observed in family and twin studies (46–69%)[12,13] can be explained by common genetic variation, as reflected by the estimated SNP heritability of 17%. This is consistent with GWAS in other psychiatric disorders, where SNP heritability is often approximately a third of the estimates from twin and family studies[16]. The substantial contribution of common genetic variation to BPD is also supported by the leave-one-out PGS analyses, in which the PRS-CS predicted a weighted average of 4.6% of the variance on the liability scale. Importantly, the low LDSC intercept (1.02) and the attenuation ratio of 11% indicate that the association signal is largely driven by the polygenic signal and not by confounding.”

Methods:

“SNP heritability, based on the autosomal markers, was estimated using LDSC[23], both as the observed SNP heritability and as the SNP heritability converted to the liability scale, using the N_{eff} (2×21,617=43,234)[21], a corresponding sample prevalence of 0.5, and assuming a population prevalence of 1.5%[3] for the main analysis. For the sex-stratified GWAS, we used sex-specific population prevalences of 2.25% in females and 0.75% in males for the conversion to the liability scale, corresponding to the previously described female:male ratio of 3:1[4,5]. To assess the contribution of confounding to the observed signal, we calculated the attenuation ratio ($\frac{(Intercept - 1)}{(mean\ \chi 2 - 1)}$). “

Comment 8:

Could more be said about the selection criteria and justification for the traits selected for genetic correlations, other than picking a round number (50), with most traits that end up being statistically significant (43)? The selection seems somewhat arbitrary as written.

Response:

We thank the reviewer for the comment. The resulting round number was not intended but occurred by coincidence. We selected phenotypes of interest, i.e. disorders or traits that have been reported to be associated with BPD, e.g. risk factors, or potential comorbid conditions. We decided on a rather targeted approach for the genetic correlations, to contrast the rather exploratory and hypothesis-free PheWAS presented in the same manuscript. We have now added more information on symptom overlap, comorbidity and shared genetic risk to the main text and on the selection criteria to the methods section:

Manuscript Changes:

Main text:

> ***“****Individuals with BPD display high rates of self-harm, suicidal ideation, and suicide attempts. BPD shows substantial symptom overlap and comorbidity with other mental disorders[5,6], and comorbidity with neurological and somatic health conditions[6,7]. While some psychotherapies are effective in treating BPD[8], no psychopharmacological treatments are FDA- approved specifically for BPD[2,9].*
>
> *In addition to environmental risk factors such as early interpersonal trauma[10,11], genetic factors substantially contribute to disorder risk. Twin and family studies estimate the heritability of BPD to be 46–69%[12,13], and demonstrate that the genetic risk for BPD is partially shared with other mental disorders, but as well as with continuous traits, e.g. the Big Five personality traits[14,15]. However, a systematic assessment of shared genetic risk with a broad range of disorders and traits is missing.”*

Methods:

"In a targeted approach, genetic correlations were calculated with 50 GWAS of a range of other disorders and traits relevant to BPD, selected based on reported phenotypic and genetic associations in the literature, theoretical consideration, and data availability. Those included GWAS of mental disorders, suicide, self-harm, trauma, substance use, physical health, pain, sleep, personality traits (Big Five GWAS including data from 23andMe, Inc.), and cognition (Supplementary Table S22). A Bonferroni p-value threshold, corrected for the number of respective tests was applied (α=0.05/50=1×10^{-3})."

Comment 9:

Is the MDD comorbidity known in the cases? Could this be reported?

Response:

We have now compiled information on the comorbidity with depression in BPD cases for all contributing samples. The percentage of BPD cases with MDD ranged from 37-95% with a median of 70%. We now report this in Supplementary Table S2 and refer to it in the main text.

Manuscript Changes:

Results:

"Details on ascertainment, and inclusion and exclusion criteria are documented in Supplementary Table S1. Sample sizes, basic demographics (sex and age), depression comorbidity, and analysis details are provided in Supplementary Table S2 and the Supplementary Methods."

Discussion:

"In BPD, comorbidity with other mental disorders is the rule rather than the exception[1,5], for example, approximately 70% of the investigated BPD patients had a history of depression. Restricting GWAS cases to those without psychiatric comorbidity would reduce the number of available subjects and limit the analysis to a less representative (and drastically smaller) subset. Therefore, we consider it a strength of the present

analysis that the biobanks and cohorts providing summary statistics performed the main analysis without excluding cases with psychiatric comorbidity."

Comment 10:

I appreciate the historical perspective presented in the PRS Figure 3. Trait-specific progress is important to keep in mind in this kind of research.

Response:

We thank the reviewer for the comment.

Comment 11:

Many of the techniques used are well-worn yet appropriate or cutting edge, a good mix of innovative and feasible for this kind of paper.

Response:

We thank the reviewer for the comment.

Comment 12:

Excellent that X chromosome is included in this analysis. It is a critically understudied part of genomic analyses. Was the X chromosome incorporated into any of the heritability calculations?

Response:

We thank the reviewer for the comment. We now clarify this in the description of the heritability calculations.

Manuscript Changes:

Methods:

> "SNP-heritability, based on the autosomal markers, was estimated using LDSC[20], both as the observed SNP-heritability and as the SNP-heritability converted to the liability scale, using the N_{eff} (2×21,617=43,234)[96], a corresponding sample prevalence of 0.5, and assuming a population prevalence of 1.5%[3] for the main analysis."

Comment 13:

Minor: It's standard to refer to X chromosome as '23' for various formatting reasons within the data/plink, perhaps it would be better labeled as 'X' in the plots for readers that aren't accustomed to that convention?

Response:

We agree and have now changed the labeling to "X" in all the figures, and supplementary tables.

Comment 14:

Could more be done to highlight the innovations in the analyses, aside from the largest available sample size? Maybe it makes sense given the available power, but many of the analyses prominently featured in writing or discussion are not particularly novel.

Response:

We thank the reviewer for the suggestion. As the reviewer mentioned in comment 12, we aimed for a balance in using techniques suitable for this investigation. Given that this was the first such analysis for BPD, we included well-established methods which increased confidence in our findings. We now highlight in more detail what we consider the core strengths of the present study, and in which regards the present analyses extend previous approaches, and address gaps in the state of knowledge.

Manuscript Changes:

Introduction:

"In addition to environmental risk factors such as early interpersonal trauma[10,11], genetic factors substantially contribute to disorder risk. Twin and family studies estimate the heritability of BPD to be 46–69%[12,13], and demonstrate that the genetic risk for BPD is partially shared with other mental disorders, but as well as with continuous traits, e.g. the Big Five personality traits[14,15]. However, a systematic assessment of shared genetic risk with a broad range of disorders and traits is missing."

…

"While these findings indicate the potential of using genetic approaches to investigate BPD, research based on those results is limited by the large uncertainties in the estimated effect sizes. Additionally, the extent to which sex-specific genetic effects contribute to the observed sex differences in the prevalence and clinical characteristics of BPD remains unclear."

Discussion:

"Discussion

The present GWAS including 12,339 BPD cases and 1,041,717 controls marks a major advance in BPD genetics, with the demonstration of a substantial SNP-heritability, the identification of genome-wide significant risk loci, pathway and tissue enrichment, and a considerably improved predictive value of derived PGS. The study includes the first sex-stratified and X-chromosomal GWAS for BPD, and the first systematic investigation of shared genetic risk of the disorder. The validity and generalizability of the results is supported by the replication of the lead SNP associations and PGS analyses in independent datasets."

…

"We present the first GWAS to include both sex-stratified and X-chromosome analyses — an initial step towards investigating the sex-specific genetic architecture of the

disorder, which is relevant concerning the sex differences in BPD prevalence and presentation.“

…

“This study is the first to systematically use genetic association data to investigate expression enrichment and potential drug repurposing for BPD, offering initial insights:“

…

“Through genetic correlation and PheWAS analyses, we address a critical gap in the literature by systematically and comprehensively assessing the shared genetic architecture of BPD across a broad spectrum of other disorders and traits.“

Additional remarks:

Reviewer #3 (Remarks on figshare data availability):

Data appears to be as described. Good practice to make the summary statistics available at such an early stage.
